# Global, regional, and national burden of epilepsy, 1990–2021: a Global Burden of Disease study

**DOI:** 10.7189/jogh.16.04066

**Published:** 2026-03-06

**Authors:** Jinqing Zhao, Qiannan Chen, Jiahan Dong, Meng Gao, Siqi Ge, Anxin Wang

**Affiliations:** 1 Department of Epidemiology, Beijing Neurosurgical Institute, Beijing Tiantan Hospital, Capital Medical University, Beijing, China; 2 Department of Clinical Epidemiology and Clinical Trial, Capital Medical University, Beijing, China; 3 Beijing Neurosurgical Institute, Capital Medical University, Beijing, China; 4 Centre for Precision Health, School of Medical and Health Sciences, Edith Cowan; University, Perth, Australia; 5 China National Clinical Research Center for Neurological Diseases, Beijing Tiantan Hospital, Capital Medical University, Beijing, China

## Abstract

**Background:**

Epilepsy is a common neurological disorder characterised by spontaneous seizures without an identifiable cause, leading to high morbidity and a significant public health burden. Understanding trends in its incidence, prevalence, mortality, and disability-adjusted life years (DALYs) is essential for developing effective prevention and treatment strategies. We aim to describe global trends in epilepsy over the past 32 years and explore the impact of sociodemographic index (SDI) levels on disease burden using the Global Burden of Disease 2021 data.

**Methods:**

We analysed the global burden of epilepsy (1990–2019) using age-standardised rates (ASRs) and their 95% uncertainty intervals (UI). We assessed temporal trends with estimated annual percentage change and joinpoint regression. We used decomposition analysis to quantify the effects of population growth, ageing, and epidemiological shifts. Using the Pearson correlation, we examined the relationship between SDI and DALY rates. Additionally, we evaluated global disparities using the slope index of inequality and the concentration index. We used multidimensional health inequality analysis to identify the population with the highest disease burden. Lastly, we used frontier analysis to identify DALY gaps across 204 countries, and the Bayesian age-period-cohort model to predict future burden.

**Results:**

The global burden of epilepsy decreased from 1990 (ASR = 208.1; 95% UI = 163.2, 260.3) to 2021 (ASR = 177.8; 95% UI = 137.7, 225.9). However, there was a gradual increase in incidence from 1990 (ASR = 38.1; 95% UI = 27.9, 49.5) to 2021 (ASR = 42.8; 95% UI = 31.2, 53.7) and prevalence from 1990 (ASR = 287.5; 95% UI = 215.7, 363.0) to 2021 (ASR = 307.4; 95% UI = 234.7, 389.0). The ASR of DALYs was negatively correlated with SDI (R^2^ = 0.619; *P* < 0.001). Furthermore, a multidimensional analysis of health inequalities identified specific groups with a high disease burden. Frontier analysis identified countries and regions that require targeted interventions to address the burden. We projected that the ASDR will continue to decline, with rates dropping to 159.56 (95% UI = 102.26, 216.86) for males and to 109.32 (95% UI = 70.47, 148.17) for females by 2050.

**Conclusions:**

We provide valuable insights into global trends and the burden of epilepsy, emphasising the need for targeted prevention and healthcare strategies across socioeconomic levels.

Epilepsy is a brain disease characterised by recurrent seizures caused by abnormal electrical activity in the brain. Potential causes include stroke, brain trauma, neonatal brain damage, and other unexplained diseases [1]. Despite many potential disease mechanisms that can lead to epilepsy, the cause remains unknown in approximately 50% of cases worldwide [2]. Epilepsy affects more than 70 million people worldwide [3], occurring in all age groups and having a bimodal incidence, with infants and the elderly being at the highest risk. At the same time, epilepsy is the fifth leading cause of neurological disability-adjusted life years (DALYs), accounting for more than 0.5% of global DALYs. In some low-income countries, epilepsy is the second most common cause of neurological diseases [4].

Due to the nature of epilepsy, affected individuals and their families often face stigma and discrimination worldwide. This stems from ignorance, misconceptions, and negative attitudes toward the disease, creating significant challenges for people with epilepsy in many aspects of their lives [5]. Nearly 80% of the world's epilepsy patients live in low-and middle-income countries (LMICs) [6]. Among them, over 75% do not receive appropriate treatment, resulting in a significant treatment gap [7]. However, epilepsy is a treatable disease, especially now that cost-effective treatments exist, and more than 70% of patients can become seizure-free if they have access to aggressive treatment [8,9]. This large treatment gap may be due to a combination of factors, including insufficient capacity of healthcare systems, uneven resource allocation, and insufficient attention to epilepsy care [5]. Diagnosing seizures and epilepsy, as well as identifying their underlying causes, remains a considerable challenge, particularly in LMICs where sociocultural and economic barriers hinder disease awareness and acceptance [10].

In 2015, the World Health Organization (WHO) urged all countries to address the specific needs of people living with epilepsy [11]. In 2019, the WHO released its first global report on epilepsy, ‘Epilepsy: A Public Health Imperative’, emphasising that epilepsy is a critical public health priority [6]. The recently released Intersectoral Global Action Plan on Epilepsy and Other Neurological Disorders 2022–2031 [5] aims to reduce the stigma, impact, and burden of neurological disorders, including associated mortality, incidence, and disability. The plan focuses on enhancing the quality of life for individuals of all ages living with neurological conditions, with a particular emphasis on epilepsy prevention, treatment, and care as critical entry points.

Against this background, we aim to describe the current incidence, prevalence, mortality, and DALYs of epilepsy; analyse the global, regional, and national disease burden attributable to epilepsy from 1990 to 2021; and examine the impact of the sociodemographic index (SDI) on epilepsy outcomes. In addition, we applied the Bayesian age-period-cohort (BAPC) model to project potential trends in epilepsy until 2050. We aim to highlight regional differences in the disease burden of epilepsy, identify high-burden areas, and inform the allocation of medical resources, the enhancement of basic healthcare services, and improved access to epilepsy diagnosis and treatment. We believe that these findings will inspire policymakers to develop scientifically grounded and effective prevention strategies, thereby providing a valuable reference for reducing the global burden of epilepsy.

## METHODS

### Study data

As one of the world's most authoritative epidemiological databases, the Global Burden of Disease (GBD) database has long been a cornerstone of numerous epidemiological studies. The recently released GBD 2021 data cover health losses associated with 371 diseases, injuries, and conditions across 204 countries and regions worldwide, disaggregated by gender, and provide comprehensive, detailed data to support research [12]. We used the GBD 2021 data, including the number of cases, prevalence, deaths, and DALYs for epilepsy with their respective age-standardised rates (ASRs), across three gender groups and six SDI levels (1990–2021), SDI data for 204 countries and regions (1990–2021), and projections of global population changes (2022–2050).

### Definitions

#### Epilepsy

In the GBD results tool, the term describing epilepsy is ‘idiopathic epilepsy’, which refers to a disease characterised by recurrent seizures with no identifiable direct cause. This term aligns with the 1985 recommendations by the International League Against Epilepsy (ILAE) for the classification of epilepsy and epilepsy syndromes [13]. Although the term has been questioned in the latest ILAE classification [14], most epidemiological studies that use GBD data still adopt the older classification. In GBD 2021, the scope of idiopathic epilepsy encompasses all disease types coded as 345–345.9 in the International Classification of Diseases, 9th revision, and all disease types G40.0–41.9 in the 10th revision. Therefore, we used the term ‘epilepsy’.

#### SDI

The SDI is a composite measure designed to provide a comparable indicator of overall socioeconomic development for each country, represented by *per capita* gross domestic product, the average years of schooling for the population aged ≥15 years, and the total fertility rate [15]. Furthermore, GBD 2021 categorises the SDI values of 204 countries and regions into five different groups based on numerical rankings: high SDI, high-middle SDI, middle SDI, lower-middle SDI, and low SDI. In this study, we used SDI to reflect the economic development level of a country or region and analyse the impact of economic factors on epilepsy.

### Statistical analysis

We used ASRs and their 95% uncertainty intervals (UIs) to quantify the incidence, prevalence, mortality, and DALY associated with epilepsy over time, stratified by gender and SDI. We analysed temporal trends in epilepsy from 1990 to 2019 by examining changes in case counts, estimated annual percentage change, and their 95% confidence intervals. Furthermore, we detailed the trends in ASRs across five-year age groups. We used joinpoint regression [16] to estimate the average annual percentage change in incidence, prevalence, mortality, and DALY rates. This method identifies statistically significant inflection points within a given time frame and evaluates overall trends during that period [17,18]. We performed a decomposition analysis to clarify the individual contributions of population growth, population ageing, and epidemiological changes to the burden of epilepsy. We assessed the relationship between SDI and age-standardised DALY rates (ASDRs) using Pearson correlation coefficients. We also assessed global health inequalities in epilepsy using the slope index of inequality and the concentration index. We used multidimensional health inequality analysis to identify specific populations with the heaviest disease burden by integrating three dimensions: SDI, gender, and age. We identified optimal DALY levels for 204 countries and regions across SDI levels using frontier analysis. This analysis identified significant disparities, including 15 countries with the greatest gap in DALYs compared to the global frontier, five countries with the smallest gap in DALYs compared to the frontier for relatively low SDI regions (SDI<0.5), and five countries with the greatest gap in DALYs compared to the frontier for relatively high SDI regions (SDI>0.85). To determine the exact number of countries and regions included in each annotation, we consulted prior literature for validation [19]. Finally, we used the BAPC model to predict the disease burden from 2022 to 2050.

We used *R*, version 4.3.2 (R Core Team, Vienna, Austria) for all statistical analyses, considering a two-sided *P* < 0.05 statistically significant.

## RESULTS

### Spatiotemporal patterns of epilepsy

**Figure 1:**
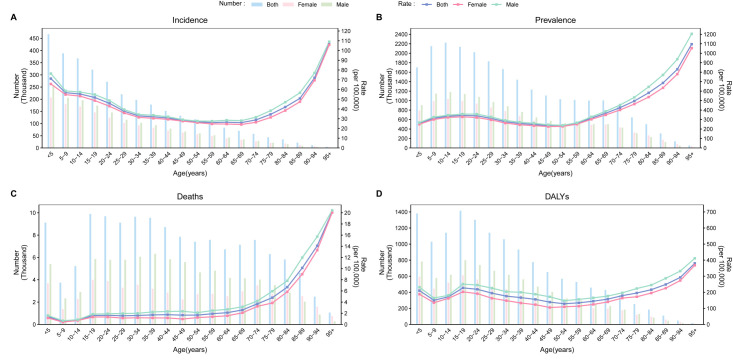
Age and gender differences for epilepsy in 2021. **Panel A.** Incidence. **Panel B.** Prevalence. **Panel C.** Deaths. **Panel D.** DALYs. DALY – disability-adjusted life years.

In 2021, there were 24 million (95% UI = 18.5, 30.7) people suffering from epilepsy and 3.27 million (95% UI = 2.40, 4.13) new cases of epilepsy worldwide (**Table 1**). There were approximately 140 000 epilepsy related deaths and 13.9 million DALYs (95% UI = 10.7, 17.6). In 2021, the global age-standardised incidence rate (ASIR) of epilepsy was 42.8 (95% UI = 31.2, 53.7) per 100 000 population, with rates of 45.1 (95% UI = 33.1, 56.4) in males and 40.5 (95% UI = 29.4, 51.0) in females. The incidence of epilepsy was significantly higher among children aged <5 years and adults aged >70 years. In the elderly population aged >70 years, the incidence rate increased rapidly with advancing age (**Figure 1**). The global prevalence of epilepsy was 307.4 (95% UI = 234.7, 389.0) per 100 000 population, with a prevalence of 321.8 (95% UI = 246.9, 450.1) in males and 293.4 (95% UI = 223.2, 373.0) in females. The prevalence peaked in the 15–19 age group and increased progressively after the age of 50–54 years. The global age-standardised mortality rate (ASMR) was 1.7 (95% UI = 1.5, 1.9) per 100 000 population, with rates of 2.1 (95% UI = 1.8, 2.4) in males and 1.4 (95% UI = 1.0, 1.5) in females. Mortality was the lowest in the 5–9 age group, increased gradually between 50 and 70 years, and rose rapidly after the age of 70 years. The ASDR was 177.8 (95% UI = 137.7, 225.9) per 100 000 population, with rates of 201.3 (95% UI = 157.9, 252.7) in males and 154.2 (95% UI = 114.7, 201.8) in females. The ASDRs peaked among children aged <5 years and adolescents aged 15–19, gradually declined with advancing age, and rose again after 50–54 years. To visually illustrate the disease burden of epilepsy in 2021, we generated a data map depicting ASDRs across 204 countries and regions (Figure S1 in the **Online Supplementary Document**). The burden of DALYs was particularly pronounced in Africa, with Central and Southern Africa experiencing the highest levels.

**Table 1:** Incidence, prevalence, mortality, and DALYs of epilepsy by gender and SDI level, 1990 and 2021

	Incidence			Prevalence			Mortality			DALYs		
	**n (95% UI)**	**ASIR (95% UI)**	**EAPC (95% CI)**	**n (95% UI)**	**ASPR (95% UI)**	**EAPC (95% CI)**	**n (95% UI)**	**ASMR (95% UI)**	**EAPC (95% CI)**	**n (95% UI)**	**ASDR (95% UI)**	**EAPC (95% CI)**
**Global**			0.26 (0.16, 0.36)			0.07 (−0.03, 0.16)			−0.57 (−0.98, −0.17)			−0.61 (−0.84, −0.38)
1990	2 121 189 (1 515 389, 2 784 665)	38.1 (27.9, 49.5)		15 320 820 (11 456 272, 19 419 537)	287.5 (215.7, 363)		104 356 (85 059, 113 885)	2.1 (1.7, 2.3)		11 378 954 (8 902 710, 14 231 577)	208.1 (163.2, 260.3)	
2021	3 272 734 (2 403 802, 4 125 119)	42.8 (31.2, 53.7)		24 220 856 (18 476 943, 30 677 995)	307.4 (234.7, 389)		139 851 (116 953, 153 370)	1.7 (1.5, 1.9)		13 877 827 (10 732 569, 17 619 993)	177.8 (137.7, 225.9)	
**Male**			0.27 (0.24, 0.31)			0.07 (0.01, 0.14)			−0.58 (−0.63, −0.53)			−0.55 (−0.59, −0.52)
1990	1 127 967 (803 892, 1 473 788)	40.2 (29.5, 51.9)		8 031 753 (6 017 720, 10 164 819)	301.6 (227.1, 379.5)		62 706 (50 779, 70 385)	2.6 (2.1, 2.9)		6 394 581 (5 094 694, 7 800 351)	233.7 (187.2, 284.3)	
2021	1 736 615 (1 279 500, 2 176 394)	45.1 (33.1, 56.4)		12 622 148 (9 672 283, 15 882 177)	321.8 (246.9, 405.1)		83 871 (69 903, 94 435)	2.1 (1.8, 2.4)		7 891 536 (6 182 334, 9 856 272)	201.3 (157.9, 252.7)	
**Female**			0.24 (0.21, 0.28)			0.06 (0, 0.11)			−0.58 (−0.64, −0.52)			−0.67 (−0.72, −0.63)
1990	993 222 (711 206, 1 306 451)	36.1 (26.3, 47.1)		7 289 066 (5 410 501, 9 268 891)	274.2 (205.3, 346.8)		41 651 (28 232, 48 921)	1.6 (1.1, 1.9)		4 984 372 (3 595 754, 6 364 531)	182.9 (132.5, 234.6)	
2021	1 536 119 (1 128 507, 1 948 582)	40.5 (29.4, 51)		11 598 708 (8 787 001, 14 784 754)	293.4 (223.2, 373)		55 980 (39 449, 63 074)	1.4 (1, 1.5)		5 986 292 (4 439 424, 7 794 993)	154.2 (114.7, 201.8)	
**High SDI**			0.31 (0.19, 0.44)			0.31 (0.17, 0.45)			0.39 (−0.05, 0.83)			−0.15 (−0.38, 0.08)
1990	390 075 (268 615, 511 963)	47 (32.3, 62.1)		2 874 983 (2 077 734, 3 717 415)	323.5 (234, 417.2)		9610 (9347, 9968)	1 (1, 1)		1 177 932 (844 227, 1 647 445)	133.2 (95.3, 186.4)	
2021	514 418 (342 283, 684 799)	52.1 (34.4, 70.3)		4 192 882 (2 841 951, 5 535 550)	357.3 (242.7, 470.3)		17 447 (15 721, 18 584)	1 (1, 1)		1 466 497 (992 406, 2 150 301)	125.9 (84.9, 188.1)	
**High-middle SDI**			0.11 (0.02, 0.21)			−0.24 (−0.36, −0.12)			−1.84 (−2.3, −1.38)			−1.68 (−1.93, −1.44)
1990	350 385 (246 753, 454 060)	33.4 (23.5, 43.7)		2 771 601 (2 019 357, 3 491 164)	262.3 (192.1, 332.4)		15 683 (14 231, 17 683)	1.5 (1.4, 1.7)		1 783 634 (1 402 274-2 238 661)	168.3 (132.4, 211.4)	
2021	426 210 (289 243, 567 787)	37 (24.9, 50.3)		3 488 443 (2 452 172, 4 627 887)	266.4 (185.6, 353.9)		14 051 (12 417, 15 374)	0.9 (0.8, 1)		1 443 736 (1 021 983, 2 021 723)	112.6 (79.9, 157.1)	
**Middle SDI**			0.27 (0.2, 0.34)			0.03 (−0.04, 0.11)			−1.54 (−1.96, −1.12)			−1.16 (−1.37, −0.95)
1990	665 330 (463 426, 896 157)	36.4 (25.8, 48.4)		4 828 175 (3 502 836, 6 175 502)	279.1 (204, 356.3)		29 033 (24 674, 32 113)	1.8 (1.6, 2)		3 543 162 (2 789 455, 4 471 812)	199.2 (158.1, 250.3)	
2021	964 574 (693 889, 1 233 906)	41.7 (29.8, 53.3)		7 330 502 (5 383 584, 9 313 155)	303 (223.4, 384.6)		28 709 (24 686, 31 674)	1.2 (1, 1.3)		3 519 220 (2 606 430, 4 601 491)	146 (108.4, 192.6)	
**Low-middle SDI**			0.38 (0.27, 0.49)			0.22 (0.14, 0.29)			−0.61 (−0.84, −0.39)			−0.56 (−0.72, −0.4))
1990	464 657 (292 495, 659 137)	36.1 (23.5, 49.7)		3 262 716 (2 121 347, 4 528 532)	277.9 (182, 385.6)		29 745 (20 270, 34 343)	3.1 (2.1, 3.5)		3 061 150 (2 192 306, 3 945 334)	256.3 (185.1, 329.4)	
2021	798 512 (580 952, 1 032 876)	40.9 (30.2, 52.2)		5 744 184 (4 244 875, 7 423 713)	301.7 (224, 388.1)		42 612 (32 664, 48 021)	2.5 (1.9, 2.8)		4 116 015 (3 207 700, 5 233 554)	215.1 (168.2, 270.4)	
**Low SDI**			0.1 (−0.04, 0.24)			−0.1 (−0.2, −0.01)			−0.66 (−1.3, −0.01)			−0.62 (−1.01, −0.23)
2019	248 591 (140 511, 368 640)	42.6 (24.2, 63.4)		1 566 135 (854 321, 2 318 952)	308.5 (169.1, 454.9)		20 198 (16 059, 23 512)	5.7 (4.6, 6.7)		1 802 907 (1 362 683, 2 320 104)	367 (280.9, 467.4)	
2021	566 338 (370 984, 776 083)	45.4 (30.1, 60.7)		3 443 197 (2 342 908, 4 625 232)	308 (214.7, 412.5)		36 906 (29 986, 43 935)	4.6 (3.8, 5.4)		3 320 413 (2 619 841, 4 187 258)	308.1 (246.5, 378.6)	

ASDR – age-standardised DALY rate, ASIR – age-standardised incidence rate, ASMR – age-standardised mortality rate, ASPR – age-standardised prevalence rate, CI – confidence interval, DALY – disability-adjusted life-year, EAPC – estimated annual percentage change, SDI – sociodemographic index

### Association with the SDI

From 1990 to 2021, the incidence of epilepsy showed an increasing trend across all five SDI levels (Figure S2 in the **Online Supplementary Document**). The highest incidence was observed in high SDI regions, followed by low SDI regions. Similarly, the prevalence of epilepsy also increased across all six SDI levels, with the highest prevalence in high SDI regions and the smallest increase in prevalence at the high-middle SDI level. Regarding mortality rates, epilepsy patients in low SDI regions had the highest mortality, followed by low-middle, middle, and high-middle SDI regions. Interestingly, mortality rates in high-SDI regions showed a slight upward trend. The DALYs attributable to epilepsy showed a downward trend across all five SDI levels, with a weak downward trend at the high SDI level and significant downward trends at the other five levels (**Table 1**).

To further illustrate these results, we created data maps for 204 countries and regions (Figure S3 in the **Online Supplementary Document**), showing an increase in epilepsy incidence over 32 years in East Asia, Southeast Asia, North America, Africa, much of Europe, and southern South America. Equatorial Guinea experienced the largest increases in both incidence and prevalence worldwide. Most countries with increasing incidence also showed an increase in prevalence. Despite the general trend of declining mortality from epilepsy worldwide, mortality rates are still gradually increasing in some regions, with the largest increases in Japan and parts of the Mediterranean. Only 43 countries worldwide have increased ASDRs for epilepsy; all other countries, except Uzbekistan (0% increase), have decreased DALY rates (Tables S1–4 in the **Online Supplementary Document**). The expected value of ASDRs decreased with increasing SDI, and increased slightly at an SDI of 0.5. Most regions have declined steadily over time, with values close to expectations. Rates in western sub-Saharan Africa and Oceania remained essentially unchanged over the 32 years, whereas rates in Central Asia and southern sub-Saharan Africa increased and then decreased over the period.

**Figure 2:**
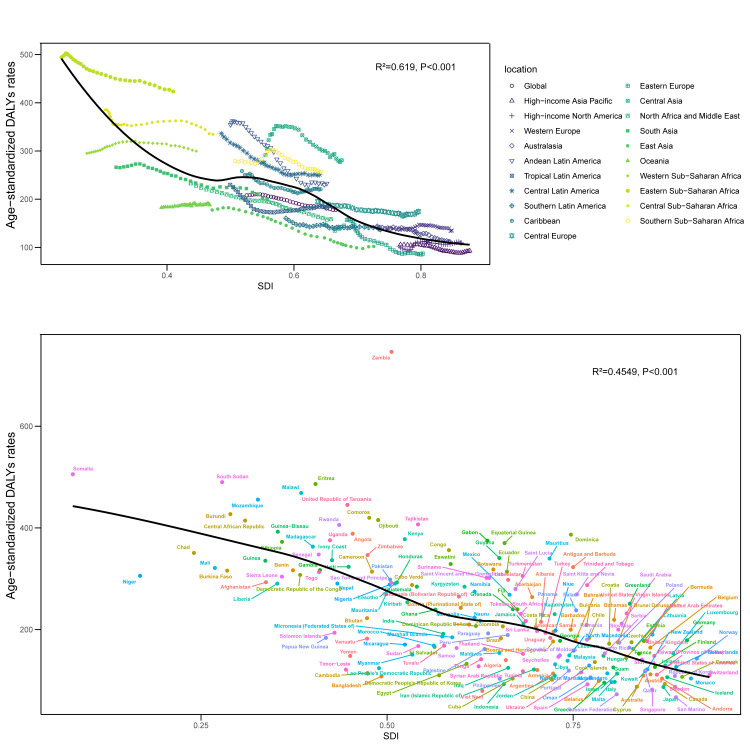
Association between ASDRs and SDI for epilepsy. **Panel A.** ASDRs by SDI across different regions. **Panel B.** Scatter plot of the correlation between ASDRs and SDI. ASDR – age-standardised disability-adjusted life year rate, SDI – sociodemographic index.

To further explore the correlation between SDI level and ASDR, we conducted Pearson correlation analysis (**Figure 2**, Panels A and B), which showed that epilepsy was negatively correlated with SDI both across regions (R^2^ = 0.619; *P* < 0.001) and across countries (R^2^ = 0.455; *P* < 0.001). Additionally, we fitted an expected-value curve for ASDRs in a country or region. As SDI increased, the ASDRs gradually decreased, indicating that the disease burden caused by epilepsy decreased with higher SDI.

### Decomposition analysis

To explore the impact of population growth, population ageing, and epidemiological changes on epilepsy DALYs from 1990 to 2021, we conducted a decomposition analysis. We found that these three factors contributed to ASIRs, age-standardised prevalence rates (ASPRs), ASMRs, and ASDRs across regions with varying SDI levels (Figure S4 in the **Online Supplementary Document**). The results indicate that population growth is the primary driving factor globally and in low SDI regions, particularly in ASIRs and ASPRs. In contrast, in high SDI regions, epidemiological changes have a greater impact on ASMRs and ASDRs. Additionally, ageing plays a crucial role in the increase of mortality and disease burden in high-SDI regions. Overall, the disease burden in low SDI regions is predominantly driven by population growth, whereas in high SDI regions it is more influenced by epidemiological changes and ageing.

### Health inequality analysis

**Figure 3:**
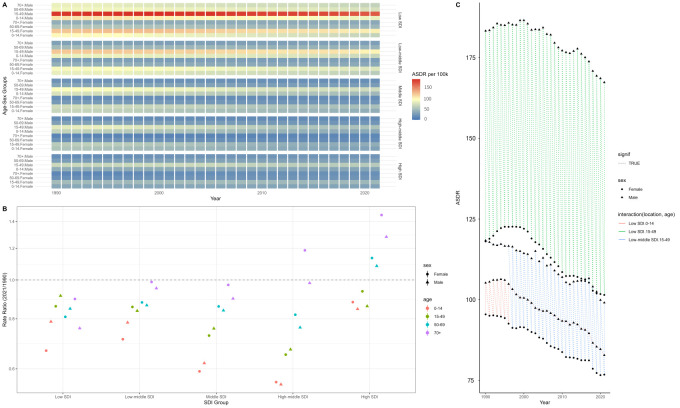
Multidimensional health inequality analysis of SDI, age, and gender (1990–2021). **Panel A.** Heat map distribution of ASDR by age, gender, and SDI. **Panel B.** Scatter plot of RR by age, gender, and SDI. **Panel C.** Temporal and spatial evolution of the top five highest disease burdens. ASDR – age-standardised disability-adjusted life year rate, DALY – disability-adjusted life year, SDI – sociodemographic index, RR – rate ratio.

Building on the conclusion that epilepsy DALYs are associated with SDI, we conducted a health inequality analysis to examine the ASDRs across different SDI levels (Figure S5 in the **Online Supplementary Document**). As SDI increased, the ASDRs gradually decreased. The inequality slope index for epilepsy decreased from −187.07 (95% UI = −150.69, 223.46) in 1990 to −200.61 (95% UI = −168.52, 232.71) in 2021, while the concentration index increased from −0.1488 in 1990 to −0.1947 in 2021. These results highlight the growing health inequalities in epilepsy, with low SDI regions continuing to bear a higher disease burden – a trend that became more pronounced in 2021. To reveal significant cross-inequality characteristics in the global distribution of the burden of epilepsy, we also conducted a multidimensional health inequality analysis. The results showed that countries with low SDI had a persistently high burden, which was significantly higher than that in the high-SDI group. The greatest burden of disease occurred in males aged 15–49 years in areas with low SDI (**Figure 3**, Panel A). The burden of disease did not change significantly between 1990 and 2021 for most groups (**Figure 3**, Panel B). In the high SDI group, the rate ratios (RRs) – calculated as the 2021 ASDR divided by the 1990 ASDR for each age and sex group – were higher among both males (RR = 1.28; 95% confidence interval = 0.71, 2.30) and females (RR = 1.45; 95% confidence interval = 0.81, 2.61) in the ≥70-year group. Since the ASDR in males was higher than that in females of the same age in 2021, the disease burden in this group will increase at the same time, and the gap will narrow or even exceed over time. Middle-aged individuals in low SDI areas consistently had the highest disease burden (**Figure 3**, Panel C). Since 1997, the disease burden of males aged 15–49 years entered the top five, while that of males aged 0–14 years dropped out of the top five.

### Frontier analysis

To explore the ideal conditions under which countries can control the disease burden of epilepsy based on their corresponding SDI levels each year, we conducted a frontier analysis (Figure S6 in the **Online Supplementary Document**). In the frontier analysis results, the five countries shown in blue (*i.e.* Bangladesh, Cambodia, Timor-Leste, Niger, and Somalia) have low SDI levels but are close to the frontier, indicating that, although they have relatively low SDI levels, they have achieved significant progress in the prevention and treatment of epilepsy. The five countries marked in red (*i.e.* Norway, Luxembourg, Belgium, Lithuania, and Germany) have high SDI levels but are farthest from the frontier, meaning that despite their higher economic development, their effectiveness in epilepsy prevention and control is relatively poor. The fifteen countries furthest from the frontier are marked in black, including South Sudan, Eritrea, Malawi, the United Republic of Tanzania, Mozambique, and others.

### Projections of epilepsy DALYs up to 2050

We used the BAPC model, combined with population projections from 2024 to 2050, to predict changes in the incidence, prevalence, mortality, and DALYs of epilepsy in male and female populations by 2050 (Figure S7 in the **Online Supplementary Document**). For both males and females, incidence and prevalence rates showed a slight upward trend, whereas mortality and DALY rates showed a downward trend.

## DISCUSSION

We used the GBD 2021 data to analyse the epidemiological trends of the disease burden caused by epilepsy from 1990 to 2021, incorporating factors such as gender, age, and SDI, with the goal of providing a comprehensive description of the prevalence trends of epilepsy and the burden of epilepsy across regions, contributing to the understanding and management of epilepsy. Over the past 32 years, deaths and DALY caused by epilepsy have significantly decreased, reflecting improvements in the accessibility of current treatments. However, despite these advances, we found that the incidence and prevalence of epilepsy continue to rise across all SDI levels. This trend underscores the ongoing challenge of addressing the global burden of epilepsy, especially in LMICs where access to effective treatment remains limited.

Age is commonly associated with the incidence and prevalence of epilepsy, consistent with previous descriptive reports [20]. We found that the incidence of epilepsy follows a bimodal distribution, with higher rates observed in both the youngest and oldest age groups. In individuals aged >70 years, the incidence of epilepsy increases with age. The prevalence of epilepsy shows an upward trend in adolescents and further increases after the age of 50. Some studies suggest that in developed countries, the prevalence of epilepsy increases with age after 50, whereas in developing countries, it decreases after 50 and then rises again after 60 years [21–23]. This difference may be attributed to variations in healthcare standards driven by economic levels. The mortality and DALY rates for epilepsy reach their lowest point at ages 5–9 and peak at ages 15–19. According to the 2016 global years lived with disability and years of life lost rates due to epilepsy by age [4], we infer that DALYs in the 5–9 age group are primarily driven by years lived with disability, while years of life lost play a decisive role in the DALYs for the 15–19 age group. Age five represents a peak period for epilepsy onset in children, during which the nervous system is still underdeveloped. This makes functional impairments caused by epilepsy more severe, significantly increasing years lived with disability. In contrast, at ages 15–19, status epilepticus likely contributes to the higher years of life lost observed.

In terms of gender differences, rates are significantly higher among males than among females. Although both mortality rates and DALYs show a downward trend, the estimated annual percentage change for female mortality and DALYs has decreased more significantly, indicating a more pronounced decline in epilepsy among females. Some suggest that females living in countries where marriage is considered unsuitable or where they are socially marginalised are more likely to conceal their diagnosis or medical history [20]. Clinical observations and translational studies have indicated that many characteristics of epilepsy are significantly influenced by gender [24–26]. Studies also suggest that the common pathological features of epilepsy syndromes are related to gender differences [27]. Irfan and colleagues [28] suggest that epigenetic mechanisms are closely related to the pathogenesis of epilepsy, with the biased deployment of epigenetic factors and mechanisms in males and females being a fundamental cause of gender differences in the risk, onset, and progression of epilepsy. However, it should be emphasised that these observations are drawn from the literature and remain speculative, as they are not directly supported by the GBD data. Further research into these gender differences in epigenetic mechanisms is needed to better understand the pathogenesis and progression of epilepsy, as well as to identify new, more effective, and personalised diagnostic, preventive, and therapeutic strategies.

We found that as SDI increases, the ASDRs for epilepsy gradually decrease, indicating a negative correlation between the two. This negative correlation between epilepsy burden and SDI is consistent with other published reports [29–31]. However, we found no correlation between incidence and prevalence rates and SDI levels (**Figure 2**), consistent with previous studies [32]. While mortality rates were negatively correlated with SDI, some high SDI regions, such as Japan, Greece, and Italy, still experience an increase in epilepsy mortality. Our decomposition analysis showed that mortality in high SDI regions is strongly influenced by population ageing. The results suggest that this demographic shift could be a key factor driving the rising mortality rates in these countries. At all SDI levels, the ASDRs for epilepsy were lowest in high SDI regions and highest in low SDI regions. We also found that ASDRs in low SDI regions decrease more rapidly than at other SDI levels, and the gap between low and high SDI regions is narrowing, indicating a reduction in health inequality related to epilepsy. In 2021, the global disease burden remained concentrated in countries with lower SDI, and although health inequalities between countries have decreased, the gap remains significant. The multidimensional health inequality analysis not only verified the persistence of SDI gradient differences (*i.e.* ASDR in low SDI countries is higher than in high SDI countries) but also found the formation mechanism and dynamic evolution of the superimposed effect of disadvantage, which has important implications for the development of global epilepsy prevention and control strategies. According to a report by the WHO in 2022 [33], the diagnosis rate of epilepsy in low SDI countries is less than one-third of that in high SDI countries, which may lead to delayed treatment and chronic disease, leading to a high disease burden. Although there were no significant temporal changes in the burden of disease for most groups (Figure S8 in the **Online Supplementary Document**), the increase in RRs among people aged >70 years in high SDI countries suggests the emergence of new risks associated with ageing. It is worth noting that the phenomenon that the burden increased faster among females than among males in the high SDI group may be closely related to the high rate of living alone and insufficient social support among older females. This suggests the need to develop sex-specific care models for older age groups.

The 2019 Global Epilepsy Report highlights the severe underinvestment in epilepsy research globally, particularly in countries with lower SDI [6]. The Intersectoral Global Action Plan calls on member states to develop and strengthen epilepsy care, increase the accessibility and affordability of antiepileptic drugs, and ensure that the needs of people with epilepsy and their families are reflected in health policies [5]. While many countries have implemented effective interventions and made some progress, there remains a need for a more rational allocation of healthcare resources. Therefore, we conducted a frontier analysis to provide insights and references for epilepsy prevention and treatment across 204 countries and regions worldwide. In countries with lower SDI levels and smaller gaps to the frontier, health investments in epilepsy treatment may be reduced, as these countries have already made significant progress in controlling the disease burden. Public health investments in these countries should be redirected to other areas. Conversely, for countries with higher SDI levels and a larger gap from the frontier, increased health economic investments in epilepsy prevention and treatment are needed, as the disease burden remains substantial even in wealthier regions. For instance, Bangladesh and Cambodia have effectively leveraged community health workers to integrate epilepsy care into primary healthcare. In Bangladesh, the community health worker programme delivers education, screening, and management services in rural communities, improving access to treatment and patient outcomes [33]. In Cambodia, community health volunteers conduct patient identification, follow-up, and adherence support, substantially reducing the treatment gap and enhancing seizure control and quality of life [34–36]. These examples provide actionable insights for policymakers, illustrating how community health worker training, community-based follow-up, and integration into primary care can replicate high-performing outcomes identified in frontier analyses. For all countries with the largest gap from the frontier in terms of epilepsy DALY, stronger efforts should be made in epilepsy prevention and treatment, with health policies tailored to each country's specific needs. According to the BAPC model, the ASIRs and ASPRs for epilepsy may increase slightly in both males and females, whereas the ASMRs and ASDRs are expected to decline.

Several limitations must be acknowledged. First, we focused specifically on epilepsy rather than all subtypes, following the GBD definition of ‘idiopathic epilepsy’. However, this classification does not fully align with the current ILAE taxonomy and may introduce misclassification bias despite standardised adjustments applied in the GBD framework. Based on the definitional differences, the GBD ‘idiopathic epilepsy’ category may capture some cases that would be classified as symptomatic or structural epilepsy under the current ILAE system, potentially leading to a slight overestimation of epilepsy burden in settings where diagnostic imaging and etiological workup are limited. Conversely, certain ILAE-defined subtypes, such as genetic generalised epilepsies or focal epilepsies without clear aetiology, may be underrepresented in GBD estimates, possibly causing underestimation in populations where these subtypes are prevalent. Therefore, the overall impact on disease burden may vary by region and diagnostic capacity, underscoring the need for caution when comparing GBD-based estimates with studies using contemporary ILAE definitions. Second, our decomposition analysis is restricted to three components – population growth, population ageing, and epidemiological changes – as standardised in the GBD framework. While this approach ensures global comparability, it does not fully capture the impact of healthcare system development, diagnostic improvements, treatment accessibility, or public health interventions, which are likely important contributors to changes in epilepsy burden. Third, we examined inequality patterns by SDI and did not include specific modifiable health system factors, such as antiepileptic drug availability or neurologist density, because the GBD framework lacks globally comparable data. These unmeasured factors may partly explain why some low SDI countries performed better than expected in the frontier analysis. Additionally, the BAPC model we applied extrapolates from historical age, period, and cohort patterns, assuming that past trends will continue. While this standardised approach within the GBD framework ensures comparability across regions, it does not explicitly account for potential healthcare disruptions, investment scenarios, or emerging risk factors such as climate change-related infections. Therefore, our projections should be interpreted as a baseline scenario rather than forecasts conditioned on future system or environmental changes. Furthermore, as the GBD 2021 framework does not separately account for secondary impacts related to the COVID-19 pandemic, we could not directly quantify the extent to which disruptions in healthcare services, interruptions in medication supply chains, and limitations in outpatient care may have contributed to the underdiagnosis or underreporting of epilepsy cases. Lastly, the data sources in the GBD database may vary by the level of health statistics available in different countries and regions. Some remote areas may lack direct data and instead rely on model-based estimations.

## CONCLUSIONS

Although the global burden of epilepsy has declined since 1990, the increasing incidence and prevalence indicate a persistent and evolving public health challenge. The strong negative association between disease burden and socioeconomic development highlights substantial health inequalities across countries and populations. Our findings underscore the need for targeted, context-specific interventions in high-burden regions, particularly those with lower SDI, to improve prevention, diagnosis, and long-term management of epilepsy. Future research should focus on identifying modifiable risk factors driving the rising incidence and evaluating the effectiveness of health system and policy interventions aimed at reducing disparities and sustaining the projected decline in epilepsy-related disability and mortality.

## Additional material


Online Supplementary Document
