# Supplementary material for: Global, regional, and national burden of epilepsy, 1990–2021: a Global Burden of Disease study
Source: J Glob Health. 2026 Mar 6;16:04066. doi: 10.7189/jogh.16.04066 (PMC12964325; doi:10.7189/jogh.16.04066)
Supplement: Online Supplementary Document [file jogh-16-04066-s001.pdf]

Supplement to: Zhao J, Chen Q, Dong J, Gao M, Ge S, Wang A. Global, regional, and national, burden of epilepsy, 1990–2021: a Global Burden of Disease Study. J Glob Health. 2026;16:04066.

Figure S1. DALYs for epilepsy in 2021 Across 204 Countries and Territories Worldwide.

Figure S2. Trends of epilepsy Across Different SDI Regions (1990–2021). SDI, Sociodemographic Index; DALY, Disability-Adjusted Life-Years. (A) Age-Standardized Incidence Rate Trends in Different SDI Regions for epilepsy. (B) Age-Standardized Prevalence Rate Trends in Different SDI Regions for epilepsy. (C) Age-Standardized Mortality Rate Trends in Different SDI Regions for epilepsy. (D) Age-Standardized DALY Rates Trends in Different SDI Regions for epilepsy.

Figure S3. AAPCs of epilepsy in 204 Countries Worldwide. AAPC: Average Annual Percentage Change. (A) AAPCs of Age-Standardized Incidence Rates in 204 Countries. (B) AAPCs of Age-Standardized Prevalence Rates in 204 Countries. (C) AAPCs of Age-Standardized Mortality Rates in 204 Countries. (D) AAPCs of Age-Standardized DALY Rates in 204 Countries.

Figure S4. Decomposition Analysis of the Trends in epilepsy DALYs from 1990 to 2021. (A) Decomposition Analysis of the Trend in Age-Standardized Incidence Rate from 1990 to 2021 for epilepsy. (B) Decomposition Analysis of the Trend in Age-Standardized Prevalence Rate from 1990 to 2021 for epilepsy. (C) Decomposition Analysis of the Trend in Age-Standardized Mortality Rate from 1990 to 2021 for epilepsy. (D) Decomposition Analysis of the Trend in Age-Standardized DALY Rates from 1990 to 2021 for epilepsy.

Figure S5. Slope Indexes and Concentration Indexes for epilepsy DALYs from 1990 to 2021 Worldwide. DALY, Disability-Adjusted Life-Years. (A) The Slope Index of Inequality for Age-Standardized DALY Rates. (B) Concentration Index for Age-Standardized DALY Rates.

Figure S6. Frontier Analysis Based on SDI and epilepsy DALYs in 204 Countries and Territories. SDI, Sociodemographic Index; DALY, Disability-Adjusted Life-Years. (A) The Frontier Analysis Based on Age-Standardized DALY Rates and SDI from 1990 to 2019. (B) The Frontier Analysis Based on Age-Standardized DALY Rates and SDI in 2021.

Figure S7. Temporal Trends Age-Standardized DALY Rates for epilepsy at the Global Level in Different Genders from 1990 to 2050. (A) Temporal Trends of Age-Standardized Incidence Rate in Male from 1990 to 2050. (B) Temporal Trends of Age-Standardized Incidence Rate in Female from 1990 to 2050.

Table S1. AAPC Results for Incidence.

Table S2. AAPC Results for Prevalence.

Table S3. AAPC Results for Mortality.

Table S4. AAPC Results for DALYs. DALY, Disability-Adjusted Life-Years.

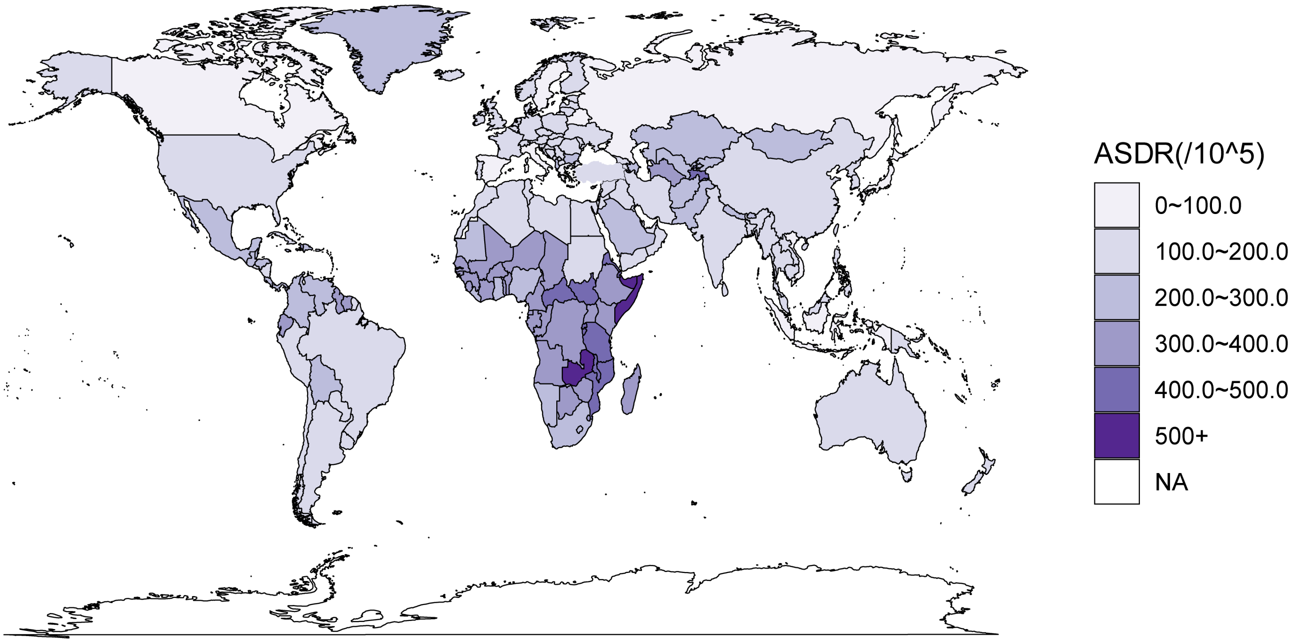
ASDR(/10^5)
0~100.0
100.0~200.0
200.0~300.0
300.0~400.0
400.0~500.0
500+
NA

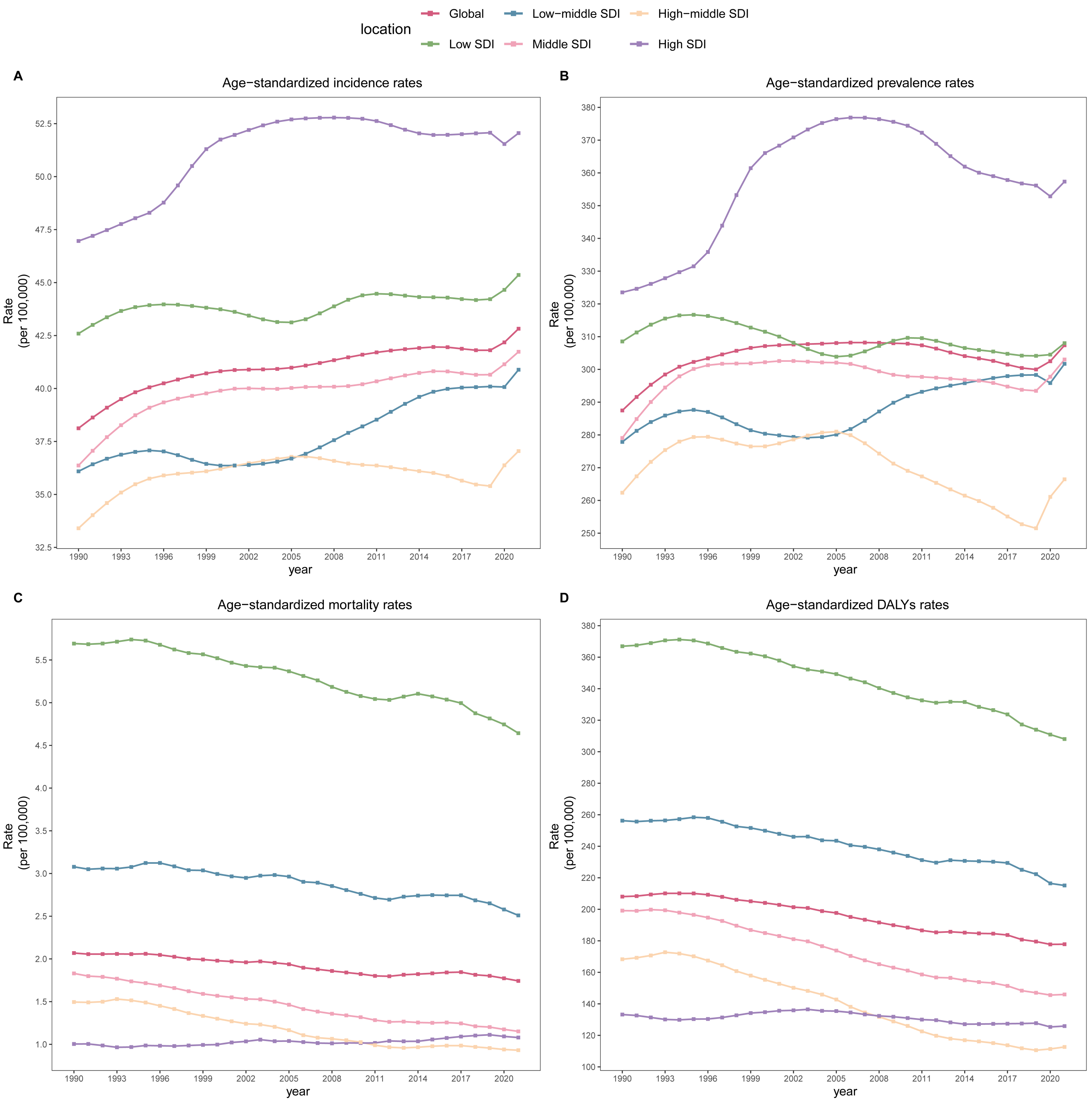
location
Global
Low−middle SDI
High−middle SDI
Low SDI
Middle SDI
High SDI
A
Age−standardized incidence rates
B
Age−standardized prevalence rates
C
Age−standardized mortality rates
D
Age−standardized DALYs rates
Rate
(per 100,000)
year

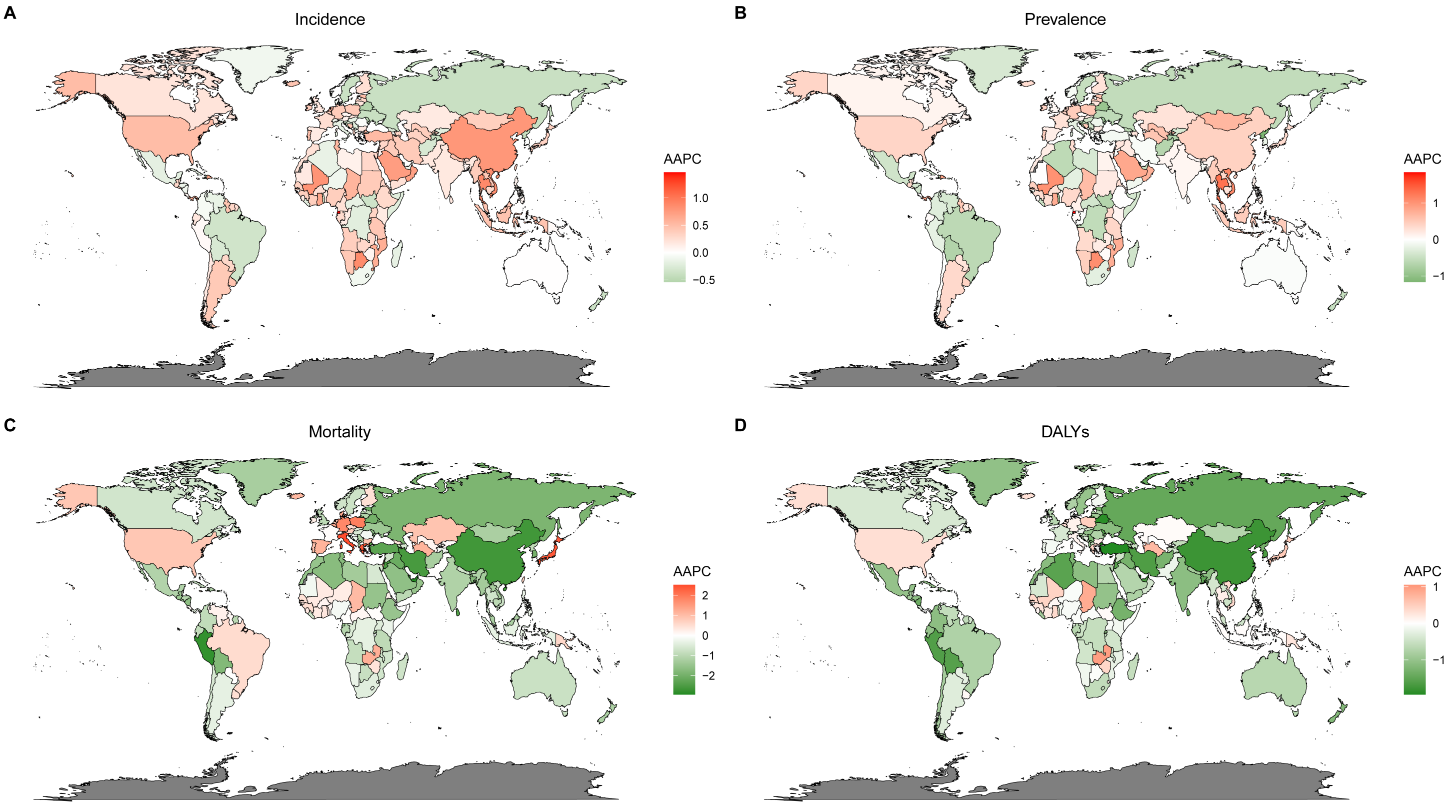
A
Incidence
AAPC
1.0
0.5
0.0
−0.5
B
Prevalence
AAPC
1
0
−1
C
Mortality
AAPC
2
1
0
−1
−2
D
DALYs
AAPC
1
0
−1

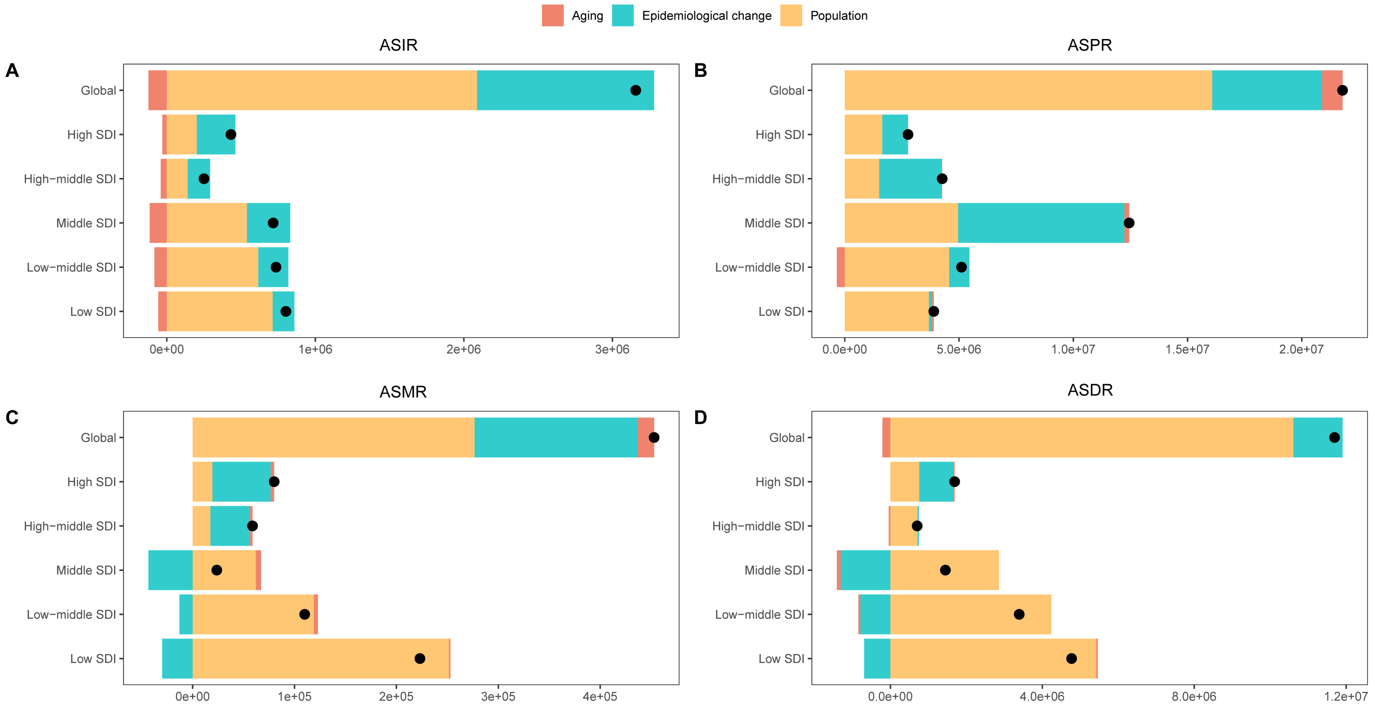
Aging
Epidemiological change
Population
A
ASIR
Global
High SDI
High−middle SDI
Middle SDI
Low−middle SDI
Low SDI
0e+00
1e+06
2e+06
3e+06
B
ASPR
0.0e+00
5.0e+06
1.0e+07
1.5e+07
2.0e+07
C
ASMR
0e+00
1e+05
2e+05
3e+05
4e+05
D
ASDR
0.0e+00
4.0e+06
8.0e+06
1.2e+07

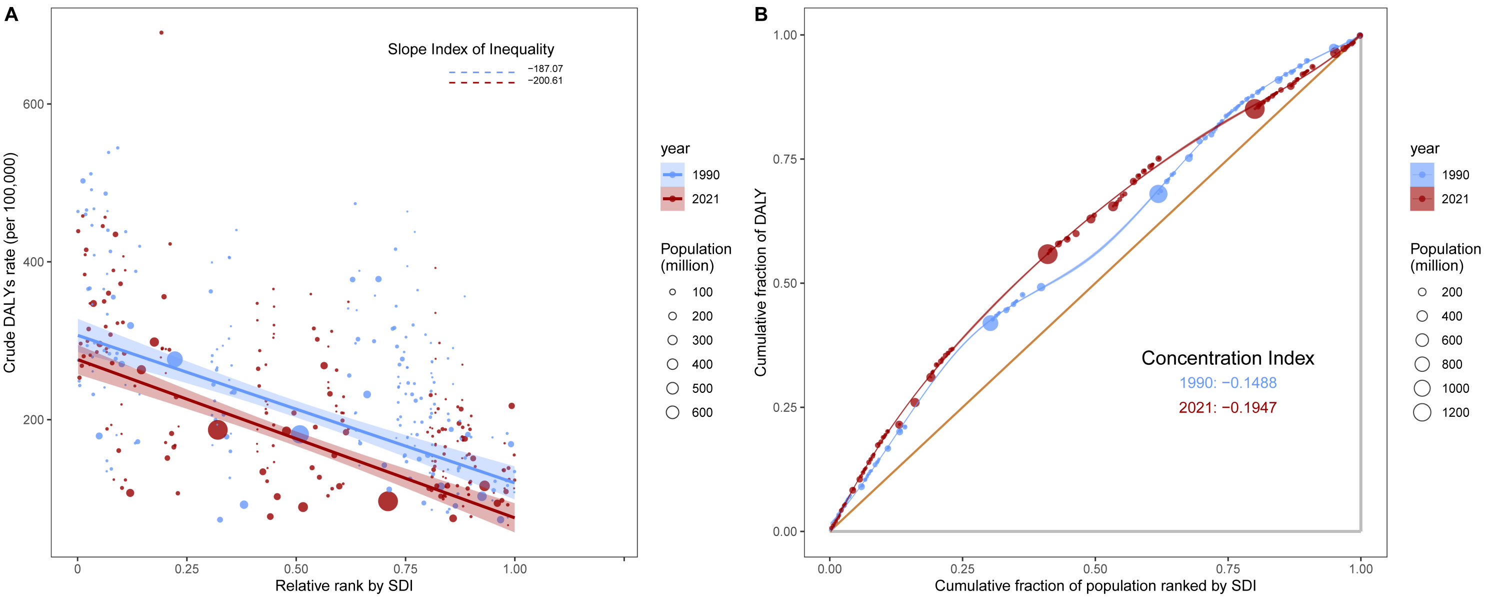
A
Slope Index of Inequality
−187.07
−200.61
Crude DALYs rate (per 100,000)
Relative rank by SDI
year
1990
2021
Population (million)
100
200
300
400
500
600
B
Concentration Index
1990: −0.1488
2021: −0.1947
Cumulative fraction of DALY
Cumulative fraction of population ranked by SDI
year
1990
2021
Population (million)
200
400
600
800
1000
1200

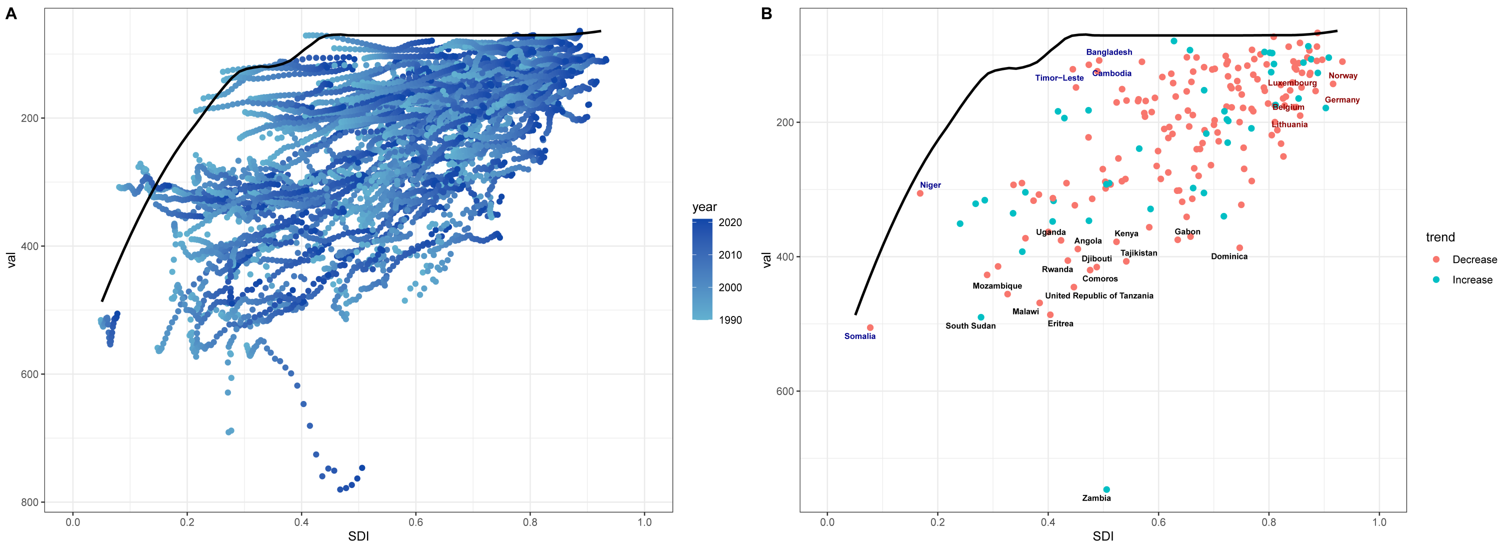
A
val
SDI
year
2020
2010
2000
1990
B
Bangladesh
Timor−Leste
Cambodia
Norway
Luxembourg
Germany
Belgium
Lithuania
Niger
Uganda
Angola
Kenya
Gabon
Djibouti
Tajikistan
Dominica
Rwanda
Comoros
Mozambique
United Republic of Tanzania
Malawi
South Sudan
Eritrea
Somalia
Zambia
val
SDI
trend
Decrease
Increase

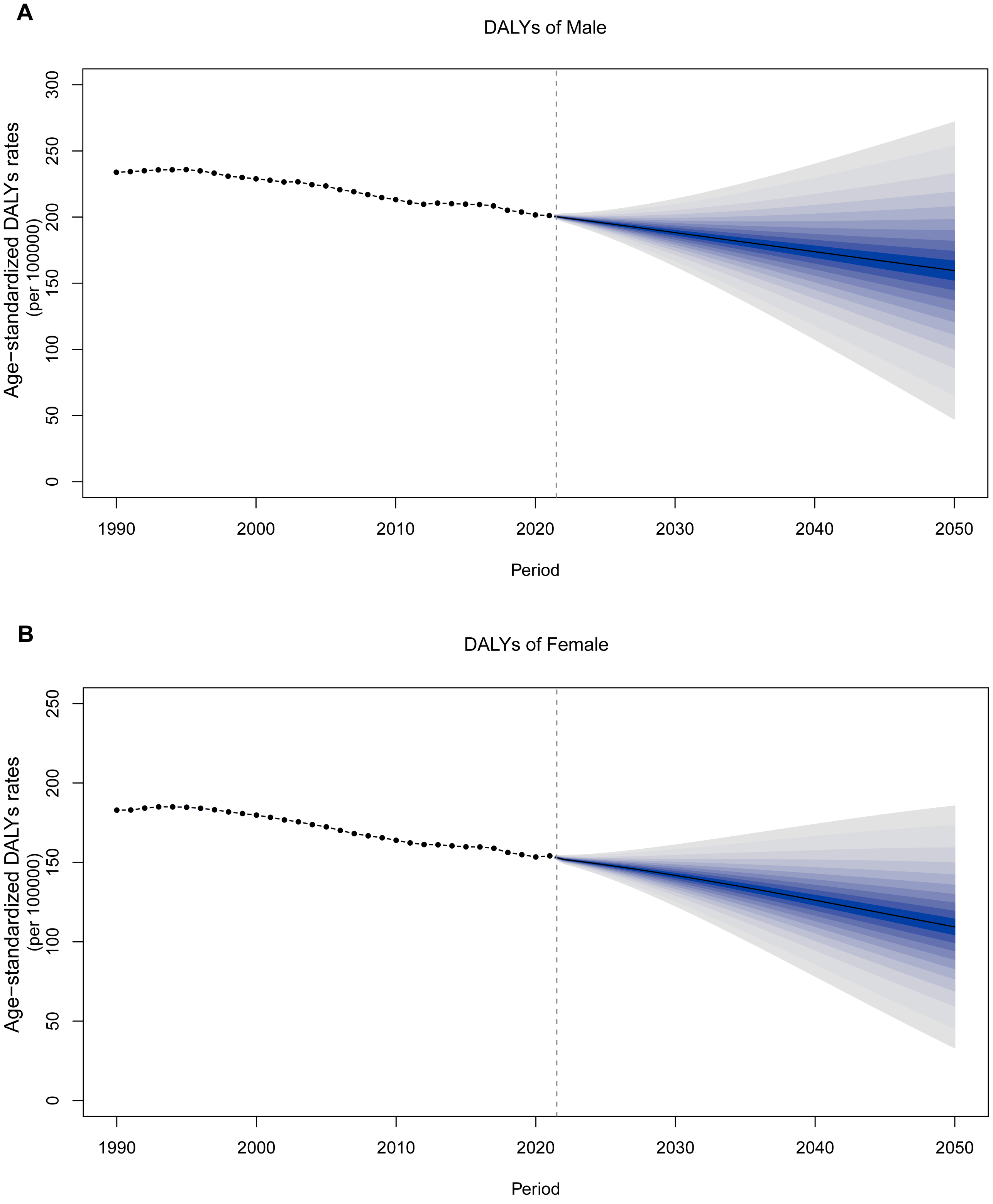
A
DALYs of Male
Age−standardized DALYs rates
(per 100000)
0
50
100
150
200
250
300
1990
2000
2010
2020
2030
2040
2050
Period
B
DALYs of Female
Age−standardized DALYs rates
(per 100000)
0
50
100
150
200
250
1990
2000
2010
2020
2030
2040
2050
Period

Table S1

Germany	56904.9 (18186.6-89582.6)	79.9 (25.6-128.1)
Iraq	8660.3 (2251-16628.5)	39.2 (10-73.7)
Israel	2362.2 (641.6-3928.1)	46.8 (12.7-77.6)
Ireland	2019.6 (526.3-3391.2)	56.9 (14.9-95)
Myanmar	14834.7 (2995.5-29537.6)	33.2 (6.7-64.8)
Uzbekistan	10427 (2763.3-18458.9)	43 (11.6-75.9)
Nigeria	61395.6 (38848.4-86523.9)	59.4 (37.3-83.2)
Ivory Coast	7629.9 (1397.5-14991.6)	54 (9.8-104.3)
Angola	7409.3 (1398.1-15069.8)	62.1 (11.5-122.8)
Guinea	3419.6 (630.7-7099.8)	47.8 (8.7-99.1)
Mongolia	962.9 (199.9-1699.2)	38.2 (8-65.6)
El Salvador	2935.8 (710.4-5472)	49.4 (12.6-90.6)
Belgium	5515.4 (1645.4-8807.2)	59.8 (17.6-97.9)
Taiwan (Province of China)	6173.9 (1951.6-10167.5)	31.3 (9.9-51.5)
Japan	45063.9 (30216.6-60664.5)	39.5 (26.1-54)
Senegal	4734.9 (687.5-9207.7)	50.6 (7.4-97.5)
Turkmenistan	1831.2 (513-3278.1)	42.4 (12.4-74.6)
Uganda	12496.3 (1881.8-25762.9)	58.1 (9.1-119.1)
Seychelles	29 (7.8-51.5)	38 (10.3-67.1)
Belize	107 (23.5-195.7)	50.6 (11.3-90.6)
Albania	1403.3 (388.6-2538.6)	39.4 (11-70.7)
Eritrea	2027.8 (362.9-4176.2)	51.1 (8.8-103.3)
Saint Vincent and the Grenadines	68.6 (15.4-125.2)	57.9 (13.1-104.1)
Azerbaijan	3368.3 (791.2-5905.2)	42.8 (10.1-73.8)
Guatemala	5390.8 (1152.2-10537.1)	55.7 (12-108.2)
Suriname	247.4 (55.4-448.6)	60.4 (13.5-108.3)
Syrian Arab Republic	5390.6 (1160.6-10414)	34.7 (7.5-64.5)
Paraguay	2129.9 (531.3-3852.9)	47.7 (11.6-86.7)
Latvia	917.9 (219.6-1541.6)	37.1 (8.8-63.6)
Cambodia	3530.3 (711.3-6958)	29.2 (6.1-57.3)
Zambia	6270.6 (1263.2-12060.2)	66.4 (13.5-124.2)
Egypt	25862.2 (5490.4-48897.3)	40.6 (8.6-75.9)
Bhutan	202.7 (32.8-421)	28.9 (4.8-58.8)
Ethiopia	21706.9 (8275-36676.7)	37.6 (14.4-62.8)
Djibouti	264.3 (50.7-539.9)	56.4 (10.7-113.8)
Gabon	816 (164.4-1453.7)	75.6 (15.5-134.7)
France	38644.6 (12146.3-61224.6)	70 (22-111.5)
Palestine	1020.4 (200.2-1966.3)	40.8 (7.9-78.2)
Grenada	54.1 (14.5-94.5)	58.5 (15.6-101)
Burkina Faso	5423.9 (912.2-11932.7)	46.9 (7.8-102.7)
Finland	2282 (750.4-3762.6)	48.3 (15.6-78)
Dominica	55 (12.6-95.6)	71.8 (16.5-124.2)
Yemen	5317 (888-10890)	30.9 (5.1-63.5)

Lebanon	1359.6 (338.3-2444)	42.3 (10.7-75.3)
Luxembourg	232.8 (72.9-365.5)	65.4 (20.8-105.3)
Cameroon	6522.8 (1198.2-12816.8)	53.4 (10.1-103.6)
Morocco	11271.2 (2725.1-20911.3)	39.3 (9.4-72.2)
Niue	0.9 (0.2-1.5)	37.2 (7.5-64.7)
Fiji	312.3 (87.3-553)	38.3 (10.8-66.9)
Canada	8780.3 (2731.5-14179.8)	33.4 (10.4-53.8)
Monaco	14.8 (4.3-23.3)	57 (16.6-91)
Tonga	33.7 (8-59)	30.8 (7.3-53.2)
Slovakia	2190 (605.3-3589)	43.1 (11.9-71.3)
Lithuania	1415.9 (429.1-2345.1)	40.5 (12.2-67)
Trinidad and Tobago	876.4 (242.9-1459.2)	69.8 (19.5-115.5)
Ecuador	9356.5 (2127.7-16557.8)	88.4 (20.2-156.4)
Pakistan	42928.5 (17472.1-73791.3)	34.2 (14.4-57.9)
Samoa	62.7 (13.1-113.8)	33.5 (7-59.4)
India	280473.1 (172090.2-405203.3)	31.1 (19.4-43.6)
Bulgaria	3195 (1014.4-5378.1)	41.8 (13.4-70.8)
United Kingdom	36201.1 (25979.9-48032.2)	68.2 (48.5-89.3)
Sri Lanka	8830.5 (1913.7-15483.7)	50 (10.9-87.7)
Chile	8754.1 (2624.2-14471.2)	63.1 (18.9-103.7)
Mauritania	1253.6 (289.8-2373.5)	53.2 (12.1-100.3)
Solomon Islands	112 (22.4-222.8)	29.2 (5.8-56.9)
Kazakhstan	7952.1 (2109.2-13507.5)	46.5 (12.4-78.9)
Benin	3107.9 (499.3-6243.3)	51.8 (8.6-104.1)
Kenya	17292.7 (11071.8-24125.6)	62.9 (40.9-85.6)
Spain	14898 (3895.4-25410)	41.7 (11.1-70.5)
Congo	1749.5 (349.6-3470.9)	64.5 (13.1-124.8)
Sierra Leone	2352.6 (388.8-4559.5)	47.7 (7.9-90.6)
Comoros	290.7 (51.1-583.7)	54.7 (9.5-109.9)
Tokelau	0.5 (0.1-1)	31.3 (7.4-57.9)
Maldives	106.5 (20.4-208.9)	41.5 (7.7-80)
Vanuatu	50.7 (11.3-95.2)	29.6 (6.4-55.6)
American Samoa	20.2 (5.8-35.9)	38.6 (11.2-68.7)
Marshall Islands	16.2 (3.7-30.2)	31.7 (7.3-56.8)
Austria	3922 (1176.5-6375.1)	55.5 (16.7-91)
Libya	2061.1 (439.5-3833)	42 (8.9-75.7)
Cook Islands	7.8 (2.2-14.1)	39.5 (11.5-70.3)
Guam	54 (13.9-91)	38.6 (9.9-63.6)
Jordan	1892.9 (510.3-3320.1)	42.1 (11.3-72.8)
Philippines	24826.5 (16964-33441.9)	35 (24.3-46.7)
Zimbabwe	5434.9 (1219-10086.6)	48.5 (10.6-88.9)
Czechia	4205 (1340.5-6891.7)	45 (14.4-73.5)
Palau	6.6 (1.5-11.5)	41.9 (9.7-72.9)

Jamaica	1471.6 (411.1-2543.5)	58.4 (16.6-98.3)
Tuvalu	3.3 (0.9-5.8)	32.1 (8.9-57.4)
Bahrain	296.8 (83.1-509)	57.5 (16.4-98)
Costa Rica	2041.9 (579.7-3600.5)	62.6 (18.2-107.9)
Bangladesh	31788.7 (6703.7-60824)	25.1 (5.2-47.7)
Peru	15724.8 (3578.3-27518.6)	66.8 (15.7-115.2)
Antigua and Barbuda	48.5 (12.3-80)	78 (19.9-128.2)
Papua New Guinea	1266.5 (269.8-2522.1)	27.6 (5.9-54.6)
Greece	4415 (1362.5-7134.6)	47.1 (14.6-78.3)
Malawi	6100.3 (1248-12173.3)	51.8 (10.8-103.2)
Guinea-Bissau	649.1 (97.7-1304.5)	54.4 (8-109.7)
Australia	7813.4 (2417.1-12611.7)	48.2 (14.7-77.1)
Norway	2686.5 (1660.2-3791.3)	67.4 (41.9-96.4)
United States Virgin Islands	71.6 (19.9-117.5)	66.4 (18.5-108.4)
Republic of Korea	21236.7 (5864.4-34676.7)	47.7 (13.3-77.1)
Saint Lucia	99.1 (26-175.2)	67.5 (18.4-119.4)
Romania	9246.1 (2581.3-15622.8)	42.3 (11.6-71.2)
Kuwait	987.8 (236-1674.1)	54.2 (13.1-91)
San Marino	11 (3-17.7)	50.8 (14.2-82)
Montenegro	235.7 (70.9-395.5)	38.8 (11.6-65.3)
Micronesia (Federated States of)	39.5 (9.6-73.8)	34.4 (8.2-62.1)
Armenia	1326.2 (358.1-2323.2)	37.5 (10.1-65.7)
Colombia	23385.5 (5469.3-40539.8)	65.8 (15.6-114)
Cyprus	394.8 (101.6-668)	51.9 (13.4-87.2)
North Macedonia	776.8 (238-1332.2)	39.8 (12.2-68.1)
South Africa	23832.4 (14673.3-34918.6)	59.5 (37.4-84.9)
United Arab Emirates	1284.2 (383.7-2167.9)	66.4 (19.9-111.6)
Denmark	2028.3 (598.1-3286.5)	43.9 (13.2-71.1)
Greenland	30.8 (8.3-51.8)	55.4 (14.7-92.3)
Switzerland	3618.1 (1159.6-5688.8)	55.6 (17.8-88.7)
Niger	4259.5 (541.9-9308.8)	42.1 (5.5-92.6)
Rwanda	4852.3 (933.3-9466.5)	56.8 (10.9-109.3)
Nicaragua	2381.6 (539.9-4383.7)	53.3 (12-96.2)
Honduras	3190.4 (603-6323)	58.8 (11.6-113.7)
Bahamas	183.6 (52-310.2)	67.9 (19.3-113.6)
Kiribati	30.8 (7.4-56.1)	37.6 (9-67.1)
Venezuela (Bolivarian Republic of)	13862.6 (3173.9-25480)	67.9 (15.5-123.3)
Algeria	13814.8 (3609.4-25652.3)	46.7 (12.6-84.7)
Hungary	4000.4 (1112.3-6781.6)	43.4 (11.7-74.5)
Cuba	5031.9 (1411.3-8733.5)	46.5 (12.9-79.5)
Madagascar	7703.6 (1505.8-15442.8)	54.7 (10.6-108.5)
Italy	22699.7 (14656.7-30977.7)	44.5 (28.3-60.7)
Central African Republic	1575.5 (318.8-3208.9)	50.2 (9.9-100.5)

Andorra	31.8 (10.7-49.1)	61.8 (21.4-95.9)
Mexico	76377.2 (53506.4-104458.6)	81.3 (57.3-111.2)
Liberia	1607.7 (301.2-3292.6)	54.7 (10.2-110.5)
Kyrgyzstan	2350.4 (578.1-4222.5)	47.3 (11.6-83.4)
Democratic Republic of the Congo	23430.2 (4034.6-46523.7)	52.1 (8.8-100.8)
Barbados	168.9 (48.8-279.7)	67.3 (19.5-112)
Somalia	3605.5 (504.2-8021.2)	38.2 (5.5-84)
Qatar	276.5 (74.3-471.2)	59.4 (15.5-100.2)
Serbia	4292.7 (1181.8-7242)	48.1 (13.4-81.1)
Puerto Rico	2429.8 (719.6-4153.7)	67.4 (20.1-115.6)
Georgia	2320.6 (559.4-3895)	43.7 (10.7-74.1)
Haiti	3656.2 (688.2-7025.8)	51 (9.3-97.9)
Northern Mariana Islands	18.6 (4.3-31.7)	41.2 (9.7-69.1)
Saint Kitts and Nevis	33.9 (8.9-58.4)	79.1 (20.8-134.7)
Nauru	4.7 (1-8.5)	42 (9.4-74.9)
Croatia	2225.9 (530-3623.9)	49.3 (11.9-81.4)
Bolivia (Plurinational State of)	3780.6 (832.1-6893.1)	53.9 (11.7-97.8)
Afghanistan	5516.1 (921.1-11013)	48 (8.3-96.1)
Bermuda	39.3 (10.6-63.9)	69.5 (17.8-112.6)
Slovenia	852.8 (281.2-1381.8)	46.4 (15.5-74.6)
Brazil	97572.1 (60874.8-135800.7)	61.4 (38.7-84.5)
South Sudan	3825.5 (839.2-7669.9)	56.4 (12.5-112.7)
Togo	2888.4 (587.2-5710.3)	63.9 (13-125.1)
New Zealand	1892.5 (747.7-2902.1)	57.1 (22.4-87.8)
Sweden	3465.4 (1343.1-5488)	44.9 (17.5-71.8)
Russian Federation	51039.4 (36088.3-68916.2)	35.9 (25.2-48.7)
Tajikistan	3181.3 (777-5940.1)	50 (12.4-92.6)
Ukraine	19592.9 (6536-31650.7)	41.1 (13.8-66.4)
Brunei Darussalam	174.6 (48.2-304)	63.7 (17.5-108.8)
Democratic People's Republic of Korea	5222.9 (1155-9319.1)	25 (5.6-45)
Republic of Moldova	1633.6 (436.9-2813.2)	37.1 (10-63.8)
Burundi	3609.8 (566.5-7126.8)	55 (8.6-107.9)
Belarus	3624.2 (994.5-6367)	37.1 (10.2-65)

Num_2021	ASR_2021	AAPC	
1297 (393.9-2171.9)	84.9 (20.6-140.9)	1.47	1.336 - 1.597
349.9 (81.5-615.9)	66.9 (18-115.6)	0.92	0.873 - 0.974
940.1 (227.3-1688.8)	49.1 (10.3-86.5)	0.91	0.853 - 0.956
24954.8 (5844.5-41180.9)	43 (13.9-71.2)	0.88	0.85 - 0.908
1427.5 (367.4-2533.7)	63.4 (18.2-106.3)	0.86	0.789 - 0.938
33499.6 (8380.3-56422)	34.6 (8-58.9)	0.84	0.632 - 1.044
11194.6 (2061.7-21263.3)	43.6 (10.2-84.1)	0.8	0.422 - 1.184
321104.6 (228734.5-429680.5)	28.2 (19-37.9)	0.78	0.65 - 0.911
23399.9 (6652.9-39457.9)	67.1 (17.6-107.4)	0.74	0.7 - 0.775
131.6 (33.2-234.8)	61.3 (17.8-104.1)	0.73	0.71 - 0.75
2090.2 (654.7-3426.3)	49.9 (14.8-79.5)	0.72	0.654 - 0.781
19478.5 (5249-33589.5)	56.4 (15.1-95.6)	0.66	0.601 - 0.724
6138.5 (1688.4-10559.3)	58 (16.4-98.5)	0.66	0.615 - 0.706
667.4 (194.4-1200)	56.6 (13.6-98.5)	0.64	0.558 - 0.72
2926.1 (842.3-4801.5)	70.8 (19.9-114)	0.62	0.604 - 0.632
18423.5 (4409.6-36979.4)	57 (11.1-104.6)	0.61	0.425 - 0.797
709.8 (217.7-1166)	61.4 (21.2-99.5)	0.61	0.576 - 0.633
9715.1 (1750-18803.3)	49.3 (8.7-97.3)	0.56	0.505 - 0.621
8791 (3305.7-13373.6)	56.4 (16.1-91.4)	0.56	0.477 - 0.646
478.5 (107.8-884)	34.2 (9.7-60.3)	0.55	0.323 - 0.769
4473 (1204.6-7472.3)	42.1 (12.1-71.3)	0.54	0.51 - 0.568
44699 (15484.7-70581.1)	57 (17.8-93.3)	0.52	0.411 - 0.626
129359.5 (85765-175485.6)	42.9 (27.1-60.7)	0.5	0.446 - 0.563
525.5 (160.5-850.9)	44.9 (13.7-69.5)	0.49	0.364 - 0.622
4591.7 (1403.9-7312.8)	47.7 (12.7-76.4)	0.48	0.364 - 0.592
1227.1 (296-2233)	51 (11.7-90.3)	0.46	0.424 - 0.489
88007.4 (60172.6-118851.4)	34.9 (22.9-46.8)	0.45	0.439 - 0.468
1841.6 (486.1-3029.9)	56.3 (13.4-91.5)	0.45	0.396 - 0.511
166.6 (41.8-264.3)	51.4 (14.7-80.3)	0.44	0.407 - 0.475
472.5 (124.5-814.8)	63.8 (18.5-104.9)	0.44	0.403 - 0.473
10474.5 (2130.6-19120.5)	35.8 (7.8-63.6)	0.44	0.339 - 0.536
12946.6 (3191.3-21895.5)	44.6 (11.8-72.7)	0.44	0.411 - 0.458
1159.5 (365.8-1907.7)	41 (11.2-69.1)	0.43	0.401 - 0.458
36343.8 (9527.3-65475.4)	60.7 (14.6-108.5)	0.43	0.118 - 0.733
195.1 (55.3-304.5)	51.4 (15.2-84.2)	0.42	0.306 - 0.536
2362.9 (536.5-4380.9)	33.4 (8.5-56.8)	0.42	0.39 - 0.45
1313.9 (363.4-2204.7)	53.4 (15.7-92.2)	0.42	0.403 - 0.431
19157.6 (6389.7-31604.9)	45.6 (11.4-77.2)	0.41	0.349 - 0.469
2098.4 (562.5-3342.5)	43 (12.2-69.1)	0.41	0.393 - 0.421
12395.2 (8129.6-16822.6)	39.1 (26.1-53.7)	0.41	0.344 - 0.466
38567.1 (25731.7-51861.3)	50 (33.8-65.7)	0.4	0.35 - 0.453
18213.9 (5316.7-31843.2)	39.1 (10.3-67.1)	0.4	0.328 - 0.47

79051 (29341.8-116708.2)	91.8 (27.5-140.5)	0.39	0.168 - 0.614
18508.2 (5406.8-30274.1)	44.2 (13.3-73.4)	0.39	0.34 - 0.438
4751.9 (1224.4-7515.8)	52.9 (17.4-83.2)	0.39	0.359 - 0.417
2795.1 (828.2-4427.2)	63.8 (19.4-100.7)	0.39	0.22 - 0.554
20597.8 (5304.7-36113.4)	37.5 (10.5-66.7)	0.38	0.315 - 0.453
16127.6 (4464.8-26829.8)	49.4 (13.6-79.9)	0.38	0.154 - 0.603
159455.9 (116918.8-209924.6)	66.4 (47.6-86.1)	0.37	0.297 - 0.446
16782.3 (3680.2-30114.7)	60.7 (14.5-109.1)	0.37	0.314 - 0.423
23805.2 (5927.7-42159.6)	69.3 (18.7-119.9)	0.37	0.328 - 0.406
7301.7 (1562.5-13566.1)	52.7 (11.5-103.7)	0.37	0.176 - 0.558
1437.1 (424.4-2541.1)	43.2 (9.2-74)	0.36	0.23 - 0.494
3573.4 (694.3-6134.6)	55 (13.2-92.6)	0.35	0.241 - 0.466
7320.8 (2368.8-11453.7)	66.7 (19.5-102.2)	0.35	0.281 - 0.414
7684.5 (2550-11696.5)	35 (9.3-55.4)	0.34	0.232 - 0.455
46847.5 (31222.7-62810.8)	44 (28.3-61)	0.34	0.297 - 0.387
9391.7 (2490.3-17219.4)	56.3 (12-104.7)	0.34	0.257 - 0.421
2558.9 (624.6-4272.2)	47.5 (11.3-81.9)	0.34	0.286 - 0.389
29187.1 (5977.7-53899.9)	64.1 (13.4-115.2)	0.32	0.233 - 0.411
41.4 (9.7-69.5)	42 (12.3-72)	0.32	0.181 - 0.463
235 (75-410.7)	55.8 (15.7-97.3)	0.32	0.238 - 0.394
1004.7 (271.1-1663.7)	43.6 (11.1-70.7)	0.31	0.165 - 0.455
3725.7 (864-6681.8)	55.8 (13.9-102.5)	0.31	0.145 - 0.475
67.1 (17.9-113.3)	63.9 (15.7-104.4)	0.31	0.284 - 0.334
4760.2 (1349.6-7745.4)	47.1 (14-76.8)	0.31	0.238 - 0.38
10063.1 (2752.5-17148.3)	61.5 (17-101.7)	0.31	0.28 - 0.337
363.3 (102.6-591.2)	66.6 (21.6-107.7)	0.31	0.289 - 0.326
5025.5 (1568.1-8675.7)	38 (10.6-62.4)	0.31	0.113 - 0.5
3793.7 (860.4-6263.2)	52.4 (17.3-86.1)	0.3	0.29 - 0.316
667.1 (170.4-1052.1)	40.9 (12.4-65.2)	0.3	0.229 - 0.373
5491.7 (1374.1-10125.6)	32.1 (10-58.7)	0.3	0.252 - 0.348
14647.6 (3838.8-25534.6)	73.1 (18.7-129)	0.3	0.238 - 0.356
49219 (16723.6-78318)	46.3 (12-77.7)	0.3	0.065 - 0.527
233.4 (60.1-437.5)	31.8 (8.5-57.8)	0.29	0.231 - 0.351
46273.1 (24186.2-70085.7)	41.4 (21.9-62.3)	0.29	0.163 - 0.41
736.5 (157.7-1323)	61.9 (16.5-110.3)	0.28	0.263 - 0.296
1538.3 (399.4-2634.4)	82.8 (17.7-140.9)	0.28	0.207 - 0.344
48262.8 (16567.8-72294.1)	76.2 (26.3-120.9)	0.25	0.207 - 0.301
2402 (766.3-4005.9)	44.2 (13.9-77.6)	0.25	0.199 - 0.308
61.8 (16.8-101)	63.1 (17.4-104.4)	0.25	0.237 - 0.258
12692.7 (2693.1-25575.6)	50.8 (9.2-99)	0.25	0.19 - 0.303
2539.1 (847.6-3937.7)	52.4 (15.7-84.1)	0.24	0.18 - 0.309
48.6 (12-77.9)	77.6 (27.2-123.4)	0.24	0.176 - 0.301
11964.2 (2937.4-21763.9)	33.3 (6.5-61.4)	0.23	0.186 - 0.277

2382.3 (658.5-3921.2)	45.6 (14.3-76.6)	0.23	0.197 - 0.261
404.9 (116.9-627.7)	70.2 (23.9-109.7)	0.23	0.142 - 0.315
19168.1 (4114.3-35290.4)	57.8 (14.9-102.3)	0.23	0.034 - 0.424
15091.5 (3668.9-25938.2)	43.2 (10.5-74.4)	0.22	0.109 - 0.34
0.6 (0.2-1)	39.8 (11.9-64.7)	0.22	0.186 - 0.25
376.1 (106.3-636.2)	41.2 (12.1-66.5)	0.22	0.116 - 0.32
12068.5 (3635.7-18756.5)	35.3 (9-57.5)	0.21	0.023 - 0.398
19.7 (5.8-30.1)	60 (18.9-96.1)	0.21	0.098 - 0.317
36.4 (8.9-63.2)	32.9 (10.2-52.8)	0.2	0.181 - 0.214
2146.5 (618.4-3543.2)	46 (13.5-76.6)	0.2	0.149 - 0.246
1011 (266.5-1634.3)	42.8 (13.8-67.3)	0.19	0.116 - 0.261
945.2 (237.1-1518)	72.3 (20.5-119)	0.19	0.06 - 0.319
16764.4 (5336.6-28323.3)	94.9 (29.9-160.5)	0.18	-0.087-0.457
84530.7 (44582-129195.3)	36.1 (18-54)	0.18	0.149 - 0.208
74.6 (20.3-130.8)	35.2 (11-59.4)	0.18	0.159 - 0.198
457885.5 (323771-614105.9)	33.2 (23.6-43.2)	0.17	0.001 - 0.345
2411.6 (790.6-3835.1)	43.5 (12-71.1)	0.17	0.052 - 0.29
42107.3 (28195.3-55897.8)	72 (49-97.8)	0.17	0.143 - 0.197
11186.2 (3396.5-18559.9)	52.8 (12.8-86.9)	0.17	0.137 - 0.196
11279.6 (2723.6-19271.2)	66.3 (19.9-107.2)	0.17	0.112 - 0.221
2503.3 (661.8-4367.8)	55.8 (15.7-95.1)	0.17	0.096 - 0.236
222.7 (46-401.4)	30.4 (7-55)	0.17	0.078 - 0.252
9119 (2782.9-15057)	49.8 (14.1-82)	0.16	-0.024-0.347
7861.7 (1841.6-14601.4)	55 (13-100.6)	0.16	0 - 0.321
32777.9 (23254.6-43788.5)	66.2 (48.5-87)	0.16	0.068 - 0.245
17792.2 (4993.1-28287.4)	44 (13.7-71.8)	0.16	0.007 - 0.305
3702.1 (798.5-6425.5)	67.6 (18.9-115)	0.15	0.117 - 0.181
4517.9 (1037.2-8484.2)	49.8 (10-92.5)	0.15	0.06 - 0.238
424.3 (108.5-743.6)	57.1 (17.1-101)	0.14	0.075 - 0.212
0.4 (0.1-0.7)	33.1 (7.7-59.4)	0.14	0.01 - 0.268
184.6 (47.5-316.6)	43.6 (13-75.8)	0.14	0.058 - 0.214
100.2 (22.3-178.8)	30.8 (7-53.4)	0.13	0.088 - 0.175
21 (6.4-34.6)	40.3 (10.5-65.5)	0.13	-0.131-0.389
19.3 (5.4-34.6)	32.8 (8.3-60.2)	0.13	0.096 - 0.154
4256.2 (1328.6-6709.4)	57.4 (16.7-90.4)	0.11	0.069 - 0.157
2789.2 (856-4635.4)	43.5 (12.8-69.9)	0.11	0.061 - 0.161
6.9 (2-11.2)	41 (12.9-66.5)	0.11	0.017 - 0.202
62.9 (18.3-99)	39.9 (13.6-65.1)	0.11	0.071 - 0.141
5101.1 (1663.9-8352.6)	43.5 (12.3-70)	0.1	0.029 - 0.176
40403.5 (27982.1-54358.6)	36.4 (25.5-48.6)	0.09	0.048 - 0.123
7941.8 (1796.1-13971.4)	50.2 (11.8-91.6)	0.08	0.005 - 0.162
4228 (1282.4-6403.5)	46.1 (13.6-75.7)	0.08	0.064 - 0.097
7.1 (2.1-11.3)	43 (11.8-69.7)	0.08	0.02 - 0.142

1600.6 (436.1-2688.1)	58.5 (17-95.4)	0.08	-0.057-0.211
4 (1.1-6.8)	33.2 (9.5-56.6)	0.07	-0.001-0.145
783.9 (197.4-1339.3)	59.1 (17.2-97.8)	0.07	0.006 - 0.134
2846.3 (814.1-4770.1)	63.5 (18.8-104.7)	0.07	-0.025-0.161
39191.8 (9623.1-72207.5)	25.7 (7.4-45.5)	0.07	0.017 - 0.115
22491.8 (6773.2-37934.9)	67.8 (18.9-113)	0.04	-0.02-0.107
67.2 (19.5-109)	79.1 (21.1-126.7)	0.04	-0.012-0.094
3008 (755.3-5624.7)	27.9 (6.6-50.8)	0.04	-0.026-0.102
4046.9 (1300.1-6296.3)	47.9 (12.4-76.1)	0.03	-0.039-0.106
10798.3 (2422.7-20330.9)	52.1 (12.3-94.2)	0.02	-0.024-0.073
1159.2 (281.6-2169.5)	54.3 (10.4-100.7)	0.02	-0.125-0.168
10917.6 (3173.9-16988.8)	48.3 (13.7-79.7)	0.01	-0.038-0.05
3293.3 (1903.7-4515.1)	67.4 (40.5-93.2)	0	-0.042-0.04
52.7 (14.6-82.5)	66.4 (20.2-107.6)	-0.01	-0.123-0.101
21283.7 (6166.2-33175.5)	47.7 (12.1-74.3)	-0.01	-0.052-0.026
110.1 (29-180.6)	65.7 (17.1-111.3)	-0.03	-0.098-0.045
6654.5 (2122.9-10912.5)	42.1 (12.6-68.1)	-0.03	-0.14-0.081
2072.5 (628.2-3381)	53.9 (14.7-86.6)	-0.03	-0.091-0.028
13.3 (3.8-20.2)	50.9 (14.5-78.5)	-0.04	-0.149-0.074
201.3 (52.6-341.3)	38.2 (10.4-62.6)	-0.04	-0.104-0.019
35.3 (10-60.3)	34.1 (7.2-58.6)	-0.06	-0.093--0.018
981.3 (227.4-1638.2)	38 (12.3-64)	-0.07	-0.233-0.103
29692.6 (8155.1-51978)	64.4 (16.2-110.3)	-0.07	-0.083--0.052
599.4 (181.6-917.1)	51.1 (14-82.3)	-0.08	-0.177-0.028
715.8 (217.7-1209.7)	38.7 (11.3-62.8)	-0.08	-0.099--0.069
32137.9 (19941.1-45502.2)	58.7 (35.8-81.5)	-0.09	-0.311-0.128
5381.2 (1629.6-8896.5)	64.4 (22-105.6)	-0.09	-0.166--0.017
2227.9 (669.2-3485.9)	42.6 (11.1-66.9)	-0.09	-0.187--0.001
29.6 (8.7-48.1)	53.8 (17.9-86.2)	-0.1	-0.153--0.054
4385.7 (1253.3-6684.3)	54.1 (14.9-85.3)	-0.1	-0.139--0.066
11182.6 (1988.7-23278.7)	41 (7.1-85.1)	-0.11	-0.147--0.073
7297.5 (1830.8-13220.3)	54.7 (11.9-98.7)	-0.11	-0.165--0.058
3462.7 (970.2-5935.9)	51.3 (12.6-95.6)	-0.11	-0.128--0.094
5774.6 (1388.5-10720.2)	56.6 (12.4-97.7)	-0.13	-0.167--0.099
244.6 (65.5-391.1)	65.5 (20.6-105)	-0.14	-0.202--0.078
44.4 (11-77.5)	35.6 (8.8-63.9)	-0.15	-0.212--0.096
18493.2 (4983.3-31092.6)	64.6 (20-110.9)	-0.16	-0.216--0.098
19295.6 (5547.9-32371.9)	43.9 (11.8-74.7)	-0.16	-0.216--0.102
3199.2 (1139.4-4999.8)	41.1 (12.8-68)	-0.16	-0.213--0.116
4029.5 (991-6800.7)	44 (12.2-75.4)	-0.17	-0.212--0.124
15266 (3813-27101.8)	52.1 (13.4-96.6)	-0.17	-0.19--0.152
22914.7 (15504.2-31101.8)	43.1 (28.7-59.7)	-0.17	-0.369-0.022
2895.3 (546.4-5552.1)	47.9 (9.9-89.4)	-0.18	-0.37-0.02

42 (9.7-65.1)	59.6 (17.2-92.1)	-0.18	-0.26--0.091
97370.6 (68247.8-132231.8)	77 (52.8-101.6)	-0.18	-0.206--0.161
2940.6 (589.1-5530)	52 (10.5-97.4)	-0.19	-0.273--0.106
3112.8 (1028.5-5224.2)	45 (12.2-76.1)	-0.21	-0.402--0.015
48402.6 (10176.2-92313.4)	49.3 (10.4-91.1)	-0.21	-0.398--0.022
165.8 (47.2-275.2)	63.1 (19.1-102.5)	-0.21	-0.268--0.152
7942.5 (1073.2-18163.3)	35.5 (5.5-76.2)	-0.21	-0.282--0.147
1362.4 (418.1-2350.2)	55.4 (17.9-87.5)	-0.21	-0.256--0.173
3444 (1024.5-5520.6)	45 (12.5-73)	-0.22	-0.272--0.166
1872.9 (586.9-2988.7)	63 (18.4-100.1)	-0.23	-0.278--0.174
1363.3 (387.1-2244.7)	41.5 (12.5-68.4)	-0.24	-0.431--0.041
6182.1 (1559.7-10840)	47.1 (11.6-85.2)	-0.24	-0.3--0.179
17.7 (4.8-28.2)	38.2 (9-62.7)	-0.25	-0.364--0.14
39.4 (10.2-64.9)	71.6 (22.5-116.3)	-0.27	-0.401--0.145
4.8 (1.3-7.8)	39.4 (10.4-65.5)	-0.27	-0.431--0.116
1645.2 (446.4-2611.7)	45.3 (11.9-73.5)	-0.28	-0.338--0.218
5759.7 (1434.3-9808.9)	48.6 (12.5-82)	-0.28	-0.393--0.166
15169.3 (2650.8-29127.3)	43.9 (10.2-83.9)	-0.29	-0.368--0.205
34.3 (9.3-54.3)	62.6 (19.6-102.6)	-0.32	-0.394--0.244
709.7 (211.6-1153.9)	41.9 (13-68.1)	-0.33	-0.362--0.304
115952.8 (78026.7-156379.1)	55.3 (37.3-74.2)	-0.34	-0.432--0.244
5041.9 (1093.2-9404.5)	50.6 (11.3-97.9)	-0.35	-0.436--0.262
4785.9 (1048.6-8909.5)	57 (12.8-105.2)	-0.35	-0.442--0.261
2296.9 (979.4-3419.6)	51.5 (20.6-77.9)	-0.38	-0.428--0.322
3651.9 (1555.4-5461)	39.9 (16.9-62.9)	-0.38	-0.455--0.309
40683.5 (27761.1-54324)	32 (21.6-43.9)	-0.39	-0.42--0.356
4618.9 (1246-7987.9)	45 (12.2-77.2)	-0.39	-0.478--0.3
13003.1 (4205.4-20695.3)	36.5 (11.2-59.4)	-0.39	-0.517--0.266
243.4 (62.8-384.8)	56.4 (17.5-88.9)	-0.4	-0.432--0.36
5120.7 (1279.6-9004.3)	21.7 (5.9-38.7)	-0.4	-0.595--0.21
978.8 (300.4-1614.8)	32.2 (9.4-54.4)	-0.46	-0.502--0.411
6646.8 (1499.3-13373)	47.7 (9.2-93.7)	-0.48	-0.671--0.293
2580.7 (747-4305.4)	31.1 (8.6-51.6)	-0.54	-0.61--0.467

Table S2

location	Num_1990	ASR_1990
Equatorial Guinea	1668.5 (300.3-3307.7)	402.5 (72.5-792.8)
Thailand	129610.9 (30186.9-242159)	230.6 (54.8-421.9)
Lesotho	3764.4 (860.8-7070.9)	247.6 (56.1-466.6)
Viet Nam	124899.7 (25487.7-239250.9)	178.9 (35.3-336.8)
Botswana	4599.1 (974.3-8271)	367.7 (80.4-643.9)
Mali	18117.6 (2513.8-37539.1)	210.9 (29.1-443.5)
Mauritius	5195.2 (1290.4-9183.3)	479.6 (119-852.3)
Cabo Verde	1372.2 (304.1-2661.7)	387.8 (85.5-749.7)
Dominican Republic	23883.7 (4985.5-41676.8)	327.5 (68.4-558.8)
Panama	10560.4 (2408.5-18763.4)	440.9 (100.4-778.5)
Saudi Arabia	62259.9 (15460.5-107142.9)	397.2 (98.9-684.9)
Sao Tome and Principe	426.6 (73.7-833.1)	342.1 (60.1-663.2)
Ghana	45416.2 (9411.2-84254.6)	304.1 (62.9-562.5)
Mozambique	43237.9 (7506.3-91102.7)	320 (56.6-676.2)
Eswatini	2730.8 (553.3-4917)	356.2 (71.9-638.6)
Oman	5272.5 (1129-9549.7)	253.4 (53.6-460)
Mongolia	7163.3 (1420.4-12376.6)	325.3 (64.7-561)
Chad	17442.7 (2387.1-37578.8)	282.2 (38.8-608.4)
Uzbekistan	84516.9 (21337.7-145917.9)	400 (102.4-689.6)
Estonia	5815.6 (1419.9-9404.4)	358.4 (89.1-576.7)
Turkmenistan	15026.1 (4274.4-25902.7)	388.1 (111.1-657.1)
Poland	114852.3 (81302.9-157164.4)	297 (210.3-405.2)
Guyana	3245.3 (812.9-5602.4)	428.1 (107.4-745.4)
Bosnia and Herzegovina	14688.6 (4231.2-26232.7)	326.3 (93.9-585.3)
Timor-Leste	1763.6 (375.9-3362.2)	219.4 (48.2-420.6)
Indonesia	351030.4 (233252.1-488320.4)	186.2 (124.2-258.7)
Germany	367472 (117956.4-569742.1)	456.7 (148-717.7)
Malta	915.3 (240.4-1515.5)	249.2 (65.2-417.2)
Myanmar	105509.6 (21218.4-202085.5)	252.2 (49.5-473.7)
Portugal	23234.6 (5210.7-39133)	229.3 (51.4-385.6)
Latvia	8562.4 (1999.8-13978)	311.3 (73.9-515.2)
Gambia	2837.1 (531.5-5590.4)	289.4 (56-571.1)
Namibia	4689.9 (1189.6-8202.8)	351.3 (89.1-612.3)
Senegal	27967.8 (4171.1-52767.7)	353.4 (52.7-658.8)
Israel	13430.4 (3567.3-21927.4)	268.3 (70.5-437.5)
China	178532.8 (1525446.5-2896308.[illegible]	189.3 (132.5-253)
Malaysia	54032.5 (16508.3-93209.9)	311.4 (95-530.8)
Singapore	6959.8 (2054.4-11711)	236.7 (70.1-392.1)
Ireland	12302.3 (3308.7-20139.6)	336.7 (90.1-550.7)
Belgium	38321 (11466-59712.7)	372.9 (109.2-584.4)
Uruguay	10807.9 (3186.1-17891.5)	346.9 (101.7-576.7)
United States of America	766684.5 (512602.8-1024431.2)	300.5 (199.4-403.4)

United Republic of Tanzania	97737.8 (21574.1-186721.9)	379.5 (82.5-731.6)
Seychelles	217.1 (59.6-375)	297 (82.9-514.4)
Netherlands	49351.3 (14437.4-74746.5)	310.8 (90.5-473.5)
Argentina	81831.6 (23342.7-132917.8)	248.3 (70.7-403.4)
Nigeria	354902.7 (224262.8-488252.2)	400.5 (254.1-555)
Lao People's Democratic Republic	9668.7 (1697.5-18487.9)	224.1 (39.6-427.7)
Angola	48549.3 (9035.9-94489.1)	487.4 (91.5-957.8)
Guatemala	37556.3 (7818.7-71234.7)	453.2 (93-851.8)
Uganda	67037 (10358.8-138268.6)	365.6 (55.7-755)
Taiwan (Province of China)	59003.2 (18145.5-96873.6)	298.9 (93.6-487.9)
Japan	294932.1 (201763.3-387878.1)	235 (160-312.5)
Iceland	718 (231.4-1127.4)	277.6 (89.3-436.8)
Guinea	21031.2 (3816-42491.7)	343.8 (62.4-682.6)
Tunisia	20865.6 (6133.2-36932.2)	234.4 (68.9-412.9)
Ivory Coast	48474.3 (9622.9-93954.6)	406.6 (80.7-782.5)
Bhutan	1708.7 (271-3398.6)	269.2 (43.2-525.6)
Eritrea	11751.6 (1985.7-23293.8)	349.9 (58.7-683.7)
France	233887.3 (76118-366908.8)	389.2 (127-614.7)
Burkina Faso	31938.6 (5221.3-68580.7)	331.6 (54.9-707.1)
Finland	16039.6 (5293.5-26153.6)	307.7 (99.9-498.8)
El Salvador	19831.1 (5166.5-35325.9)	364.9 (95.8-649.4)
Cambodia	22693.6 (4782-44894.7)	214.6 (45.8-422)
Kazakhstan	71534.3 (18721.6-118378.8)	436.4 (115.2-720.8)
Paraguay	14728.2 (3756.6-25445.9)	368.2 (93-631)
Belize	695.3 (143.1-1241.6)	368.7 (77.5-656.1)
Azerbaijan	31701.4 (7790.8-54293.6)	420 (104.4-716.4)
Luxembourg	1614.4 (510.2-2570.1)	408.7 (125-655.3)
Dominica	443.8 (107.8-753.8)	617.1 (150.9-1043.1)
Syrian Arab Republic	29663.7 (6474.9-53388.8)	213 (46.4-385.9)
Lithuania	13814.2 (4019.7-22800.5)	364.8 (105.9-601.1)
Monaco	111.3 (32.9-172.6)	335 (97.5-523.3)
Tonga	233.9 (59.2-414.6)	243.1 (62.2-419.4)
Fiji	2638.5 (737.7-4529.9)	354.4 (99.4-594.7)
Sudan	51626.9 (10420.6-100746.4)	228.6 (46.6-443)
Iraq	52296.7 (13177.7-97028.8)	262.6 (65-485)
American Samoa	159.8 (45.7-270.2)	348.6 (97.8-585.2)
Saint Vincent and the Grenadines	503 (113.6-887)	461.4 (104.6-813.9)
Suriname	1949.2 (443.6-3433.2)	505.3 (113.7-892.3)
Zambia	41233.3 (8892.6-76955.5)	515 (109.5-945.8)
Bulgaria	35339.1 (11089.1-57190.6)	403.6 (127.8-654.7)
Slovakia	23230.5 (6684.1-37673.2)	431.4 (124-703.4)
Morocco	68099 (15841.1-126329.8)	257.1 (59.6-474.5)
Spain	99221.8 (26502-167509.1)	254.9 (68.4-430)

Guam	437.8 (111-692.3)	335.9 (84.1-525.2)
Cameroon	39125.7 (7251.3-73998.6)	384.4 (74.7-724.2)
Philippines	150507.3 (102373-201047)	234.5 (163.5-312.5)
Samoa	466.6 (100-824.3)	285.7 (61.1-494.6)
Egypt	170130.5 (38148.2-319303.9)	285.8 (65-546.5)
Niue	7.7 (1.5-13.1)	335.8 (65.1-572.5)
Austria	25371.6 (7543-40664.9)	321.7 (96.3-515.6)
Canada	74577.5 (23244.7-117258.1)	263.5 (81.7-413.1)
Djibouti	1697.8 (331.3-3390.9)	411.7 (80.7-815.6)
Trinidad and Tobago	6929.6 (1896.3-11419.8)	582.6 (160.6-955.1)
Kenya	93581.2 (60340-129974.8)	400 (266.3-546.1)
India	2082161.7 (1311068-2906903.2	245.4 (156.6-341)
Pakistan	330876.8 (137833.7-573001.7)	301 (126.4-514.7)
Palestine	5957.9 (1125-11294.4)	270.6 (50.9-501.6)
Solomon Islands	796.3 (158.1-1501.5)	249.6 (50.6-471.8)
Vanuatu	359.3 (77.2-686.3)	248.6 (53.9-465.8)
Sri Lanka	78964.2 (17964.5-136502)	465.2 (105.3-795)
Marshall Islands	117.1 (26.5-214.3)	272.9 (62.6-498.1)
Sierra Leone	14443 (2142.4-27832.1)	337.7 (49.5-643.1)
Grenada	396.2 (104.8-701.8)	464.2 (121.7-819.9)
United Kingdom	237359.1 (173421.6-302514.6)	404 (293.1-517.2)
Nepal	65456.3 (11132.9-132556.4)	333.6 (57.1-665.3)
Benin	19252.9 (3450.2-38362)	382.7 (68.6-765.5)
Zimbabwe	35192.3 (7378.4-65214.9)	375.7 (80.3-689.9)
Cook Islands	65.2 (20.1-109.2)	355.4 (111-589.6)
Gabon	6427.5 (1332.8-11377.8)	675.6 (140.5-1193.1)
Ecuador	64723.3 (14751.7-113065)	681.1 (155.6-1180.9)
Czechia	46714.8 (14518.9-75623.9)	444 (136-717.4)
Papua New Guinea	9134.5 (2032.4-17721.8)	226.1 (50.2-442.7)
Costa Rica	14779.7 (4100-25074.3)	493.4 (138.7-831.1)
Tuvalu	25 (7-44.8)	269.5 (75-484.2)
Albania	14468.9 (4130.1-24909.8)	426.5 (121.7-731.1)
Tokelau	4.2 (1-7.5)	263.5 (62.7-474.4)
Ethiopia	125696.4 (46522.2-210709)	257 (93.3-431.1)
Mauritania	7713.6 (1766.7-14512.5)	382.8 (86.2-702.8)
Greece	27842.9 (8364.4-46299.9)	266.9 (80.7-439.9)
Lebanon	8842.1 (2309.1-15685.2)	288.1 (75.9-509)
Chile	64541.2 (19037.5-108798.8)	479.2 (142.7-810.1)
Jamaica	10431.9 (2967.4-17631.6)	436 (121.5-741)
Denmark	14132.3 (4278.4-22208.6)	270 (81.9-431.5)
Jordan	10971 (2987.8-19163.7)	271.6 (73.5-462.4)
Australia	54845.8 (16459.9-86161.1)	320.4 (95.6-503)
Palau	61.7 (13.9-104.8)	408.2 (93.2-694)

Congo	12193.7 (2400.7-23819.9)	525.4 (104.7-1005.8)
Iran (Islamic Republic of)	198462.4 (134739.1-264691.8)	310.1 (216.5-408)
Bangladesh	226311 (49676.8-425114.1)	202.4 (43.6-378.3)
Norway	18786.1 (11561.6-25102.5)	422.5 (262.8-572.3)
San Marino	69.9 (19-111.1)	289.8 (80.4-462.9)
Turkey	250633.3 (63212.8-448993.8)	415.8 (104-736.6)
United States Virgin Islands	538 (140.8-851.5)	513.8 (134.5-816)
Guinea-Bissau	4014.3 (581.2-7967)	403.1 (58.1-793.8)
Yemen	32058.5 (4830.2-65192.3)	204.8 (30.2-415.8)
Malawi	35628 (7669.2-69628.8)	353.7 (77.6-684.3)
Armenia	10474 (2966.6-18640.7)	305.8 (85.8-545.6)
Romania	97062.6 (27166.3-158605)	410.9 (114-676.9)
Comoros	1821.4 (340.5-3655.1)	398 (73.6-794.5)
Montenegro	2193.2 (675.8-3538.5)	349.5 (107.8-563.7)
Republic of Korea	146788.5 (39935.3-245141.4)	332.8 (91.4-549.7)
Antigua and Barbuda	424.8 (108.8-714.4)	699.8 (180-1178.5)
Micronesia (Federated States of)	303.2 (72.7-544.9)	305.9 (74.2-535.1)
Maldives	892 (169.2-1699.1)	401.3 (75.1-763.2)
North Macedonia	7265 (2195.5-11898.3)	363.7 (109.8-597.5)
Peru	121084.6 (30210.2-218187.6)	563.7 (142.6-995.6)
Italy	152751.2 (97844.6-203783.2)	263.1 (169.3-351.1)
Cyprus	2367.5 (641-3851)	304.4 (81.8-495.2)
Andorra	202.3 (67.4-320)	370.4 (125.8-578.3)
South Africa	159479.4 (99526.5-219586.7)	436.7 (274.6-605.3)
Niger	23478.8 (3051-51086.3)	283.4 (37.2-597.7)
Kuwait	6536.4 (1602.8-10918.4)	382.7 (94.4-630.3)
Bahrain	2156.5 (680.3-3682.9)	450 (142.7-756.8)
Switzerland	24527.3 (7727.2-38312.9)	340.5 (105.9-531)
Colombia	182671.9 (47269-309727.7)	549.7 (144.9-935)
Cuba	33256.3 (9400.7-56857.6)	300.3 (84.4-509.2)
Honduras	22841.8 (4226.8-43331.2)	482 (90.8-904.8)
Libya	13420.3 (3003.4-24165)	297.2 (66.1-529.6)
Saint Lucia	774 (212.7-1351.3)	581.4 (160.1-1004.5)
Kiribati	256 (64-463.7)	356.8 (87.6-639.7)
Kyrgyzstan	20633 (5120.7-35362.2)	460 (112.3-786.8)
Greenland	338.9 (94.4-568.2)	621.8 (173-1033.4)
Venezuela (Bolivarian Republic of)	107094 (24634.7-189781.4)	581.5 (136.9-1031)
Rwanda	29338.9 (5859.4-56046.9)	405.7 (79.5-767.4)
Somalia	18962.5 (2627-40570.5)	238 (34.2-504.4)
Central African Republic	9711.9 (1945.6-19150.8)	364.7 (72.5-707.2)
Nicaragua	16442.7 (3494.5-30320)	415.3 (92-761.6)
Madagascar	46982.8 (9125.4-91942.8)	384.2 (75-742.9)
Mexico	562695 (396935.7-745666.1)	660.8 (470.7-860.5)

Bahamas	1427.7 (406.7-2343.9)	562.3 (162.7-918.9)
Serbia	41017.8 (11644.9-67334.3)	425.8 (118.8-702.6)
Hungary	45344.7 (12574.6-73555.7)	429.2 (118.6-702.9)
Liberia	10612 (2055.7-20790.1)	416.4 (79-816.9)
Northern Mariana Islands	161.4 (37-261.4)	378.1 (86.4-615.5)
Barbados	1404.8 (432.2-2331.7)	537.1 (165.3-897)
New Zealand	12571.7 (4974.9-18749.8)	364.8 (145.4-547.1)
Sweden	25357.7 (10318.3-38318.9)	288.4 (117.4-441.7)
United Arab Emirates	10056.1 (2922.3-16545.9)	567.6 (170-928.3)
Nauru	42.1 (8.9-75.4)	437.6 (95.8-777.3)
Russian Federation	379498.4 (273213.8-492552.8)	248.9 (180.4-322.4)
Democratic Republic of the Congo	145676.6 (26062.8-280386.7)	382.1 (68.4-728.4)
Puerto Rico	19749.9 (6002.1-32483)	544.2 (165.4-892.4)
Haiti	24730.2 (4597.7-46911)	387.1 (70.7-726.8)
Ukraine	158540.3 (52685.6-253921)	301.5 (102-484.3)
Algeria	95397.9 (23294.5-171367.3)	344.4 (85.8-611.1)
Brazil	690901.2 (452706.6-944245.9)	465.9 (304.4-632.5)
Georgia	23182.2 (6000.6-38280.8)	423 (109.4-701.2)
Croatia	27032.4 (7047.6-43466.8)	534.8 (138.7-854.8)
Bermuda	332.1 (88-549.8)	552.8 (149-923.1)
Afghanistan	40056.6 (6995.5-78183)	389.5 (68-773.7)
Slovenia	9485.9 (2984.6-15310.8)	462.2 (144.7-742.3)
Tajikistan	27435 (6190.4-49156)	511.5 (116.5-923.1)
South Sudan	24516.5 (5144.7-47922.8)	412.8 (89.9-798)
Bolivia (Plurinational State of)	32632.9 (6784.3-58972.9)	526.5 (107.4-955.6)
Qatar	2085 (543.1-3465.4)	476.9 (128.5-787.1)
Saint Kitts and Nevis	289.9 (76.9-491.4)	712.2 (188.1-1197.6)
Brunei Darussalam	1336.5 (399.3-2141)	532 (157.7-861.4)
Togo	19314.2 (3938.2-37321.3)	511.7 (102.4-994.7)
Republic of Moldova	14254.8 (3716.5-24175.1)	319.3 (83.2-542.9)
Burundi	22191.4 (3713.1-44214.1)	399.2 (67.1-795.7)
Belarus	33257.3 (9486.2-55860.2)	314.3 (89.4-531.6)
Democratic People's Republic of Korea	52818.7 (11682.4-93848.1)	257.9 (57.7-454.5)

Num_2021	ASR_2021	AAPC	
9743.1 (2784.8-16073.1)	706 (172-1150.4)	1.84	1.731 - 1.946
228059.7 (59025.4-373733)	344.2 (111.1-559.4)	1.3	1.261 - 1.335
6466.9 (1612.8-11346.7)	360.1 (79.7-602.7)	1.2	1.124 - 1.271
240267.5 (61786.8-386501.5)	246.7 (56.2-409.7)	1.09	0.947 - 1.227
11068.8 (3003-18292)	513.1 (142.2-844.8)	1.06	1.005 - 1.121
61959.8 (11383.6-117911)	288.3 (64-547.2)	1.03	0.794 - 1.267
8201.2 (2423.4-12972.8)	630.3 (207.5-981)	0.9	0.856 - 0.939
2634.8 (622.6-4654.9)	500.8 (125.8-858.8)	0.87	0.771 - 0.967
44471.4 (12023.8-75909.8)	423 (129.2-696.3)	0.84	0.765 - 0.919
23415 (7001.9-38184.1)	571.5 (170.7-897.7)	0.84	0.803 - 0.873
177653.3 (52438.6-279397.1)	518 (145.1-805.9)	0.83	0.744 - 0.91
877.4 (227.9-1519.1)	439 (133.7-723)	0.81	0.769 - 0.846
120471.1 (32428.3-202290.3)	387.6 (104.4-634.9)	0.79	0.701 - 0.869
116322.1 (27208.5-219309.8)	409.6 (77.4-763.8)	0.77	0.606 - 0.938
4839.2 (1322.4-8177)	449 (107.6-751.3)	0.76	0.669 - 0.847
12649.5 (3829.7-20222.4)	321.8 (89-497.8)	0.74	0.695 - 0.779
12453.7 (3596.2-21021.1)	403 (82.2-672.8)	0.69	0.635 - 0.737
56722.1 (10330.3-108756.7)	342.1 (60.3-649.1)	0.63	0.519 - 0.748
149415.6 (41055.7-242691.5)	479.2 (142.1-765.4)	0.59	0.454 - 0.715
6229.9 (1788.6-9967.4)	420.7 (126.8-632.4)	0.57	0.43 - 0.71
23855.6 (5789.1-38157.9)	459.8 (115.9-748.7)	0.55	0.503 - 0.592
140005.1 (89572.3-184832)	350.7 (236.8-458.2)	0.53	0.521 - 0.548
3608.1 (956.9-6019.7)	502.4 (138.6-804.2)	0.51	0.478 - 0.546
13253.3 (4169.4-21257.6)	379.1 (107-617.1)	0.51	0.432 - 0.587
3246.6 (743.9-5756.5)	251.7 (69-428.9)	0.5	0.274 - 0.73
542468.9 (366005.5-732647.4)	219 (147.8-294.1)	0.48	0.439 - 0.525
582520.5 (223598.6-855770.8)	538.6 (163-815.6)	0.48	0.246 - 0.714
1341.6 (373.8-2109.5)	290.5 (82.7-458.4)	0.48	0.348 - 0.61
157488.9 (38343.8-268290.9)	292.2 (84.4-518.6)	0.47	0.341 - 0.606
32466.6 (9736.1-51426)	269.2 (73.8-420.7)	0.46	0.362 - 0.558
7493.4 (1835.8-11602.3)	362.8 (117-571.2)	0.46	0.389 - 0.529
7100.5 (1830-12739.2)	334.6 (77.4-578)	0.46	0.427 - 0.486
9242 (2680-15302.4)	405.5 (123.2-688)	0.46	0.437 - 0.475
60449 (16553.7-105860.4)	400.8 (89.7-717.9)	0.44	0.27 - 0.61
28456.7 (7493.8-45473.6)	307.6 (105.5-466.1)	0.44	0.386 - 0.486
2653418.5 (1904378-3456797.7)	214.7 (150.1-278.6)	0.43	0.279 - 0.585
103710.8 (25431.3-168239.5)	353.4 (94.8-559.1)	0.43	0.391 - 0.469
14910.3 (3918.3-23126.2)	271.3 (78.8-431.8)	0.43	0.41 - 0.448
18667.4 (5304.8-28663.3)	382.9 (113-589.7)	0.43	0.26 - 0.598
56184.8 (18882.4-86144.1)	425.6 (125.9-656.7)	0.42	0.396 - 0.451
14284.7 (3888.4-23189.5)	394.4 (95.3-636.9)	0.42	0.39 - 0.452
1171416.1 (773159.1-1553777.4)	341.6 (217.9-466.9)	0.4	0.297 - 0.506

229767 (61881.8-394168.6)	425.8 (103-749)	0.39	0.044 - 0.734
337.7 (83.3-553.9)	331.8 (94.3-560.3)	0.37	0.231 - 0.504
64462.3 (23540.1-96396.8)	347.3 (99.4-537.7)	0.36	0.251 - 0.477
122929.2 (43175-195637)	277.7 (71.5-454.7)	0.36	0.297 - 0.429
922861.4 (678892.5-1181455.9)	443.4 (326.7-580.9)	0.36	0.245 - 0.479
16881.8 (4169.7-31383.3)	252.3 (60.5-427.9)	0.36	0.312 - 0.41
159338.5 (39078.2-275776.8)	542 (142.7-917)	0.35	0.312 - 0.382
78196.9 (21185.5-133475.1)	505.2 (140.3-844.9)	0.35	0.238 - 0.453
159877.3 (32419.4-294545.3)	404.7 (85.8-722.6)	0.34	0.272 - 0.414
85721.5 (28637.6-130546.8)	334.2 (84.6-509.7)	0.34	0.228 - 0.453
367649.3 (239472.7-493975.7)	261.6 (169-352.3)	0.34	0.266 - 0.405
1120.6 (280.3-1742.1)	308.1 (88-484.8)	0.33	0.303 - 0.354
45884.6 (9029.5-84967.2)	374.1 (81.7-702.2)	0.32	0.231 - 0.414
28184.1 (7528.5-46182.5)	257.9 (75.8-429.9)	0.31	0.253 - 0.375
110233.4 (24640.8-199409.1)	446.6 (109.5-797.9)	0.31	0.258 - 0.355
2197.9 (581.8-3944.7)	297.6 (78.9-525.4)	0.3	0.216 - 0.377
22955.7 (5237.2-40124)	380.1 (98.2-676.6)	0.29	0.13 - 0.453
328152 (114023.3-488111.4)	426.9 (155.3-661.3)	0.28	0.237 - 0.324
78209.5 (15876.6-155834.1)	362.1 (66.6-692.2)	0.27	0.21 - 0.336
20385.5 (6677.6-30915.4)	336.3 (91.5-529.4)	0.27	0.221 - 0.322
25321.4 (5413.8-42775.2)	401.8 (98.6-658.9)	0.27	0.139 - 0.4
37829.5 (9668.4-67062.6)	233.4 (73.5-429)	0.27	0.225 - 0.307
85478.8 (25648.3-136369.7)	475.8 (136.7-769.9)	0.27	0.173 - 0.359
27820.5 (6254.9-45774.2)	399.9 (124-668.6)	0.26	0.204 - 0.314
1624.3 (495.2-2815.1)	401.3 (116.3-686.1)	0.25	0.189 - 0.317
46829.6 (13870.7-75019.5)	449.6 (133.3-745.2)	0.25	0.103 - 0.389
2955.6 (854.8-4500.5)	439.9 (139.5-663)	0.25	0.213 - 0.276
445.4 (112.2-715.4)	665.6 (224.1-1029.3)	0.24	0.217 - 0.268
30369.6 (9094.4-52374.4)	229.3 (61.2-381)	0.24	0.069 - 0.409
11840.9 (3090.9-18625.9)	389.2 (123.9-586.2)	0.24	0.186 - 0.284
153.7 (44.7-238.5)	356.4 (121.8-564)	0.23	0.14 - 0.316
261.8 (68.1-436.9)	261.6 (79.9-422.3)	0.22	0.17 - 0.263
3402.1 (922.2-5587.9)	381.3 (111.3-598)	0.21	0.144 - 0.269
104902.6 (30258.3-177101.1)	242.3 (64.5-411.8)	0.2	0.151 - 0.249
109932.9 (31932.2-179364)	278.9 (83.4-449.8)	0.2	0.108 - 0.284
187 (56.7-300.6)	366.2 (92.7-594.3)	0.19	-0.013-0.389
553 (155.9-919.1)	494.2 (132.8-812.4)	0.19	0.095 - 0.281
3058 (923.1-4897.2)	542.9 (184.4-868.1)	0.19	0.086 - 0.286
97029.1 (25914.7-166945.8)	547.3 (147.9-944.1)	0.19	0.095 - 0.276
30253.7 (10153.1-46465.4)	429.1 (118.8-682)	0.18	0.12 - 0.247
26477.5 (7392.8-41699.5)	458.3 (136.9-727.4)	0.18	0.122 - 0.241
97094.7 (25268-165185.6)	281.1 (69.5-485)	0.18	0.04 - 0.313
131162.9 (38757.4-205471)	269.3 (85.1-429.5)	0.16	-0.042-0.37

578.4 (161.5-890.6)	351.4 (123.2-567.2)	0.14	0.1 - 0.188
117836.5 (25343.7-210467.9)	402.8 (99.8-694.6)	0.14	-0.025-0.303
257362.5 (186239.9-334564.2)	243.5 (175.2-319.5)	0.12	0.033 - 0.214
565.4 (138.5-961.3)	295.3 (90.6-476)	0.12	0.099 - 0.147
297113.4 (104527.1-474061.2)	303.1 (81-484.3)	0.12	-0.01-0.244
5.7 (1.7-9.2)	347.9 (108.6-551.5)	0.12	0.045 - 0.19
29701 (9119.9-45548.6)	332.7 (99.5-511.7)	0.11	0.093 - 0.126
108492.5 (32716.9-166022.6)	272.3 (76.4-430.2)	0.11	-0.011-0.23
4783.8 (1066.3-8494.6)	429.9 (113.8-738)	0.11	0.077 - 0.144
8470.9 (2195.7-13660.4)	593.4 (168.6-955.2)	0.1	0.022 - 0.173
185143.1 (133528.2-239943.8)	411.9 (308-527.6)	0.09	0.04 - 0.144
3596343.5 (2611912.5-4693453.2)	256.2 (185.6-325.7)	0.09	-0.025-0.201
665973.3 (361365.9-1012095.6)	308.5 (157.1-447.3)	0.09	0.056 - 0.121
14125.8 (4378.6-23962.6)	279 (87.4-468.9)	0.09	0.04 - 0.136
1670.6 (338.9-2863)	256.7 (61.9-458.6)	0.08	-0.002-0.171
746.2 (161.4-1293)	254.8 (56.8-433.2)	0.08	0.056 - 0.103
105492.8 (33949.4-173566)	476.5 (117.2-777.7)	0.08	0.026 - 0.131
154.1 (40.9-267.9)	278.6 (67.4-492.3)	0.07	0.006 - 0.138
28500.4 (6432.4-52167.2)	346.4 (73.4-636.3)	0.07	-0.049-0.194
487.9 (134.2-766.3)	477.2 (131.8-790.3)	0.07	0.046 - 0.087
281683.1 (192826-367347.3)	413.6 (276.6-554)	0.06	-0.021-0.145
97224.9 (20212.1-176999.5)	339.5 (68.8-609.1)	0.06	-0.052-0.168
48944.2 (11618.2-88431.5)	391.2 (96.6-719.4)	0.06	-0.056-0.171
53197.7 (12078.1-92538.7)	380.2 (89.8-677.6)	0.05	-0.051-0.15
65.4 (19.6-102.2)	364.6 (115.3-578.1)	0.04	-0.036-0.12
11853.2 (2981.9-19422.9)	688.2 (146.3-1158)	0.04	-0.068-0.149
123880.9 (40232.3-200904)	710.8 (226.2-1140.8)	0.04	-0.23-0.306
51470 (15848.4-78952.7)	450.3 (126.9-694.6)	0.03	-0.024-0.088
21947.1 (5903.5-39761.5)	227.3 (54.7-402.6)	0.03	-0.036-0.097
22908.1 (6656.1-37882.7)	495.7 (150-804)	0.03	-0.019-0.071
31.5 (9-54.9)	272.2 (74.7-438.5)	0.02	0.001 - 0.043
11353.2 (3127.4-18562.9)	437.1 (113.6-705.2)	0.02	-0.172-0.21
3.6 (1.1-6)	268.2 (64.5-454.5)	0.01	-0.108-0.125
257198.7 (139003.5-388720.1)	257.5 (139.1-372.4)	0.01	-0.11-0.121
15257 (4109.5-26000.8)	384.2 (106.1-649.1)	0.01	-0.08-0.091
28053.6 (9143.5-44006.5)	267.3 (68.7-422.8)	0	-0.082-0.09
15459.6 (4586.3-24627.6)	289.8 (93.5-476.6)	0	-0.029-0.035
89070 (21865.6-143661.2)	478.8 (148.1-773.6)	-0.02	-0.054-0.014
12205.9 (3617.4-19958.3)	426.3 (124.7-693.1)	-0.02	-0.121-0.073
17001.3 (5355.6-25972.9)	266.1 (74.9-413.4)	-0.03	-0.175-0.126
30251.1 (9608.5-48966.4)	268.8 (74-436)	-0.04	-0.137-0.057
82298.5 (23155.3-127272.2)	316.5 (100.8-512)	-0.04	-0.114-0.032
72.9 (21.5-115.6)	403.1 (115.7-631.4)	-0.04	-0.056--0.025

25711.7 (5739-42934.9)	515.4 (144.1-887.6)	-0.04	-0.125-0.039
238568.5 (168893.1-310929.6)	305.4 (211.4-392.6)	-0.05	-0.085--0.019
294799.3 (71006-536606.3)	199.3 (59-356.3)	-0.07	-0.172-0.023
23570.9 (14024-32030.2)	413.3 (247.4-567.6)	-0.08	-0.111--0.043
91.1 (26.6-138.3)	284.9 (78.7-448.8)	-0.08	-0.109--0.055
329890.1 (115002.7-509961.8)	405.8 (127.1-642.6)	-0.1	-0.279-0.09
489 (134-761.6)	500.4 (152.1-777.4)	-0.12	-0.233--0.012
7107.2 (1665.6-13319.4)	385.9 (71.8-712.7)	-0.13	-0.225--0.033
65078.4 (17093.8-118538.6)	197.4 (36.9-352.4)	-0.13	-0.196--0.062
62716.8 (13803.3-116429.3)	338.2 (75.6-610.6)	-0.14	-0.163--0.107
8225 (2046.4-13544)	296.5 (95.5-496.2)	-0.14	-0.183--0.1
77343.7 (24134.1-122218.4)	398.1 (121.7-617.6)	-0.15	-0.273--0.025
2712.2 (657.3-4711.9)	378.8 (108.6-661.6)	-0.15	-0.249--0.051
2020.1 (568.7-3282.4)	333.3 (89.4-532.7)	-0.15	-0.179--0.122
164736.6 (47087.9-253656.6)	315.4 (84.6-484.7)	-0.19	-0.23--0.155
591.9 (171-933.6)	662.3 (195.6-1027.4)	-0.2	-0.254--0.149
286.5 (82.9-485.7)	289.1 (62.6-488.8)	-0.21	-0.232--0.177
1632.6 (424.5-2796.8)	376.5 (120.1-631.7)	-0.21	-0.277--0.139
7505.9 (2333.7-12147.5)	341.9 (96.4-539.1)	-0.22	-0.238--0.192
176866.2 (56660.1-298146.2)	534.4 (155.1-856.1)	-0.22	-0.351--0.092
170110 (111724.2-225480.9)	253.4 (174.1-344.3)	-0.22	-0.455-0.009
3753 (1129.9-5723.6)	287.6 (81-450.5)	-0.23	-0.356--0.094
291.8 (69-439.5)	348.7 (100.7-530.1)	-0.23	-0.283--0.175
220980.4 (143621.4-305558.5)	407.3 (266.3-544.6)	-0.24	-0.474--0.007
60675.6 (10480.4-125024.2)	264.6 (48.4-540.1)	-0.24	-0.275--0.211
13658.2 (3866.5-21945.5)	358.1 (95-569.3)	-0.25	-0.353--0.136
5555.3 (1421.8-9084.3)	417.2 (115.7-663.3)	-0.25	-0.321--0.174
30772.7 (9239-46728.8)	314.1 (92.6-495.1)	-0.26	-0.306--0.218
239231.2 (62856.3-395645.3)	504.4 (135.2-858.9)	-0.29	-0.332--0.237
28042.9 (7225.5-45764.6)	270.1 (78.5-445.2)	-0.34	-0.403--0.273
40850.1 (10531.4-73502.9)	437.9 (96.9-757)	-0.35	-0.453--0.246
17267.6 (5163-28447.6)	266.7 (80.2-428.6)	-0.35	-0.402--0.298
977.4 (270.1-1574.6)	519.3 (139.4-852.3)	-0.35	-0.405--0.294
364.2 (87.3-615.9)	317.8 (79.1-551.9)	-0.36	-0.416--0.294
27191.8 (8885.9-45280.2)	416.9 (112.5-660.2)	-0.36	-0.463--0.246
334 (100.6-522.3)	554.6 (167.4-870.9)	-0.36	-0.432--0.285
151955.8 (44680.6-247432.2)	520.8 (149-889.5)	-0.37	-0.426--0.313
43889.8 (10919.9-75952.7)	360.5 (77.4-634.1)	-0.37	-0.415--0.33
40996.1 (5688.4-92690.7)	211.5 (34.2-459.8)	-0.38	-0.434--0.329
17429.8 (3165.7-32923.4)	325.7 (65-600.6)	-0.4	-0.487--0.309
23374.3 (6256.9-40214.3)	364.8 (85.9-645.8)	-0.42	-0.454--0.388
87756 (22963.1-156065.7)	337.3 (88.4-620)	-0.42	-0.454--0.39
734266.5 (534829.5-958489.3)	582.8 (400.6-753.8)	-0.42	-0.461--0.385

1939.7 (516.6-3061.3)	493.1 (155.9-765.5)	-0.44	-0.505--0.364
35137.3 (10280-57022.1)	371.2 (109.1-567.6)	-0.45	-0.486--0.403
36726 (12668.1-56452.2)	375 (118.2-588.8)	-0.45	-0.512--0.388
18347 (3861.3-34065.1)	365.3 (72.6-657.7)	-0.46	-0.623--0.298
153.6 (43.3-245.4)	326.8 (80.8-516.1)	-0.47	-0.542--0.388
1413.1 (399-2237)	463 (148-739.6)	-0.49	-0.55--0.434
15699 (6962.5-23459.2)	312.9 (122.1-471.4)	-0.51	-0.553--0.47
26637.4 (11016.1-39873.1)	245.2 (105.3-363.5)	-0.51	-0.586--0.442
42953 (13968.3-68508.3)	482.4 (148.1-748.3)	-0.52	-0.594--0.442
41.3 (11.2-66.8)	376.9 (99.3-611.3)	-0.53	-0.722--0.334
312836.1 (214189.9-415384)	211.2 (143.6-281.8)	-0.55	-0.594--0.514
290190.2 (60104.2-531337.3)	327 (73.1-584.1)	-0.56	-0.827--0.292
16767.9 (5205.5-26028.7)	460.9 (129.5-727)	-0.57	-0.651--0.482
39875.6 (10267.8-68824.9)	324.3 (80.6-567.8)	-0.57	-0.663--0.481
110318.7 (36663.7-172190.4)	253.1 (77.6-403.7)	-0.59	-0.723--0.451
121203 (35089.5-202228.1)	284.4 (72-471.3)	-0.59	-0.629--0.55
843931.1 (598845.6-1142885.9)	388.9 (262.2-526.5)	-0.6	-0.675--0.521
13092.3 (3899.6-20874.4)	357.5 (110.3-573.4)	-0.61	-0.754--0.456
20717.9 (5587.4-32121.7)	443.8 (122.6-705.1)	-0.61	-0.657--0.57
299.9 (85.2-476.9)	452.6 (139.4-709.8)	-0.63	-0.682--0.577
95793.9 (18065.6-182133.5)	317.9 (70.6-594.2)	-0.66	-0.753--0.57
8106.2 (2572.8-12694.7)	376.7 (120.6-590.9)	-0.66	-0.694--0.634
38380.4 (10443.3-64317.9)	417.8 (113-698.8)	-0.67	-0.727--0.62
29116.1 (6342.3-54765.5)	334.8 (76.4-623.4)	-0.68	-0.724--0.629
48156.8 (12898.7-79408.1)	421.1 (105.8-704)	-0.7	-0.794--0.596
9058.5 (2762.6-14937.7)	381.3 (122.2-587.3)	-0.71	-0.772--0.644
337.6 (81.8-529.4)	564.3 (166.3-909.6)	-0.74	-0.789--0.687
1837.9 (467.3-2852.9)	418.3 (132.5-644.5)	-0.77	-0.796--0.733
30324.3 (6250.2-55167.7)	399.6 (86.9-733.2)	-0.77	-0.898--0.647
9049.6 (2781.2-14841)	247.5 (76.3-407.6)	-0.84	-0.883--0.793
37854.8 (8912.2-74509.5)	306.8 (57.7-602.2)	-0.85	-0.95--0.753
23102.6 (6856.2-36770.4)	234.2 (65.1-384.3)	-0.92	-1.101--0.737
45540.1 (11673.5-77884.8)	177.8 (47.7-304.9)	-1.16	-1.285--1.043

Table S3

location	Num_1990	ASR_1990	Num_2021
Italy	393.9 (377.6-408.9)	0.6 (0.6-0.6)	1438.1 (1244-1568.4)
Greece	55.5 (52.3-59.1)	0.5 (0.4-0.5)	178.2 (161.6-192.4)
Japan	422.3 (411.7-430.9)	0.3 (0.3-0.3)	1660.5 (1415.9-1797.4)
Northern Mariana Islands	0.1 (0.1-0.3)	0.3 (0.2-0.6)	0.3 (0.2-0.4)
Poland	472.2 (460.1-486.6)	1.2 (1.1-1.2)	1041.5 (1008-1070.3)
Germany	1046.8 (1009.7-1089.4)	1.1 (1.1-1.1)	3127.1 (2805.1-3347.3)
American Samoa	0.4 (0.3-0.7)	0.9 (0.6-1.6)	0.7 (0.5-0.8)
Denmark	64.3 (58.8-69.8)	1 (0.9-1.1)	132.7 (119.6-144.5)
Malta	2.1 (2-2.3)	0.5 (0.5-0.6)	5.7 (5.1-6.3)
Belgium	153 (144.3-161.1)	1.3 (1.2-1.3)	372.7 (335.3-403.8)
Guam	0.3 (0.2-0.6)	0.2 (0.2-0.4)	0.6 (0.4-0.8)
Zambia	443 (336-543.8)	8.9 (7.2-10.7)	1701.6 (1236.6-2284.6)
Tokelau	0 (0-0)	1.5 (1.1-2.3)	0 (0-0)
Spain	286.5 (271.6-301.6)	0.7 (0.6-0.7)	830.2 (725.6-910)
Turkmenistan	68.6 (60.2-78.4)	1.9 (1.7-2.2)	148.2 (102.2-199.1)
Iceland	1.9 (1.8-2.1)	0.7 (0.7-0.8)	4.8 (4.3-5.3)
Chad	149.6 (100.2-216.3)	3.8 (2.5-5.5)	492.3 (323.9-683.1)
Israel	38.6 (35.9-41.5)	0.8 (0.8-0.9)	128.7 (117.8-138.8)
Portugal	136.2 (128.7-144.9)	1.3 (1.2-1.3)	322.7 (288.7-347.9)
Monaco	0.2 (0.1-0.3)	0.5 (0.4-0.6)	0.3 (0.2-0.4)
Kazakhstan	246 (224.8-268.7)	1.5 (1.4-1.7)	381.2 (345.5-420.9)
Lesotho	29.4 (20.7-41.7)	2.4 (1.7-3.5)	52.4 (37.9-69.6)
Bulgaria	137.9 (126.3-151)	1.5 (1.4-1.7)	167.4 (145.6-192.5)
Netherlands	179.1 (167.5-191.5)	1 (1-1.1)	370.1 (335.4-398.8)
United States of America	1715 (1666.9-1755.7)	0.6 (0.6-0.6)	3451.3 (3263.7-3568)
Austria	76.8 (72.6-81.3)	0.8 (0.8-0.9)	165.8 (150.9-178.6)
Taiwan (Province of China)	162.3 (153.5-170.9)	0.8 (0.8-0.8)	305.8 (284.1-326.9)
Papua New Guinea	54.8 (29.7-82.7)	1.5 (0.8-2.3)	173.2 (115.8-258.1)
Mauritius	26.7 (24.9-28.6)	2.9 (2.7-3.2)	51.1 (46.9-55.1)
Gambia	22.8 (15.4-32.5)	3.7 (2.6-5.3)	74.7 (53.2-100)
Czechia	165.4 (154-176.8)	1.5 (1.4-1.6)	232.1 (207.8-257.6)
Brazil	1726.6 (1662.2-1797.6)	1.3 (1.3-1.4)	3644.6 (3503.2-3775.1)
Guinea	172.1 (121.3-233.8)	3.9 (2.7-5.4)	390.2 (251.7-518)
Uruguay	48.8 (46-51.8)	1.5 (1.4-1.6)	73.8 (68.4-78.7)
Finland	69.2 (64.7-73)	1.2 (1.1-1.2)	124.3 (111.4-135.2)
Senegal	200 (153.8-259.8)	4 (3-5.2)	539.2 (384.3-710.6)
Sao Tome and Principe	2.5 (1.9-3.4)	3.1 (2.2-4.2)	5.6 (4-7.6)
Zimbabwe	258.2 (207.6-366.2)	4 (3.2-5.2)	511.4 (337.7-735)
Singapore	12.4 (11.7-13.1)	0.4 (0.4-0.4)	27.2 (25-29.5)
Georgia	81.4 (73.8-89.7)	1.5 (1.3-1.6)	61.1 (55-67.2)
Niue	0 (0-0)	1.2 (0.9-1.8)	0 (0-0)
Ghana	217.7 (164.3-297.9)	2.1 (1.6-3)	612.8 (418.1-794.6)

Togo	78.8 (59-104.6)	3.5 (2.6-4.8)	235.9 (152.2-324.2)
Jamaica	44 (40.2-48.2)	2.1 (1.9-2.3)	62.4 (48.6-78.5)
Sierra Leone	108.1 (77.5-148)	3.6 (2.5-5.1)	240.5 (151.5-336.6)
Guinea-Bissau	32.3 (23.7-41.3)	5 (3.6-6.5)	74.1 (52.4-98.4)
Mali	263.2 (188.2-360.8)	4.5 (3.2-6.3)	712.1 (491.2-953.8)
Ivory Coast	276.9 (214.5-373.7)	3.7 (2.9-5.1)	780.8 (474.9-1056.1)
Niger	219.1 (148.3-318)	4.4 (2.9-6.5)	640 (373.7-984.1)
Vanuatu	1.8 (1.2-2.9)	1.4 (0.9-2.3)	4.4 (3.3-5.9)
Ireland	44.4 (41.8-47.5)	1.2 (1.1-1.3)	93 (84.2-101.8)
Venezuela (Bolivarian Republic (	375.2 (358-395.7)	2.3 (2.2-2.4)	616.4 (499.9-764.4)
Eritrea	182.5 (136.2-230.5)	8.1 (6-10.2)	356 (251.9-483.3)
Solomon Islands	4.6 (2.7-6.9)	1.7 (1-2.6)	11 (7.5-16)
Luxembourg	6.9 (6.4-7.3)	1.5 (1.4-1.6)	15.6 (14.1-17.3)
Uzbekistan	524.9 (482.2-572.1)	2.6 (2.4-2.9)	900.3 (810.7-1001.9)
Burkina Faso	254.3 (172.1-381.5)	4.2 (2.7-6.3)	620.9 (390.1-851.4)
Benin	119.8 (91-156.3)	3.9 (2.9-5.1)	331.9 (216.1-447.5)
Central African Republic	114.6 (87.1-152.6)	5.4 (4-7.3)	238.5 (167.2-338.5)
Costa Rica	41.5 (38.8-44.5)	1.7 (1.5-1.8)	82.2 (76.2-89.1)
Nauru	0.1 (0.1-0.2)	1.6 (1.2-2.2)	0.2 (0.1-0.2)
Guyana	24.8 (21.5-27.9)	4 (3.5-4.5)	27.1 (21-33.9)
Switzerland	97.5 (87.4-110.6)	1.1 (1-1.3)	170.8 (150.4-190.5)
Kyrgyzstan	133.4 (119.6-148.6)	3.1 (2.8-3.5)	201.5 (176.6-226.8)
France	1180 (1124.6-1229.6)	1.7 (1.6-1.8)	2076.1 (1828.9-2285.7)
South Sudan	322.6 (238.3-439.1)	8.9 (6.4-12.5)	519.3 (363.9-714.8)
Cameroon	264.7 (214.2-343)	3.9 (3.1-5.2)	824.2 (496.3-1144)
Pakistan	3843.3 (2757.1-4670.7)	4.4 (3-5.5)	7952.1 (6164.2-10007.8)
Paraguay	54.1 (46-70.2)	1.5 (1.3-2)	111.3 (81.5-137.7)
North Macedonia	32.6 (26.3-45.7)	1.6 (1.3-2.3)	34.7 (27.7-41.7)
Somalia	479 (331-722.6)	10.4 (7.1-15.9)	1243.7 (783.2-2020.6)
Croatia	78.6 (72.1-85.5)	1.5 (1.4-1.6)	87.9 (77.6-98.7)
Djibouti	16.9 (12.8-22.5)	7.2 (5.4-9.7)	58.2 (40.8-83.3)
Tajikistan	238.9 (205.1-296)	4.8 (4.1-6.1)	468.8 (351-632.8)
Marshall Islands	0.6 (0.5-0.8)	1.6 (1.2-2.3)	0.9 (0.6-1.1)
Nigeria	2053 (1543.7-2689)	3.3 (2.4-4.4)	4782 (2833.7-6432.9)
Romania	415.7 (392.5-440.6)	1.8 (1.7-1.9)	382.2 (349.2-414)
Burundi	324 (253.5-402.9)	8.8 (6.9-11.4)	608.3 (460.8-791.8)
Mauritania	48.8 (37.8-69.2)	3.5 (2.8-4.9)	97.5 (67.8-124.9)
Eswatini	21 (15.7-27.5)	3.6 (2.7-4.8)	34.3 (22.5-46.9)
Kenya	879.5 (667.1-1098.2)	6.7 (4.8-8.6)	1974.2 (1579.6-2439.6)
Philippines	218.8 (170.1-246.5)	0.4 (0.3-0.5)	389.4 (286.8-442.4)
Bosnia and Herzegovina	93.1 (78.9-123.1)	2 (1.7-2.7)	79 (52.8-95.8)
Namibia	33.7 (25.7-43.2)	3.2 (2.4-4.1)	59.6 (42.3-82.5)
Palau	0.3 (0.2-0.4)	2.1 (1.6-2.8)	0.4 (0.3-0.5)

Indonesia	560 (138.3-818.8)	0.4 (0.1-0.6)	897.6 (219.4-1385.2)
Argentina	279.6 (264.9-294)	0.9 (0.8-0.9)	418.8 (390.8-449.4)
Slovenia	31 (28-34.4)	1.4 (1.3-1.6)	38.1 (33.7-42.8)
Democratic Republic of the Con[illegible]	1235.3 (940.2-1669)	4.3 (3.3-5.9)	2619.6 (1894.7-3756)
Kiribati	1.8 (1.4-2.3)	2.6 (2-3.3)	2.6 (1.9-3.6)
Fiji	15.4 (11.5-23.6)	2.2 (1.6-3.3)	17.4 (12.7-23.5)
South Africa	779.5 (610-928)	2.4 (1.9-2.8)	1196.3 (1025.5-1433.4)
Slovakia	123.9 (106.3-159.1)	2.2 (1.9-2.9)	126.1 (97.5-144.9)
Panama	36.3 (33.5-39.8)	1.7 (1.6-1.9)	63.2 (57.5-68.9)
Tonga	0.9 (0.7-1.2)	1.2 (0.9-1.5)	1 (0.7-1.3)
Uganda	665.2 (493.4-928.9)	6.6 (4.8-9.1)	1501.2 (1138.2-1970.6)
United States Virgin Islands	2 (1.4-2.5)	2 (1.4-2.4)	1.7 (1.2-2.2)
Saint Vincent and the Grenadine	3.6 (3.2-3.9)	3.9 (3.5-4.3)	3.9 (3.5-4.4)
Dominica	2.7 (2.4-3.3)	4.2 (3.7-5.1)	2.7 (2.1-3.4)
Samoa	2 (1.4-2.9)	1.4 (1-2.1)	2.2 (1.7-3.1)
Montenegro	5.7 (4.8-7)	0.9 (0.8-1.1)	5.6 (4.4-6.7)
Hungary	172.9 (160.8-185.5)	1.5 (1.4-1.6)	162.3 (147.7-178.8)
Congo	79.1 (59.1-102.5)	4.4 (3.4-5.6)	160.6 (115.4-215.1)
Comoros	25.2 (17.6-31.8)	8.4 (6.3-10.5)	40.4 (30.6-56)
Azerbaijan	195.4 (164.8-250.2)	2.7 (2.2-3.5)	239.4 (180.7-326.7)
Chile	230 (218.4-242.1)	1.8 (1.7-1.9)	326.1 (305.4-345.6)
Egypt	485.2 (419.3-623.9)	1 (0.8-1.3)	721.5 (534.7-812.3)
Micronesia (Federated States of)	1.5 (1.1-2.2)	1.8 (1.3-2.5)	1.5 (1.1-2)
Syrian Arab Republic	147.8 (104.1-188)	1.4 (1-1.7)	143.5 (98.1-192.4)
Mozambique	802.3 (649.8-1034.1)	9.6 (7.8-12.2)	1436.2 (1051.7-2025.8)
Tuvalu	0.1 (0.1-0.2)	1.6 (1.3-2.3)	0.2 (0.1-0.2)
Canada	302.9 (289.2-316.6)	1 (1-1.1)	408 (375.9-433.6)
Liberia	84.8 (65.1-111.8)	4.7 (3.6-6.2)	139.3 (86.7-203.4)
Malawi	629.5 (470.5-756.4)	10.5 (8-12.8)	1085 (816.3-1357.6)
Thailand	680 (565.4-818.1)	1.5 (1.2-1.8)	997 (779.1-1179.6)
Belize	3.6 (3.3-3.9)	2.2 (2-2.4)	7.7 (6.9-8.8)
Norway	80.7 (77.4-84)	1.6 (1.5-1.6)	108.7 (101.2-114.9)
Nepal	836.5 (571.3-1108.4)	6.8 (3.7-9.1)	1245.4 (857.2-1655.3)
Madagascar	545 (455.9-686.9)	7.2 (6-9)	1055.5 (786.3-1392)
Sweden	109.7 (104.1-116.1)	1.1 (1-1.1)	124.2 (114.2-132.9)
Viet Nam	52.2 (9.5-205.7)	0.1 (0-0.4)	85.9 (18.4-345.8)
Australia	215.8 (204.9-228.3)	1.2 (1.1-1.3)	299.7 (277.1-318.4)
Botswana	37.1 (25.3-54)	3.7 (2.5-5.4)	65 (48.6-82)
Cuba	120.8 (115.4-127.3)	1.1 (1.1-1.2)	124.1 (115.2-132.7)
Bhutan	15.5 (9.4-20.9)	3.4 (2.3-4.7)	19.1 (12.8-27.3)
Angola	400.3 (296.1-516)	5.1 (3.8-6.6)	921.2 (682.2-1239.2)
Timor-Leste	4.9 (3-8.6)	0.8 (0.5-1.5)	6.8 (4.4-13.4)
Ecuador	291.8 (274.7-308.4)	3.3 (3.1-3.5)	343.7 (303.7-387.6)

Colombia	559.8 (525.8-591.3)	1.9 (1.8-2)	738.8 (697.4-777.8)
Seychelles	1.1 (0.9-1.3)	1.7 (1.4-2)	1.4 (1.1-1.7)
Democratic People's Republic of	255.6 (172-388.8)	1.3 (0.9-1.9)	263.1 (184-416.5)
United Republic of Tanzania	1551.4 (1257.2-1867.3)	9.4 (7.4-11.4)	2873.9 (2230.6-3722.1)
United Kingdom	1046.7 (1020.5-1066.4)	1.6 (1.6-1.6)	1083.6 (1032.8-1121.7)
Cambodia	72.7 (54.4-116.5)	0.9 (0.7-1.5)	97.8 (61.1-184)
Yemen	259.9 (142.3-371)	2 (1.3-2.8)	439.2 (283-598.6)
Suriname	12.1 (9.2-14.3)	3.6 (2.8-4.3)	17.1 (14.7-21)
Latvia	61.4 (53.7-69.9)	2.2 (1.9-2.5)	33.5 (28.9-39.5)
Haiti	298.3 (224.7-414.7)	5.8 (4.5-7.7)	454.1 (335.6-640.3)
Rwanda	450.9 (363.7-565.8)	9.6 (7.9-12.1)	613.8 (453.5-864)
Honduras	167 (137.3-195.3)	4.1 (3.5-4.9)	259.4 (185.6-358.5)
Gabon	32.7 (26.5-41.2)	4.3 (3.5-5.4)	48.5 (33.9-68.1)
Armenia	33.4 (30.1-36.2)	1 (0.9-1.1)	26.6 (24.3-29.2)
Cabo Verde	11.1 (8.8-13.9)	4.6 (3.6-5.7)	17.4 (13.2-22.4)
Dominican Republic	123.5 (102.9-147.6)	2 (1.7-2.5)	164.2 (129.8-217.8)
Brunei Darussalam	3.6 (2.8-4.9)	1.7 (1.3-2.3)	5.6 (4.7-6.7)
Mexico	1942.5 (1879-2008.5)	2.6 (2.6-2.7)	2337.8 (2256.3-2423.5)
Lithuania	104.6 (94.6-115.5)	2.7 (2.5-3)	63.9 (57.3-71.9)
Serbia	208.7 (178.6-259.3)	2.1 (1.8-2.6)	177.6 (129.8-204.9)
Albania	88.1 (70.9-121.9)	2.8 (2.2-4.1)	63.4 (48.3-89.9)
Barbados	6.9 (6.3-7.5)	2.6 (2.4-2.8)	7 (5.8-8.4)
India	25738.5 (15953.5-30648.2)	3.6 (2.2-4.3)	32694.7 (24185.5-36778.5
New Zealand	56.5 (53.5-59.9)	1.6 (1.5-1.7)	61.3 (57.4-65.5)
Trinidad and Tobago	38.4 (36-41.2)	3.7 (3.4-3.9)	38.3 (31.8-44.5)
Bahamas	4.9 (4.4-5.5)	2.2 (1.9-2.4)	6.2 (5-7.6)
Malaysia	146.4 (121.9-170)	1.1 (0.9-1.3)	215.5 (186.6-257)
Mongolia	55.4 (43.3-69.6)	2.7 (2.2-3.4)	61.7 (47.9-77.6)
El Salvador	72.3 (57.5-83.6)	1.5 (1.2-1.7)	67.4 (52-76.3)
Antigua and Barbuda	2.8 (2.5-3)	4.9 (4.5-5.4)	3.6 (3.3-4.1)
Greenland	1.8 (1.3-2.2)	3.6 (2.5-4.4)	1.7 (1-2.1)
Afghanistan	455.3 (317.8-605.2)	5 (3.6-6.7)	862.3 (639.2-1119.5)
Lao People's Democratic Republ	31.4 (21.5-52.9)	0.9 (0.6-1.6)	40 (25.3-79)
Cook Islands	0.2 (0.1-0.3)	1 (0.7-1.4)	0.1 (0.1-0.2)
Myanmar	473.9 (237.1-650.3)	1.4 (0.8-1.9)	521.3 (302.4-718.5)
Libya	59.6 (46.5-75.6)	1.6 (1.3-2.1)	69.6 (47.5-102)
Saint Lucia	5 (4.7-5.4)	4.6 (4.4-5)	5.7 (4.8-6.7)
Ukraine	686.4 (660.7-711.9)	1.3 (1.2-1.3)	449.5 (370.9-543.3)
Guatemala	265 (247.7-283.2)	3.7 (3.5-3.9)	365.6 (343.7-389)
Ethiopia	3623.3 (2580-4553.3)	12.1 (9-14.7)	5141 (4361.4-6085.3)
Grenada	2.7 (2.4-3)	3.6 (3.2-4)	2.4 (2.2-2.7)
Morocco	613.3 (453.1-734.4)	2.9 (2.1-3.5)	627.1 (461.7-798.9)
Sudan	501.5 (336.5-736.5)	2.5 (1.8-3.3)	650.1 (453.9-880)

Oman	10.8 (7.7-14.7)	0.7 (0.5-0.9)	17.3 (12.4-21.4)
Equatorial Guinea	15.3 (11.4-21.8)	4.8 (3.5-6.7)	33.7 (20.6-51.3)
Andorra	0.6 (0.4-0.8)	1 (0.8-1.4)	0.9 (0.7-1.2)
Saudi Arabia	319.6 (237.2-409.4)	2.5 (1.9-3.2)	539.3 (339-716.5)
Republic of Moldova	118.2 (108-128.5)	2.7 (2.5-2.9)	72.2 (63-82.6)
Nicaragua	74.1 (60.7-85.7)	2.1 (1.9-2.6)	90.5 (78.9-112)
Bahrain	10 (8.6-12.7)	2.7 (2.3-3.2)	19.4 (16.4-25.2)
Algeria	445.7 (333.8-541.4)	2 (1.6-2.4)	473.9 (388.8-584.9)
Tunisia	130.6 (99.5-160.2)	1.7 (1.3-2.1)	115.1 (84.2-161.2)
Bangladesh	1642.9 (1241.8-2283.4)	1.9 (1.4-2.5)	1513.2 (1134.3-2026.6)
Bolivia (Plurinational State of)	199.4 (147.7-253.7)	3.5 (2.7-4.5)	230.4 (164.1-317.9)
Palestine	51.6 (40.8-66.1)	3 (2.4-3.8)	71.8 (61-92.3)
Lebanon	77.8 (57.5-104)	2.9 (2.2-3.8)	101 (84.3-124.3)
Kuwait	15.3 (13.9-16.9)	1 (1-1.1)	24.4 (22.5-26.6)
Russian Federation	1232.3 (1203.3-1259.9)	0.8 (0.8-0.8)	829.7 (794.3-854.1)
Republic of Korea	654.6 (578.5-884.1)	1.6 (1.4-2.2)	565.9 (425.4-634.8)
Belarus	183.4 (164.5-202.3)	1.7 (1.5-1.9)	105.6 (95.1-117.7)
Estonia	54 (47.3-62.1)	3.3 (2.9-3.8)	28.3 (24.8-32.2)
Iraq	289.7 (227.1-353.2)	1.7 (1.4-2.1)	335 (250.2-428)
San Marino	0.1 (0.1-0.1)	0.2 (0.2-0.3)	0.1 (0.1-0.1)
Bermuda	1.1 (1-1.2)	1.8 (1.6-1.9)	0.9 (0.7-1)
Maldives	2.7 (2-3.7)	1.9 (1.4-2.4)	4.6 (3.5-7.8)
Puerto Rico	90.7 (84.1-98.3)	2.5 (2.3-2.7)	56.2 (50.9-61.3)
Turkey	2015.7 (1569.4-2488.8)	3.7 (3-4.6)	1511.8 (1254.6-1786.9)
Cyprus	10.3 (8.8-12.4)	1.5 (1.2-1.7)	12.7 (10.5-14.8)
Sri Lanka	432.3 (350.3-499.5)	3.2 (2.6-3.7)	356.1 (265.8-493.3)
Saint Kitts and Nevis	1.8 (1.6-1.9)	4.9 (4.5-5.3)	1.4 (1.2-1.7)
Jordan	50.4 (40.7-60.7)	1.6 (1.3-1.9)	74 (61.4-89.9)
Iran (Islamic Republic of)	913.8 (637.3-1067.4)	1.7 (1.3-1.9)	699.4 (624.6-876.2)
China	21495.6 (18263-25955.7)	1.9 (1.6-2.2)	12204.1 (9876.6-15189.5)
Peru	377.8 (279.8-440.1)	1.9 (1.4-2.2)	317.7 (253.8-436)
Qatar	4.5 (3.5-5.5)	1.6 (1-1.9)	15.6 (11.6-23.8)
United Arab Emirates	35.5 (20.4-49.3)	2.1 (1.4-2.9)	61.5 (47.1-79.1)

ASR_2021	AAPC	
1.2 (1-1.4)	2.52	1.779 - 3.27
0.9 (0.9-1)	2.38	1.935 - 2.834
0.7 (0.6-0.7)	2.36	1.249 - 3.486
0.6 (0.4-0.7)	1.92	0.458 - 3.397
2.2 (1.7-2.5)	1.9	0.831 - 2.987
1.9 (1.7-2)	1.78	1.324 - 2.229
1.4 (1-1.7)	1.44	0.85 - 2.03
1.4 (1.2-1.5)	1.32	0.5 - 2.139
0.8 (0.7-0.9)	1.31	0.576 - 2.049
1.9 (1.7-2.1)	1.28	0.566 - 1.996
0.4 (0.2-0.5)	1.23	0.637 - 1.828
12.9 (9.5-17.1)	1.22	0.933 - 1.516
2 (1.7-2.6)	1.17	0.551 - 1.795
1 (0.8-1.1)	1.15	0.563 - 1.746
2.8 (1.9-3.9)	1.15	0.997 - 1.293
1 (0.9-1.1)	1.07	0.357 - 1.786
5.1 (3.4-7)	1.06	0.912 - 1.198
1.1 (1-1.2)	1.01	0.587 - 1.43
1.6 (1.4-1.8)	0.93	-0.092-1.954
0.6 (0.4-0.8)	0.92	0.774 - 1.059
1.9 (1.7-2.2)	0.91	0.185 - 1.639
3.2 (2.2-4.2)	0.9	0.737 - 1.07
2 (1.5-2.6)	0.89	0.249 - 1.526
1.4 (1.2-1.5)	0.88	0.381 - 1.391
0.8 (0.7-0.8)	0.86	0.499 - 1.218
1.1 (1-1.1)	0.74	-0.097-1.582
1 (0.9-1.1)	0.67	-0.044-1.396
1.8 (1.2-2.7)	0.6	0.528 - 0.667
3.5 (3.1-3.8)	0.59	-0.388-1.577
4.5 (3.1-6.1)	0.57	-0.201-1.342
1.7 (1.3-2.1)	0.55	-0.29-1.395
1.5 (1.5-1.6)	0.54	0.281 - 0.806
4.5 (3-6.1)	0.5	0.413 - 0.581
1.8 (1.6-1.9)	0.5	-0.345-1.344
1.3 (1.2-1.5)	0.48	0.15 - 0.82
4.6 (3.2-6.2)	0.45	-0.043-0.954
3.4 (2.4-4.5)	0.42	0.251 - 0.597
4.5 (3-6.3)	0.41	0.218 - 0.609
0.5 (0.4-0.5)	0.39	-0.188-0.971
1.5 (1.2-1.8)	0.38	-0.579-1.356
1.4 (1.1-1.7)	0.37	-0.108-0.851
2.4 (1.6-3.1)	0.37	0.188 - 0.545

3.9 (2.5-5.4)	0.36	0.091 - 0.632
2.1 (1.5-2.7)	0.35	-0.435-1.15
4 (2.5-5.7)	0.33	0.072 - 0.593
5.5 (3.8-7.2)	0.32	0.214 - 0.429
5 (3.3-6.9)	0.32	0.214 - 0.421
4.1 (2.5-5.6)	0.31	0.074 - 0.539
4.8 (2.8-7.6)	0.28	0.084 - 0.476
1.6 (1.2-2.1)	0.28	0.025 - 0.528
1.3 (1.1-1.4)	0.27	-0.169-0.704
2.2 (1.6-2.8)	0.22	-0.335-0.77
8.6 (6.4-11.4)	0.21	0.103 - 0.314
1.8 (1.2-2.6)	0.18	0.064 - 0.287
1.6 (1.4-1.9)	0.16	-0.401-0.719
2.6 (2.2-3.1)	0.16	-0.535-0.853
4.4 (2.7-6.1)	0.15	-0.032-0.329
4 (2.6-5.5)	0.13	0.016 - 0.239
5.6 (4-7.8)	0.12	-0.029-0.27
1.7 (1.5-1.9)	0.11	-0.218-0.443
1.7 (1.3-2.2)	0.11	0.029 - 0.191
3.6 (2.7-4.6)	0.11	-1.133-1.359
1.2 (1-1.3)	0.07	-0.245-0.384
3.1 (2.5-3.7)	0.06	-1.449-1.597
1.7 (1.5-1.8)	0.04	-0.23-0.313
9 (6.5-12.5)	0.04	-0.215-0.286
3.9 (2.3-5.5)	0.02	-0.056-0.095
4.4 (3.1-5.8)	0.02	-0.074-0.111
1.5 (1.1-2)	-0.01	-0.213-0.198
1.6 (1.1-2.1)	-0.01	-0.341-0.327
10.3 (6.7-16.9)	-0.02	-0.131-0.098
1.6 (1.1-2)	-0.02	-0.625-0.583
6.8 (5-9.5)	-0.09	-0.287-0.113
4.6 (3.4-6.1)	-0.14	-0.527-0.247
1.5 (1.2-2)	-0.14	-0.19--0.089
3.2 (1.9-4.3)	-0.14	-0.272--0.016
1.7 (1.3-2)	-0.17	-0.987-0.659
8.5 (6.4-11.4)	-0.18	-0.323--0.039
3.3 (2.3-4.3)	-0.18	-0.593-0.228
3.4 (2.3-4.6)	-0.19	-0.298--0.08
6.1 (4.7-7.5)	-0.27	-0.349--0.193
0.4 (0.3-0.5)	-0.28	-0.558-0.007
1.8 (1.2-2.3)	-0.3	-0.661-0.071
2.9 (2.1-3.9)	-0.3	-0.36--0.245
1.9 (1.5-2.5)	-0.31	-0.37--0.245

0.4 (0.1-0.6)	-0.34	-0.464--0.22
0.8 (0.7-0.8)	-0.36	-1.062-0.345
1.4 (1-1.8)	-0.36	-0.751-0.031
3.9 (2.7-5.7)	-0.37	-0.555--0.175
2.3 (1.7-3.1)	-0.4	-0.462--0.342
1.9 (1.4-2.6)	-0.42	-0.515--0.331
2.1 (1.8-2.4)	-0.43	-0.698--0.156
1.9 (1.5-2.4)	-0.43	-0.666--0.187
1.5 (1.2-1.7)	-0.43	-0.944-0.08
1 (0.7-1.4)	-0.47	-0.597--0.343
5.7 (4.4-7.4)	-0.47	-0.622--0.324
1.6 (1.1-2.2)	-0.48	-1.179-0.223
3.2 (2.7-3.6)	-0.48	-0.954--0.005
3.6 (2.8-4.7)	-0.49	-0.566--0.42
1.2 (0.9-1.6)	-0.5	-0.557--0.449
0.7 (0.6-0.9)	-0.52	-0.707--0.336
1.3 (1-1.6)	-0.53	-1.488-0.431
3.6 (2.5-4.8)	-0.53	-0.891--0.174
7.2 (5.3-10.2)	-0.54	-1.572-0.511
2.4 (1.7-3.3)	-0.55	-0.932--0.171
1.4 (1.3-1.6)	-0.57	-1.29-0.161
0.8 (0.6-1)	-0.58	-0.804--0.36
1.5 (1.1-1.9)	-0.6	-0.621--0.576
1.2 (0.8-1.6)	-0.63	-0.862--0.406
7.9 (5.9-10.6)	-0.64	-0.8--0.484
1.3 (1-1.7)	-0.65	-0.7--0.604
0.8 (0.7-0.8)	-0.66	-0.897--0.421
3.8 (2.3-5.5)	-0.67	-0.905--0.425
8.5 (6.4-10.7)	-0.67	-0.893--0.44
1.2 (0.9-1.5)	-0.67	-0.921--0.424
1.8 (1.5-2.1)	-0.68	-1.481-0.134
1.4 (1.3-1.4)	-0.68	-1.446-0.094
5.5 (3.1-7.6)	-0.7	-0.791--0.607
5.7 (4.4-7.7)	-0.71	-0.783--0.631
0.8 (0.7-0.9)	-0.72	-2.55-1.146
0.1 (0-0.3)	-0.73	-0.867--0.598
0.9 (0.8-1)	-0.76	-0.954--0.564
2.9 (2.1-3.7)	-0.8	-0.996--0.606
0.8 (0.7-0.9)	-0.81	-1.457--0.162
2.7 (1.8-3.9)	-0.82	-1.067--0.576
3.9 (2.8-5.1)	-0.84	-1.09--0.582
0.6 (0.4-1.3)	-0.86	-1.209--0.518
2.2 (1.5-2.9)	-0.87	-2.029-0.313

1.4 (1.2-1.7)	-0.89	-1.54--0.24
1 (0.8-1.2)	-0.9	-1.049--0.743
0.9 (0.7-1.5)	-0.9	-0.954--0.85
7 (5.5-9.2)	-0.93	-1.011--0.842
1.2 (1.1-1.3)	-0.95	-1.75--0.144
0.7 (0.4-1.2)	-0.97	-1.021--0.916
1.5 (1-2)	-0.97	-1.352--0.591
2.6 (2-3.3)	-0.98	-1.319--0.645
1.5 (1.1-1.9)	-1.01	-2.184-0.183
4.2 (3.1-5.8)	-1.01	-1.133--0.896
6.8 (5.1-9.7)	-1.03	-1.725--0.339
3 (2.1-4)	-1.05	-1.178--0.916
3.1 (2.2-4.3)	-1.06	-1.366--0.747
0.8 (0.6-1)	-1.06	-2.162-0.055
3.3 (2.5-4.2)	-1.07	-1.229--0.911
1.4 (1.1-1.9)	-1.07	-1.291--0.856
1.2 (1-1.4)	-1.09	-1.248--0.925
2 (1.7-2.3)	-1.11	-1.575--0.646
1.9 (1.5-2.3)	-1.11	-1.855--0.363
1.4 (1.1-1.7)	-1.13	-1.281--0.979
2 (1.5-2.9)	-1.14	-1.376--0.896
1.7 (1.3-2.1)	-1.14	-1.699--0.581
2.5 (1.8-2.8)	-1.14	-1.442--0.839
1 (1-1.1)	-1.2	-2.058--0.332
2.4 (1.8-3)	-1.21	-2.557-0.163
1.5 (1.2-1.9)	-1.21	-1.634--0.793
0.7 (0.6-0.9)	-1.22	-1.418--1.011
1.8 (1.4-2.3)	-1.22	-1.657--0.773
1 (0.8-1.3)	-1.23	-1.426--1.03
3.2 (3-3.5)	-1.25	-2.363--0.13
2.4 (1.6-3.1)	-1.25	-1.609--0.899
3.3 (2.5-4.3)	-1.26	-1.543--0.968
0.6 (0.4-1.3)	-1.27	-1.318--1.216
0.6 (0.5-0.9)	-1.28	-1.348--1.211
1 (0.6-1.3)	-1.32	-1.43--1.2
1.1 (0.7-1.5)	-1.32	-1.495--1.142
2.8 (2.3-3.3)	-1.35	-1.873--0.82
0.9 (0.6-1.1)	-1.4	-3.342-0.578
2.2 (1.9-2.6)	-1.46	-2.427--0.48
7.6 (6.2-9.2)	-1.49	-1.551--1.42
2.2 (1.9-2.5)	-1.51	-2.804--0.195
1.8 (1.3-2.2)	-1.51	-1.672--1.348
1.6 (1.1-2.1)	-1.52	-1.622--1.408

0.4 (0.3-0.5)	-1.52	-1.82--1.221
2.9 (1.9-4.4)	-1.55	-1.963--1.134
0.7 (0.4-0.9)	-1.56	-1.761--1.364
1.5 (1.1-1.9)	-1.6	-1.702--1.497
1.7 (1.3-2)	-1.62	-2.876--0.353
1.3 (1.1-1.6)	-1.63	-1.813--1.448
1.6 (1.3-2)	-1.65	-1.997--1.296
1.1 (0.9-1.5)	-1.71	-1.806--1.608
1 (0.7-1.4)	-1.73	-1.804--1.664
1.1 (0.8-1.4)	-1.76	-2.079--1.446
2 (1.4-2.8)	-1.78	-1.823--1.742
1.7 (1.5-2.2)	-1.79	-2.083--1.486
1.7 (1.4-2)	-1.79	-1.918--1.668
0.6 (0.5-0.7)	-1.85	-3.442--0.23
0.5 (0.4-0.5)	-1.88	-3.258--0.477
0.8 (0.7-1)	-1.96	-2.179--1.741
0.9 (0.7-1.1)	-2	-2.526--1.468
1.7 (1.4-2.1)	-2.02	-2.698--1.346
0.9 (0.7-1.1)	-2.11	-2.312--1.913
0.1 (0.1-0.2)	-2.19	-2.447--1.94
0.9 (0.8-1.1)	-2.22	-2.589--1.84
1 (0.8-1.4)	-2.22	-2.517--1.928
1.2 (1-1.4)	-2.29	-2.875--1.696
1.8 (1.4-2.2)	-2.32	-2.542--2.097
0.7 (0.6-0.8)	-2.35	-2.792--1.911
1.5 (1-2.1)	-2.45	-2.646--2.25
2.3 (1.9-2.8)	-2.46	-3.278--1.628
0.7 (0.6-0.9)	-2.54	-2.778--2.309
0.7 (0.7-1)	-2.57	-2.712--2.424
0.8 (0.7-1)	-2.65	-2.876--2.423
0.8 (0.6-1.1)	-2.8	-3.522--2.077
0.6 (0.5-0.8)	-2.85	-3.241--2.457
0.9 (0.6-1.1)	-2.94	-3.774--2.102

Table S4

location	Num_1990	ASR_1990
Lesotho	3153.6 (1764.6-5090.8)	211.3 (119.7-334.7)
Zambia	43203.7 (26981.9-63643.3)	555.1 (363.6-802.7)
Chad	14996.9 (7446.6-25759.2)	276.2 (148.8-457.2)
Tokelau	2.8 (1.4-4.6)	181.1 (94.5-295.2)
Turkmenistan	9844.6 (5990.3-15406.4)	249.2 (150-383.4)
Japan	92184.5 (55975.2-142383.7)	74.7 (45.7-115.7)
Mauritius	3131.4 (1787.3-4958.4)	289.7 (167.2-456.5)
Gambia	2363.4 (1271.8-3842.7)	272.5 (155.1-428.8)
American Samoa	80.9 (34.7-147.9)	171.4 (72-311.2)
Poland	57282.5 (41969.3-78737)	147.7 (108.3-203.1)
Greece	8611.3 (3551.3-17629.7)	82.8 (33.9-173.6)
Mali	21072.8 (12274.6-33161.7)	279.7 (166.9-428.3)
Zimbabwe	27204.6 (15334.6-43271.8)	301.1 (177-466.6)
Senegal	21949.9 (11617-35846.3)	312.4 (176.9-495.4)
Ghana	29909.9 (15148-48846.3)	211.8 (113.9-342.1)
Malta	317.9 (137.9-627.8)	86.4 (36.9-172.7)
Viet Nam	49976.1 (12310.6-108917.6)	71.1 (18-152.1)
Sao Tome and Principe	295.9 (145.5-498.8)	263.7 (142.2-427.7)
United States of America	261530.3 (170520.9-382726.3)	102 (66.1-153.1)
Belgium	15115.7 (7419-26885.1)	147.8 (71.2-266.8)
Iceland	247.9 (115.3-458.1)	96 (44.5-177.1)
Guinea	17612.2 (9383.8-29473.4)	312.4 (174.6-503.5)
Papua New Guinea	6986.2 (3324.4-11196)	169.8 (80.5-277.4)
Niue	4.2 (1.9-7)	186.5 (86.1-308.3)
Netherlands	18392.8 (9332.5-32153.9)	117.7 (59.9-204.3)
Ivory Coast	34587.8 (17686.3-60588.4)	316.7 (174.7-523.7)
Israel	5261.2 (2401.8-9548.4)	105.6 (48.8-191)
Eswatini	2331.6 (1340.8-3583.5)	308.8 (179-472.9)
Bulgaria	17088.7 (9117-28463.3)	199.9 (108-334.3)
Thailand	80758.6 (42902.1-131648.1)	144.4 (77.5-234.4)
Vanuatu	255.3 (127.9-398.6)	174.1 (87.1-268.7)
Germany	135073.1 (65995.6-253737.6)	168.8 (80.9-316.9)
Italy	51526.4 (33104.5-75731.4)	89.8 (57.9-133.8)
Monaco	32.9 (11.5-67.1)	102.5 (37.1-205.5)
Guam	163.4 (55.2-300.9)	121.7 (40.7-225.5)
Sierra Leone	11598.3 (5863.6-19406.5)	293.5 (158.4-475.7)
Burkina Faso	26207.5 (13050.6-44989.3)	309.4 (167.5-513.9)
Uruguay	5580.4 (2949-9308.9)	180 (95.1-300.5)
South Sudan	26226.7 (16119.3-40134.4)	479.9 (302.1-710.8)
Guinea-Bissau	3487 (1861.9-5730.6)	389.3 (221.6-606.4)
Kazakhstan	38043.3 (19336.8-63728.1)	228.7 (115.4-382.9)
Indonesia	170704.9 (101569.4-255391.7)	90.9 (53.4-136.3)

Solomon Islands	611.9 (298.3-1067.3)	190.7 (93.1-322.1)
Uzbekistan	62752.3 (39011.5-96738.1)	288.5 (178.2-443.9)
Guyana	2658.1 (1600.7-3987.9)	352.7 (213.4-522.3)
Panama	5557.2 (2744.9-9281.1)	228.7 (113.6-384.2)
Cameroon	29642.2 (15933.7-48181.2)	317.9 (186.6-506.2)
Botswana	3992.8 (2385.5-6136.2)	321.5 (194.2-493.4)
Central African Republic	11152.3 (6812.2-16679.3)	418 (257.6-620.7)
Somalia	36820.4 (23829.8-54670.7)	515.6 (334.7-773.5)
Niger	22115.8 (11546.8-36972.4)	307.8 (170.5-497.4)
Nigeria	243587.7 (170177.8-335049.3)	294.2 (208.6-397.4)
Uganda	63202.6 (38274.8-99985.6)	381.9 (230-594.3)
Ireland	4859.9 (2462.3-9241.5)	133.9 (68-254.5)
Denmark	5997.7 (3263.4-10374.7)	113.3 (60-198.8)
Djibouti	1653.8 (1003.8-2572.7)	431.5 (270.9-648.7)
Benin	14412.9 (7308.5-24660.2)	321.3 (182.6-526.6)
Marshall Islands	82.8 (42.4-138.3)	192.9 (101.3-319.7)
Spain	36616.2 (16991.7-70472.2)	94.8 (43.8-186.2)
Philippines	70320.6 (45654.1-100259.6)	107 (70.4-151.8)
Finland	6939.7 (3930.5-12473.4)	131.4 (72.7-237.6)
Kenya	83918.8 (62555.8-108474.7)	390.2 (291.6-497)
Tonga	140.3 (69.7-233.6)	147.5 (75.3-238.7)
Fiji	1928.1 (1083.3-2982.5)	249 (138-387.5)
Namibia	3772.5 (2127.9-5722.8)	285.2 (165.9-426.1)
Dominica	285.7 (161.2-456.2)	403.2 (231.2-632.7)
Austria	9212.3 (4393-17533.9)	117 (54.8-223.6)
Eritrea	15911.7 (10681.3-22845.7)	498.8 (343.8-701.9)
Taiwan (Province of China)	26712.5 (13234-48092.1)	131.4 (63.7-237.6)
Pakistan	354779.1 (246839.9-493051.8)	314.5 (219.7-438.3)
Timor-Leste	1051.5 (425.6-1848.7)	127.7 (49.7-223.4)
Portugal	12340.1 (7335.9-20262.1)	124.3 (74.2-202.3)
Singapore	2641.7 (1102.7-4991.2)	88.3 (35.9-166.5)
United States Virgin Islands	274.6 (139.5-445.6)	259.6 (131.1-420.3)
Mozambique	61471.5 (41730.8-88447)	482 (336.5-671.9)
Equatorial Guinea	1626.6 (933.1-2652.7)	397.8 (226.6-637)
Paraguay	8440.4 (4221.3-14137.6)	206.1 (102-350.4)
Dominican Republic	16731.4 (9289.2-27007.8)	224.2 (123.8-361.7)
Samoa	292.6 (138.8-474.5)	177.9 (85.3-282.7)
Palau	41.4 (23.5-61.8)	259.5 (145.3-392.1)
Jamaica	5696.8 (3045.8-9381.5)	240.1 (130-392.6)
Argentina	41311.5 (20290.3-69104.3)	125.6 (62-209.5)
Bosnia and Herzegovina	9160 (5550.4-14040.3)	200.7 (120.6-309.9)
Costa Rica	6748.1 (3181.4-11975.1)	225 (106.7-396.5)
Northern Mariana Islands	61.9 (18.5-119.5)	138.9 (38.6-275.9)

Czechia	20508.1 (11008.2-37043.3)	195.9 (104.5-353.8)
Luxembourg	676.6 (362.1-1173.8)	171.5 (90.4-304.3)
France	101919.6 (58160.6-167073.9)	169.8 (95.9-281.3)
Canada	30127.6 (17208.1-51069.4)	106.6 (60.7-181.3)
Mauritania	5617.9 (3028.7-9638.7)	301.1 (173.6-494.3)
Togo	12165.9 (5532-21722.7)	355.2 (180.8-604.4)
Tajikistan	25435.7 (17220.1-38054.1)	454.8 (303-687.4)
Oman	2505 (926.6-4729.7)	119 (45.5-222.1)
Myanmar	69593.7 (27261.2-119833.4)	169.7 (69.3-289.6)
Lao People's Democratic Republic	6165.3 (2426.8-11105.5)	141 (54.9-253.7)
Malawi	50157.4 (33242.2-69523.2)	539.6 (367.2-733.1)
Saint Vincent and the Grenadines	370.9 (224.5-576.4)	347.7 (215.9-531.1)
United Republic of Tanzania	125689.7 (85754.7-172653.2)	507.1 (356.1-685.6)
Angola	45217 (26021.5-71241.6)	449.8 (260.5-721.9)
Kiribati	218.6 (133.6-338.6)	292.1 (176.4-452.9)
Syrian Arab Republic	20328.9 (11006.1-32869.3)	145.8 (80.3-234.7)
Malaysia	27361.5 (12271.2-46681.2)	157.5 (71.5-266.9)
Cambodia	14135 (6023.8-27179.8)	131.5 (55.9-249.7)
Nauru	25.1 (11.2-43.1)	254.1 (113.1-435.8)
Azerbaijan	23442 (14596.4-37135.8)	303.2 (187.1-483.9)
Belize	467.9 (271.2-735.4)	242.5 (139.1-381)
Cabo Verde	1029.3 (583.2-1663.4)	336.8 (206.8-521.5)
San Marino	18.1 (5.5-39.5)	75.9 (23.2-163.8)
Slovakia	12473.7 (7207-20100.5)	232.1 (133.6-373.8)
Congo	9747.5 (5468.2-16122.4)	422.5 (242.3-682)
Micronesia (Federated States of)	215 (115.8-356.7)	214.9 (118.1-352.9)
Tuvalu	18.4 (10-29)	196.3 (106.6-311.7)
Seychelles	128.8 (69-212.4)	179.7 (98.4-293.3)
Switzerland	9217.5 (4709.1-16134.5)	128.3 (63.5-228.6)
Madagascar	49173.5 (31424.8-74397)	429.8 (288.6-628.3)
Democratic Republic of the Congo	135541.7 (76702.6-209330.4)	360.6 (207.6-547)
Montenegro	857 (424.9-1482.5)	136.5 (67.4-237)
Bhutan	1645.5 (933.7-2556)	257.8 (146.7-398.4)
Georgia	11488.2 (6045.1-19210.2)	210.5 (110.6-353.2)
Venezuela (Bolivarian Republic of)	59480.4 (30232.1-101393.2)	317 (160.4-539)
Kyrgyzstan	15347.9 (9617.8-23429.2)	336.5 (210.7-514.7)
Comoros	2147.8 (1346.5-3156.9)	489.7 (317.4-707.1)
Suriname	1381.4 (748.7-2212)	358.4 (201.9-565.9)
South Africa	102452.1 (75206-137536.8)	276.3 (201.3-374.9)
Burundi	27284.2 (18086.5-38977.3)	514.8 (335.2-719.2)
North Macedonia	3925.8 (2178.5-6444.8)	196.5 (109.6-321.9)
United Kingdom	103799.4 (76892.8-141221.9)	179.2 (133-242.6)
Australia	23463.6 (13074.6-39991.6)	137.1 (76.1-235.4)

Gabon	4330.4 (2245.5-7184.1)	459.7 (242.8-745.3)
Egypt	98709.5 (44337.1-174208.2)	161.7 (73-284.9)
Cook Islands	31.6 (14.5-54.8)	169.2 (78-295.1)
Croatia	10678.2 (5370.3-18652.8)	212.9 (106.8-372.3)
Mongolia	6403.9 (4076.9-9389.6)	282 (177.4-417.1)
Saudi Arabia	42766.9 (24446.8-65785)	262.7 (147-404)
Trinidad and Tobago	4386.8 (2556.3-6889.3)	370.1 (217.9-576.5)
Antigua and Barbuda	263.8 (157.2-419.5)	441.2 (265.8-694.1)
Romania	52380.7 (29410.5-83974.9)	226.5 (128.4-361.1)
Brazil	344443.4 (240210.2-488987)	230.5 (160.7-324.1)
Norway	7750.9 (5267.4-10906.2)	176.8 (121-248.6)
Yemen	31628.4 (14870.2-51966.4)	189.1 (94-310.4)
Latvia	5511.3 (3496.5-8269.8)	207.2 (132.8-308.3)
El Salvador	11873.8 (5918.7-19818.9)	214.6 (109.4-360.2)
Chile	32752.2 (17263.5-56113.6)	242.2 (127-411.9)
Liberia	8993.2 (5276.9-14557)	381.9 (230.1-601.4)
Guatemala	32525.8 (20083.6-51244.6)	373.8 (228.8-593.7)
Nepal	71338.2 (42133.1-111244.4)	380.7 (230.8-568.1)
Lithuania	9084 (5901-13753.7)	244.3 (159.4-369.5)
Armenia	5236.9 (2655.8-8967.6)	150.9 (76-258.2)
Haiti	27960 (17432.2-42999.8)	444.2 (286.2-673.1)
Slovenia	3915.2 (2109.4-6856.1)	191.2 (102.2-334)
Andorra	75.4 (37.4-135.6)	137.2 (67.6-248.4)
Bahamas	746.1 (380.3-1250.1)	291.4 (148.2-499.6)
Sweden	10474.4 (6234.9-17004.8)	119 (70.1-193.6)
Cuba	15921.2 (7996.4-26995.8)	142.7 (71.5-241.4)
Mexico	322902.6 (238780.4-428343.6)	369 (273.9-485.6)
Sudan	55502.1 (32927-85602.8)	231.4 (139.8-350.3)
Ecuador	41513.1 (22335.1-65741.7)	421.2 (222.2-677.6)
Hungary	21118.2 (10987.8-34988.4)	200.5 (104.7-330.6)
Greenland	197.9 (109.5-307.2)	355.1 (195.3-553.3)
Grenada	277 (165-438.8)	334.1 (202.9-522.1)
Honduras	19769.7 (11833.4-31009.2)	405.5 (254.3-636.5)
Libya	8437.7 (4546.4-13322.8)	184.7 (102.9-288.8)
Colombia	98115.6 (48297.4-166495)	289.9 (142.9-497.2)
Barbados	753.2 (425.1-1224.7)	289.6 (164.4-475.3)
India	2359098 (1581157.9-3016454.1)	270 (183.6-344.7)
Ukraine	81682.6 (48784.4-130264.5)	157.7 (94.5-250.1)
Morocco	62009.2 (39212.4-96428.3)	231.9 (147.8-355.6)
Albania	10066.1 (6291.6-15543.9)	292.1 (179.7-452.4)
Rwanda	38720.3 (25787.2-56010.3)	567.5 (389.2-782.8)
Saint Lucia	530.7 (315.9-811.1)	409.4 (251-618.2)
Brunei Darussalam	657.7 (313.5-1090.3)	252.1 (118-427.8)

New Zealand	6080.5 (3893.6-9161.7)	176.5 (113.1-267.3)
Kuwait	2889.9 (1384.1-5350.8)	165.1 (78.1-307)
Tunisia	15599.5 (9456.7-23433.1)	172 (104.3-254.8)
Serbia	21872 (11832-35208.4)	227.2 (121.8-367.4)
Palestine	5443.5 (3420.4-8205.6)	237.9 (150.1-360.3)
Afghanistan	46304.5 (28105.2-72552.6)	440.2 (271.7-696.7)
Iraq	38019.8 (22702.2-58904.1)	185.8 (111.3-285.5)
Maldives	515.5 (219.1-941.8)	230.1 (97.6-419.6)
Ethiopia	254125 (179995.6-328611.8)	559 (403.8-707.6)
Democratic People's Republic of Korea	35003.7 (18369.7-57389.9)	166.8 (86.9-274.1)
Sri Lanka	50135.4 (26837.5-82786.9)	298.1 (161.4-487.3)
Cyprus	1032.4 (526.9-1793.6)	133.3 (68.4-232.4)
Estonia	4354.3 (2985.6-6132)	277.3 (191.8-391.2)
Nicaragua	10934.5 (5899-17448.6)	263 (138.9-422.1)
Bahrain	1347.4 (789.6-2138.9)	272.3 (156.9-434)
Russian Federation	174211.7 (129580.5-230065.6)	115.4 (86-153.7)
Bangladesh	195709.9 (106548.1-309894.5)	169.2 (92.6-265.7)
Algeria	64725 (36221.8-106609.6)	228.4 (129.1-371.7)
Lebanon	7234.8 (4458.7-10844.7)	236.9 (147.8-352.3)
Jordan	7202 (4187.9-11163.6)	175.6 (102.5-269.2)
Bolivia (Plurinational State of)	25856.4 (14398.9-41051.8)	400.4 (221.2-637.5)
Republic of Moldova	10927.9 (7320.9-15573.3)	245.6 (164.5-350)
Bermuda	145.6 (71.1-260.8)	238 (113.5-438.4)
Peru	68201.5 (30640.3-116872.7)	310.1 (137.4-526.3)
Puerto Rico	9883.4 (5607.9-16245.9)	272.4 (154.7-446.1)
Iran (Islamic Republic of)	134253.4 (96652.5-177111.4)	202.5 (151-264.5)
United Arab Emirates	5756.9 (2924.9-9152.4)	304.7 (150.7-494.3)
Republic of Korea	83001.5 (46878.9-126734.9)	183.3 (101.8-285)
Saint Kitts and Nevis	192.3 (110.9-295.2)	485.2 (285.6-739)
China	2134558.1 (1717519.1-2651182.6	178.6 (143.4-220.6)
Belarus	18903.2 (11205.1-30793.2)	180.6 (107.5-296.4)
Qatar	949.9 (436.7-1654.7)	217.3 (100.2-378.2)
Turkey	216987.4 (137430.6-325093.2)	355.3 (226.7-529)

Num_2021	ASR_2021	AAPC	
5435.7 (3111-8029.3)	290.4 (165.7-427.6)	1.05	0.89 - 1.213
131713.4 (90300.1-178066.2)	746.4 (505.8-1031.4)	0.97	0.804 - 1.134
49271.6 (27740.7-79902.2)	350.9 (206.9-522.6)	0.84	0.745 - 0.926
2 (1.1-3.3)	216.8 (140.8-311.5)	0.75	0.433 - 1.059
16216.8 (9476.2-25104.6)	305.2 (170.1-480.8)	0.7	0.531 - 0.862
118512 (78451.3-183534.9)	87 (56.7-135.8)	0.53	0.257 - 0.805
4528.9 (2629.1-6973.2)	339.8 (202.7-542.7)	0.52	-0.478-1.532
6552.7 (4041.8-9526.5)	316.9 (201.2-452.6)	0.52	0.025 - 1.007
99 (49.9-155.4)	195.8 (104.3-317.1)	0.51	0.207 - 0.814
70929.1 (54620.3-95397.6)	174.3 (126.5-233.6)	0.49	0.233 - 0.74
10687 (5799.9-19386.4)	95.4 (48.2-179.7)	0.45	0.263 - 0.627
61061.8 (37334.5-92318.4)	321.2 (194.4-462.6)	0.44	0.35 - 0.527
48557.6 (29545.2-72421.1)	346.5 (205.5-517)	0.41	0.202 - 0.62
50277.9 (29894.6-74373.5)	347.6 (211.6-520.4)	0.4	-0.054-0.859
75047.3 (38723.3-122220.5)	239.2 (129.5-383.3)	0.37	0.213 - 0.524
466.8 (227.6-883.9)	96.5 (46.9-187.8)	0.36	0.106 - 0.623
77136.6 (17653.4-161341.8)	79.1 (18.1-169.2)	0.36	0.273 - 0.441
582.9 (328.8-886.3)	292.2 (152.3-457.6)	0.33	0.227 - 0.439
387144.5 (260655.7-596191.9)	111.2 (70-173.7)	0.33	-0.014-0.667
21866.1 (12564.2-38145.1)	164.6 (90.4-293.7)	0.32	0.156 - 0.488
394.2 (199.4-743.7)	106.2 (53.8-205.1)	0.3	-0.055-0.662
38399.7 (21802.8-60767.2)	335.4 (201.5-527.5)	0.26	0.207 - 0.315
19022.8 (10774.1-29374.2)	183.7 (98.5-286.2)	0.25	0.213 - 0.296
2.7 (1.3-4.5)	198 (120.5-310.1)	0.25	0.015 - 0.475
23831.6 (13670.9-41579.1)	126.6 (70.1-231.5)	0.22	-0.025-0.461
81585.1 (45208.2-125924)	336.5 (187.6-530.1)	0.21	0.102 - 0.307
10797.4 (5894.4-18736.8)	113.4 (57.5-216.4)	0.18	0.045 - 0.313
3780.9 (2189.7-5644.8)	329 (195.4-508.7)	0.17	0.033 - 0.312
14177 (8269.7-23190.4)	209 (115.4-339.5)	0.17	-0.114-0.447
103018.7 (50429.5-178348.4)	152.3 (74.8-270.9)	0.16	0.055 - 0.264
552.4 (294-862.3)	182.2 (96.2-284.9)	0.16	0.064 - 0.254
196306.2 (103967.2-358293.3)	178.8 (88.8-355)	0.14	-0.097-0.374
66639.2 (47990.5-95926.7)	97.1 (65.6-148.3)	0.12	-0.213-0.45
43.4 (16.2-89.5)	103.8 (42.2-209.9)	0.1	0.022 - 0.169
209.6 (76.6-399.5)	125.5 (47.9-237)	0.09	-0.037-0.221
23445.4 (13343.7-35518.2)	304.2 (166.8-454.2)	0.09	-0.186-0.364
62584.9 (31963.9-102992.9)	315.9 (165.2-491.8)	0.08	-0.028-0.187
6618.6 (3842.1-11202.1)	183.7 (100.5-305.5)	0.07	-0.183-0.32
40126.4 (26314.2-57276.7)	490.1 (339.6-688.2)	0.05	-0.308-0.409
6881.8 (4053.7-10253.9)	392.5 (240-587.9)	0.05	-0.014-0.105
43711.5 (25170.6-69577.6)	230.3 (126.4-386.6)	0.04	-0.226-0.314
235801.1 (141572.1-344204.7)	92.7 (54.1-136)	0.03	-0.057-0.109

1305.8 (712.4-1990.7)	193.8 (108-306.8)	0.02	-0.169-0.212
98549.1 (62272.2-149869.4)	297.9 (186.3-466.5)	0	-0.309-0.308
2622.2 (1585.1-3878.8)	340.8 (197.2-506.7)	-0.02	-0.56-0.526
9515.4 (4619.6-17379.3)	228.3 (105.9-403.1)	-0.03	-0.152-0.101
87591.2 (47920.8-136614.7)	313.9 (176.3-472.1)	-0.04	-0.118-0.043
7400 (4411-11728.8)	318.3 (176.7-482.5)	-0.04	-0.099-0.016
21678.3 (13948.2-31364)	414.5 (267.6-602.2)	-0.05	-0.165-0.069
90107.2 (55452.9-135889.8)	505.5 (305.5-770.1)	-0.05	-0.113-0.014
58393.8 (31806.3-92855.7)	305.8 (174.6-484.5)	-0.05	-0.265-0.158
585260.6 (416749.3-760860.2)	288.5 (205.5-374.5)	-0.07	-0.16-0.025
145253.6 (94901.1-212901.4)	375.7 (247.1-555.6)	-0.07	-0.256-0.12
6868.6 (4002.2-12275)	129.3 (66.8-242.3)	-0.07	-0.242-0.105
7272.8 (4441-12621.6)	107 (60.6-189.6)	-0.08	-0.371-0.207
4816.9 (2967.5-7239.3)	415.5 (266.6-596.9)	-0.08	-0.296-0.131
36424.4 (19561.5-59348.7)	316.6 (180.3-492.2)	-0.09	-0.166--0.017
109.8 (62.2-167.3)	186.3 (97.2-290.2)	-0.09	-0.15--0.039
47259.6 (24996.7-89436.2)	92.3 (47.3-176.5)	-0.1	-0.29-0.084
112841.9 (74449.4-159157.4)	103.3 (70.5-144.9)	-0.11	-0.176--0.042
7817.3 (4694.6-13354.6)	126.4 (67.9-229.2)	-0.11	-0.483-0.26
170888.3 (133854.6-211241.8)	377.9 (295.6-465.8)	-0.11	-0.195--0.032
144.4 (70.7-232)	141.7 (75.7-229.2)	-0.13	-0.173--0.089
2224.6 (1187.4-3407.5)	239.8 (130.8-376.7)	-0.14	-0.203--0.077
6464 (3792.9-9910.9)	274.7 (159.1-429.9)	-0.14	-0.3-0.02
260.2 (145.7-401.1)	386.8 (225-604.4)	-0.14	-0.23--0.052
10931.2 (5837.2-19065.1)	112 (53.4-207.7)	-0.15	-0.34-0.046
28614 (18768.9-40676.1)	486.4 (329.2-688.8)	-0.15	-0.307-0.012
32844.2 (16442.6-60696.3)	126.7 (61.9-232)	-0.16	-0.445-0.124
696591.6 (544883.9-906445.4)	298.4 (227.7-395.6)	-0.17	-0.242--0.092
1603.3 (637.2-2847.6)	121.1 (45.6-221)	-0.17	-0.354-0.019
15157 (9643.2-24685.7)	119.2 (71-197.1)	-0.17	-0.735-0.399
4413 (1668.8-8878.3)	82 (34.9-166.2)	-0.19	-0.264--0.122
218.9 (117.7-369.8)	231.8 (118.4-389.8)	-0.21	-0.714-0.299
125891.6 (80953.4-190637.3)	455.7 (293.1-651.5)	-0.21	-0.361--0.066
5309.4 (2573.6-8909.1)	370.1 (185-625.8)	-0.23	-0.367--0.083
14058.3 (7103.8-23875.1)	192.5 (99.5-319.1)	-0.23	-0.322--0.139
23049.6 (11804.1-38723.2)	207 (99.5-358.3)	-0.24	-0.357--0.112
323.2 (162.7-533.6)	163.7 (85.1-268.3)	-0.26	-0.315--0.204
43 (25.1-68.4)	237.9 (134.2-361.8)	-0.27	-0.324--0.216
6126.6 (3358.9-9979.4)	211.9 (109.3-348.6)	-0.31	-0.717-0.105
53273.3 (27290.1-91945.4)	113.5 (53-205.4)	-0.31	-0.551--0.065
6323.6 (3600.8-10903.2)	176.7 (97.1-293.3)	-0.31	-0.534--0.081
9681.9 (4886.2-17333.1)	203.7 (107.1-344.6)	-0.32	-0.42--0.211
60.9 (24.6-116.5)	129.3 (53-234.5)	-0.34	-0.512--0.165

19389.7 (10140.7-33380.3)	175.1 (90.6-312.6)	-0.35	-0.554--0.146
1065.1 (556.3-2001.3)	153.5 (78.4-278.8)	-0.36	-0.535--0.179
121458.3 (67668.1-212061.8)	152.6 (83.6-292.8)	-0.38	-0.662--0.095
36966.4 (19668-71967)	93.1 (46.6-173.7)	-0.38	-0.683--0.078
10033.2 (5814.6-16094.3)	269.7 (159.3-421.7)	-0.38	-0.573--0.195
23311.7 (12676.2-37501)	312.9 (177.8-479.9)	-0.39	-0.479--0.291
42120.5 (27974.5-57781.5)	406.9 (269.1-572)	-0.39	-0.603--0.178
4431.2 (1765.6-8236.4)	106 (39.3-202.5)	-0.41	-0.537--0.289
83168 (38414.4-144433.4)	150.7 (65.5-260.5)	-0.41	-0.477--0.35
8735.9 (3448.4-15784.7)	124.1 (48.5-221.7)	-0.42	-0.506--0.334
84542.1 (56936.8-117561.5)	468.8 (329.8-652.8)	-0.43	-0.584--0.275
348.1 (215.1-546.2)	301.5 (179.4-451.7)	-0.43	-0.627--0.237
247143.8 (167802.7-355441.5)	445.3 (294.8-641.6)	-0.44	-0.598--0.283
117194.9 (65692.9-178373.8)	388.6 (208.2-604.8)	-0.44	-0.592--0.29
304.7 (173-451.5)	253.8 (151-385.7)	-0.45	-0.521--0.379
16665.1 (9436.3-28317.7)	127.8 (76.3-205.8)	-0.45	-0.588--0.316
41838.8 (15685.6-74840)	138.7 (54.2-247.8)	-0.45	-0.546--0.36
19039.8 (8156.1-33702.8)	114.4 (50-195.9)	-0.46	-0.494--0.42
24.6 (12.3-40.5)	217.3 (111.1-348.7)	-0.48	-0.563--0.39
27239.4 (15271-42105.6)	263.8 (150-417.9)	-0.48	-0.69--0.274
913.2 (543.3-1464.7)	210 (107.5-348.7)	-0.48	-0.747--0.219
1612.5 (902.9-2638.5)	287.7 (153.5-471.7)	-0.49	-0.557--0.415
21.9 (6.8-47.2)	67 (20.3-146.5)	-0.49	-0.702--0.271
11468 (6577.5-19219)	199.8 (110.2-338.9)	-0.49	-0.627--0.353
18429.9 (10358.5-28652.5)	356.2 (194.6-544.2)	-0.49	-0.675--0.308
189.5 (108.1-290.7)	184.8 (96.1-298.9)	-0.49	-0.532--0.455
20.2 (11.7-31.3)	168.3 (91.6-261.9)	-0.5	-0.55--0.444
168.6 (86.4-281.9)	146.4 (65.4-254.5)	-0.53	-0.738--0.323
10842.9 (5896.8-20409.7)	109.8 (57.3-204)	-0.54	-0.697--0.373
94758.7 (64411.2-137458.2)	363.1 (241.6-514.9)	-0.56	-0.687--0.422
264288.8 (157653.9-405479.8)	307.6 (184.2-474.2)	-0.56	-0.684--0.432
709.4 (333.6-1415)	113.9 (46.1-221.3)	-0.57	-0.682--0.458
1683.6 (999.6-2677.1)	222.5 (127-348)	-0.57	-0.812--0.329
6446.9 (3595.8-10553.2)	177.8 (96.1-291.4)	-0.57	-0.855--0.286
75319 (39807.5-123205.5)	265.1 (138.8-462.2)	-0.57	-0.791--0.354
19307.7 (13511.1-27807.3)	284.3 (179.2-428.9)	-0.57	-1.535-0.396
2937.9 (1996.6-4226.2)	419.9 (273.3-596.3)	-0.58	-1.532-0.39
1825 (1029.4-2844)	301.8 (175.6-474.2)	-0.59	-0.758--0.412
131830.6 (96443.4-175086.6)	231 (168.2-311.5)	-0.59	-0.715--0.458
48361.9 (31941.3-69448.5)	427.1 (289.8-607.8)	-0.6	-0.784--0.423
3312.4 (1808-5467.1)	158.7 (89.2-267)	-0.64	-0.837--0.435
102085.3 (70989.7-148390.3)	148.2 (101.8-221.1)	-0.68	-1.107--0.24
28680 (14928.1-53737.7)	109.4 (55.7-209.2)	-0.68	-0.926--0.434

6765.5 (3650.1-10836.1)	374.9 (194.3-615.7)	-0.69	-0.883--0.492
136001.4 (69004.3-223467.8)	132.7 (59.7-240.4)	-0.7	-0.81--0.59
23.5 (9.5-42.4)	136 (62.4-247.2)	-0.7	-0.797--0.604
7689 (3870.6-13654.3)	172.1 (83.8-318.5)	-0.7	-1.017--0.389
7385.5 (4366.8-11762.6)	223.4 (116.4-364.4)	-0.71	-0.951--0.467
77490.1 (38650.1-132922.4)	211.7 (99-386.4)	-0.71	-0.809--0.617
4159 (2167.9-6801.3)	287.4 (153.6-486.5)	-0.73	-1.141--0.323
323.6 (189.4-497.7)	322.8 (184.9-523.2)	-0.75	-1.103--0.391
33992.8 (19172.8-58419.5)	180.1 (100.7-290.7)	-0.75	-1.132--0.363
405961.2 (304730.7-534745.4)	182.6 (129.9-254.6)	-0.76	-0.879--0.649
8462.6 (5610.6-12736.8)	142.9 (91.5-227.9)	-0.77	-1.082--0.449
52226.4 (29979.5-79341.5)	148.2 (79.9-235.6)	-0.78	-0.915--0.649
3152.8 (1722.2-5238.9)	161.8 (86.2-286.3)	-0.81	-1.345--0.268
10869.8 (4920.1-20375.8)	165.9 (74.2-292.8)	-0.83	-0.999--0.651
34919.1 (18076.6-61334.3)	183.6 (92.4-331.9)	-0.85	-0.943--0.764
13946.4 (7458.9-22759.2)	290.5 (165.4-431.6)	-0.86	-1.202--0.512
47515.9 (27364.3-75178.3)	284.5 (156.4-453.6)	-0.87	-1.386--0.342
82769.5 (52384.7-128523.9)	290.5 (185.4-435.2)	-0.88	-0.957--0.806
5431.9 (3074.9-8819.9)	190.1 (110.8-303)	-0.9	-1.264--0.534
3413.5 (1544.1-6088.7)	120.5 (57.7-211.4)	-0.91	-1.218--0.593
40547.9 (25807.2-58299.1)	323.5 (202.6-482)	-0.94	-1.03--0.843
2969.9 (1543.1-5241.1)	140.9 (74.9-247)	-0.94	-1.251--0.626
94.4 (42-180.8)	103.6 (46.1-201.8)	-0.96	-1.193--0.716
869.3 (415.9-1444.6)	219.1 (101.5-367.3)	-0.99	-1.309--0.665
9782.3 (5970.1-15907.3)	87.4 (49.7-149.7)	-1	-1.527--0.469
11853.3 (6059.2-21370.3)	103.6 (47.7-190.5)	-1.02	-1.161--0.869
334749.7 (244619.8-448407.2)	268.5 (192-360.9)	-1.02	-1.161--0.875
79508.5 (46524.8-119608.5)	167.7 (101.5-256.7)	-1.02	-1.114--0.927
54960.3 (27608.5-94405.2)	313.9 (155-524.7)	-1.02	-1.674--0.364
14606.8 (8382.2-25998.6)	148.5 (74.7-266.4)	-1.05	-1.53--0.562
157.5 (85.5-255.4)	250.9 (135.4-429.7)	-1.05	-1.264--0.831
260 (141.8-418.3)	240.1 (128.5-393)	-1.07	-1.569--0.563
28522.1 (16043.1-45855.3)	292.5 (164-456.7)	-1.07	-1.146--0.989
8758.7 (4730.8-14392.3)	131.7 (66.9-218.5)	-1.09	-1.248--0.927
103081.2 (50738.6-185481.8)	206.3 (98.7-371.2)	-1.09	-1.431--0.747
651.4 (347.9-1067.7)	199.8 (105-335.7)	-1.09	-1.519--0.661
2786484.4 (2107620.5-3427937.4	191.6 (146.9-237.7)	-1.1	-1.238--0.954
51175 (29077.1-85672.5)	115.8 (62-194)	-1.11	-1.741--0.472
60886.5 (36228.1-98715.7)	168.5 (97.3-273.8)	-1.12	-1.242--0.999
5762.6 (3399.6-9468)	215.2 (123.5-367.3)	-1.12	-1.3--0.947
49379.8 (31754.7-69183.4)	405.8 (276-585.1)	-1.13	-1.748--0.509
530.2 (313.4-839.7)	279.8 (158.3-458.5)	-1.15	-1.446--0.856
829.3 (410.6-1438.1)	174.6 (85.5-292.4)	-1.17	-1.254--1.095

6064 (3821.3-9912.4)	121.5 (70.5-199)	-1.18	-1.56--0.788
4650.1 (1990-9178.5)	117.9 (46.4-231.6)	-1.23	-1.618--0.833
13445 (7200.6-22922.4)	118.1 (61.6-205.2)	-1.24	-1.283--1.187
15101.5 (8161.2-25667.2)	153.2 (81-270.2)	-1.27	-1.507--1.024
8505.1 (5212.7-13348.9)	161.7 (95.1-252.5)	-1.27	-1.532--1.007
95632.1 (56179.6-143780.9)	293.1 (182.3-448.9)	-1.28	-1.6--0.949
52680.9 (26680.1-91061.5)	124.7 (62.6-208.7)	-1.3	-1.418--1.177
713.6 (330.4-1250.4)	154 (67-275.2)	-1.3	-1.44--1.16
369418.3 (299947.5-460092.5)	372.6 (302.9-464)	-1.3	-1.366--1.236
28278.3 (16026.8-46042.9)	110 (60.7-183)	-1.31	-1.413--1.199
44286.6 (21350.9-74139.1)	197.1 (87.7-345.7)	-1.35	-1.48--1.226
1234.7 (618.2-2398)	87.6 (39.4-164.6)	-1.38	-1.604--1.149
2505.5 (1326.8-4295.7)	177.5 (97.6-298.5)	-1.38	-1.708--1.051
11995.8 (6313.2-20864.8)	170.5 (85.6-302.2)	-1.39	-1.492--1.283
2509.7 (1295.8-4202.2)	178.6 (92.2-310.9)	-1.4	-1.61--1.179
106249 (70691.9-160021.6)	72.8 (48.2-110.6)	-1.46	-2.157--0.748
169469.6 (91823.5-276962.3)	108 (57.9-184)	-1.5	-1.649--1.348
62310.4 (33353.2-102576.9)	139.9 (73-230.3)	-1.55	-1.593--1.514
8144.5 (5032.8-13565.6)	149.2 (83.7-244.1)	-1.56	-1.756--1.355
12714.1 (6431.3-21975.2)	109.2 (53.4-193)	-1.56	-1.752--1.369
28899 (15609.5-46509.2)	242.8 (126.1-392.9)	-1.59	-1.619--1.561
5611.5 (3702.4-8364.6)	149.3 (93.5-228.1)	-1.6	-1.935--1.267
96.7 (40.5-192.5)	144.5 (60.9-281.6)	-1.62	-1.752--1.494
66354.6 (28774.6-129820.2)	189.3 (75.8-353.7)	-1.62	-1.821--1.426
5946.4 (2833.9-11164.4)	164.6 (74.3-304.3)	-1.64	-1.85--1.423
103039.3 (74862.5-143597.4)	121.5 (81.7-172.7)	-1.65	-1.741--1.554
15716.6 (6336.7-28753.1)	177.4 (74.6-330.1)	-1.67	-1.877--1.458
55339.1 (27547.6-98279.4)	108.7 (51.9-201.4)	-1.69	-1.798--1.572
162.6 (82.2-262)	269.3 (142.6-448.3)	-1.77	-2.396--1.144
1300816 (952460.6-1771011.8)	101.4 (72.5-139.4)	-1.79	-1.96--1.619
9803.8 (5444.9-16707.5)	99 (52.2-175.6)	-1.9	-2.277--1.526
3153.5 (1377.3-5983.3)	119.1 (48.4-232.5)	-1.93	-2.068--1.781
158628 (92877.5-253054.2)	193.5 (111.2-316.2)	-1.95	-2.156--1.739

Text S1. Explanation of authorship change statement

During the manuscript submission process, due to the authors' oversight, the designations of co-first authors were not clearly indicated, although this was their original intention. The authors regret this omission and any inconvenience it may have caused. QC made substantial contributions to this work, including through his major involvement in data analysis, drafting of the initial manuscript, and significant contributions to the subsequent revisions. The authors acknowledge this lack of clarity as their responsibility and sincerely apologize for the confusion. All authors have reviewed and agreed to this correction, and confirm that the requested changes accurately reflect each author’s contributions in accordance with the Journal’s authorship policies.
